# CYP4X1/sEH‐Dependent Endocannabinoid Metabolism Drives Fibroblast‐Mediated Immunosuppression to Limit Immunotherapy in Colon Cancer

**DOI:** 10.1002/advs.202507695

**Published:** 2025-11-23

**Authors:** Min Mo, Xuewei Chen, Yanzhuo Liu, Chenlong Wang, Xuehan Chen, Nan Zhang, Nan He, Ying Li, Jingyi Wang, Honglei Chen, Jing Yang

**Affiliations:** ^1^ Department of Pharmacology and Hubei Province Key Laboratory of Allergy and Immune‐related Diseases School of Basic Medical Sciences Wuhan University Wuhan 430071 China; ^2^ Department of Laboratory Medicine Zhongnan Hospital of Wuhan University Wuhan 430071 China; ^3^ Department of Pharmacy Renmin Hospital of Wuhan University Wuhan 430071 China; ^4^ Department of Pharmacy Zhongnan Hospital of Wuhan University Wuhan 430071 China; ^5^ Department of Pathology School of Basic Medical Sciences Wuhan University Wuhan 430071 China

**Keywords:** CAFs, colon cancer, CYP4X1, EPHX2, immunotherapy

## Abstract

Cytochrome P450 (CYP) 4X1 and soluble epoxide hydrolase (sEH), the key enzymes responsible for endocannabinoid oxidative metabolism, have been implicated in inflammation and cancer. However, the precise role of CYP4X1 and sEH in tumor immune evasion is poorly understood. Here, it is elucidated that CYP4X1/sEH‐dependent endocannabinoid metabolism governs immune evasion in colon cancer by promoting the infiltration of regulatory T cells (Tregs) and impairing CD8^+^ T cell effector function. Mechanistically, CYP4X1/sEH‐derived 14,15‐EET‐EA upregulates PD‐L1, CXCL12, and TGF‐β in cancer‐associated fibroblasts (CAFs) via the GPR119‐Gs/β‐arrestin 2 signaling axis. Importantly, targeted regulation of the CYP4X1/sEH‐GPR119 axis enhances the efficacy of anti‐PD‐1 therapy. Moreover, CYP4X1 and sEH levels jointly predict prognosis and immune infiltration in human colon cancer. Together, this study highlights that CYP4X1/sEH‐dependent endocannabinoid metabolism controls CAF‐mediated immune evasion, and targeting the CYP4X1/sEH‐14,15‐EET‐EA‐GPR119 axis represents a promising therapeutic strategy for improving anti‐PD‐1 therapy in colon cancer.

## Graphical abstract

A proposed mechanism to explain the role of CYP4X1/sEH‐14,15‐EET‐EA system in colon cancer immune escape. 14,15‐EET‐EA, 14,15‐epoxyeicosatrienoic acid‐ethanolamide; sEH, soluble epoxide hydrolase; CYP4X1, cytochrome P450 4X1; GPR119, G‐protein coupled receptor 119; EGFR, epidermal growth factor receptor; Gs, Gαs subunit; cAMP, cyclic adenosine monophosphate; CXCL12, C‐X‐C motif chemokine 12; TGF‐β, transforming growth factor‐β; PD‐L1, programmed death ligand 1; CAFs, cancer‐associated fibroblasts.

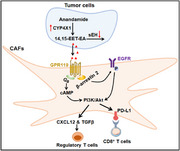

## 1 Introduction

Colon cancer ranks third among the most frequently diagnosed cancers worldwide.^[^ ^1^ ^]^ The treatment of colon cancer has been revolutionized by the introduction of immune checkpoint inhibitors (ICIs).^[^ ^2^ ^]^ However, the clinical use of ICIs therapy in colon cancer remains highly limited since only a select subset of patients are responsive.^[^ ^2^ ^]^ One reason for the low response rate is that tumors establish a highly immunosuppressive microenvironment to evade immune surveillance.^[^ ^2^ ^]^ Therefore, elucidating the molecular mechanisms of immunosuppressive tumor microenvironment (TME) formation to improve current immunotherapy is critical for the treatment of colon cancer.

Cannabis is commonly used to alleviate cancer symptoms, including pain.^[^ ^3^ ^]^ Nevertheless, cannabis consumption compromises immune function^[^ ^4^ ^]^ and attenuates the response to immunotherapy in cancer patients.^[^ ^5^ ^]^ The endocannabinoid system (ECS) is composed of endocannabinoids (e.g., anandamide), enzymes catalyzing the degradation and biosynthesis of endocannabinoids, and cannabinoid receptors (CBs).^[^ ^6^ ^]^ The intratumoral levels of endocannabinoids, including anandamide (AEA), are increased in patients with colon cancer.^[^ ^7^ ^]^ The AEA inhibits anti‐tumor immunity by impairing the function of CD8^+^ T cells, and thereby diminishing the therapeutic efficacy of PD‐1 antibody in colon cancer.^[^ ^8^ ^]^ Conversely, anandamide exerts growth‐inhibitory effects on colon cancer cells through metabolism catalyzed by COX‐2.^[^ ^9^ ^]^ The activation of the classical endocannabinoid receptor CB_2_ limits the anti‐tumor activity of CD8^+^ T cells in colon cancer, while depletion of CB_2_ sensitizes lung cancer to anti‐PD‐1 therapy.^[^ ^10^, ^11^ ^]^ Controversially, another study reported that CB_2_ activation suppresses colon tumorigenesis.^[^ ^12^ ^]^ Previous attention has been paid to the key enzymes of endocannabinoid catabolism, including fatty acid amide hydrolase and monoacylglycerol lipase.^[^ ^13^, ^14^ ^]^ However, the clinical studies with their inhibitors have been a disappointment, with several studies showing a negative outcome or safety concerns.^[^ ^15^ ^]^ Therefore, the precise role of ECS in tumor immune escape remains unclear.

Cytochrome P450 (CYP) 4X1, an orphan enzyme, oxidatively metabolizes AEA into 14,15‐epoxyeicosatrienoic acid (EET)‐ethanolamide (EA) that is then catabolized by soluble epoxide hydrolase (sEH, encoded by *EPHX2* gene).^[^ ^16^ ^]^ The *CYP4X1* gene is upregulated, whereas the *EPHX2* gene is downregulated in human colon cancer tissues.^[^ ^17^, ^18^ ^]^ Low *EPHX2* expression predicts a poor prognosis in patients with colon cancer.^[^ ^19^ ^]^ Our previous study has demonstrated that CYP4X1‐catalyzed AEA metabolite 14,15‐EET‐EA is implicated in tumor growth and angiogenesis.^[^ ^20^ ^]^ GPR119, a non‐classical endocannabinoid receptor, is activated by 14,15‐EET‐EA.^[^ ^21^ ^]^ GPR119 is involved in the induction of regulatory T cells (Tregs) in high‐fat diet‐induced obese mice.^[^ ^22^ ^]^ However, the role of CYP4X1/sEH‐14,15‐EET‐EA‐GPR119 axis in tumor immune evasion remains fully unknown.

Herein, we discovered that CYP4X1/sEH‐dependent endocannabinoid metabolism drives immunosuppressive TME formation, and consequently resistance to immunotherapy in colon cancer. Mechanistically, CYP4X1/sEH‐derived 14,15‐EET‐EA promotes the infiltration of Tregs and impairs CD8^+^ T cell effector function by the upregulation of PD‐L1, CXCL12, and TGF‐β in cancer‐associated fibroblasts (CAFs). Importantly, simultaneous targeting of CYP4X1 and sEH orchestrates a favorable antitumor TME and potentiates the efficacy of anti‐PD‐1 therapy. Our findings improve understanding of CYP4X1/sEH‐catalyzed endocannabinoid metabolism in the establishment of an immunosuppressive TME, and may provide strategies for sensitizing immunotherapy in human colon cancer.

## 2 Results

### 2.1 CYP4X1 and sEH Jointly Predict Prognosis in Human Colon Cancer

**Figure 1:**
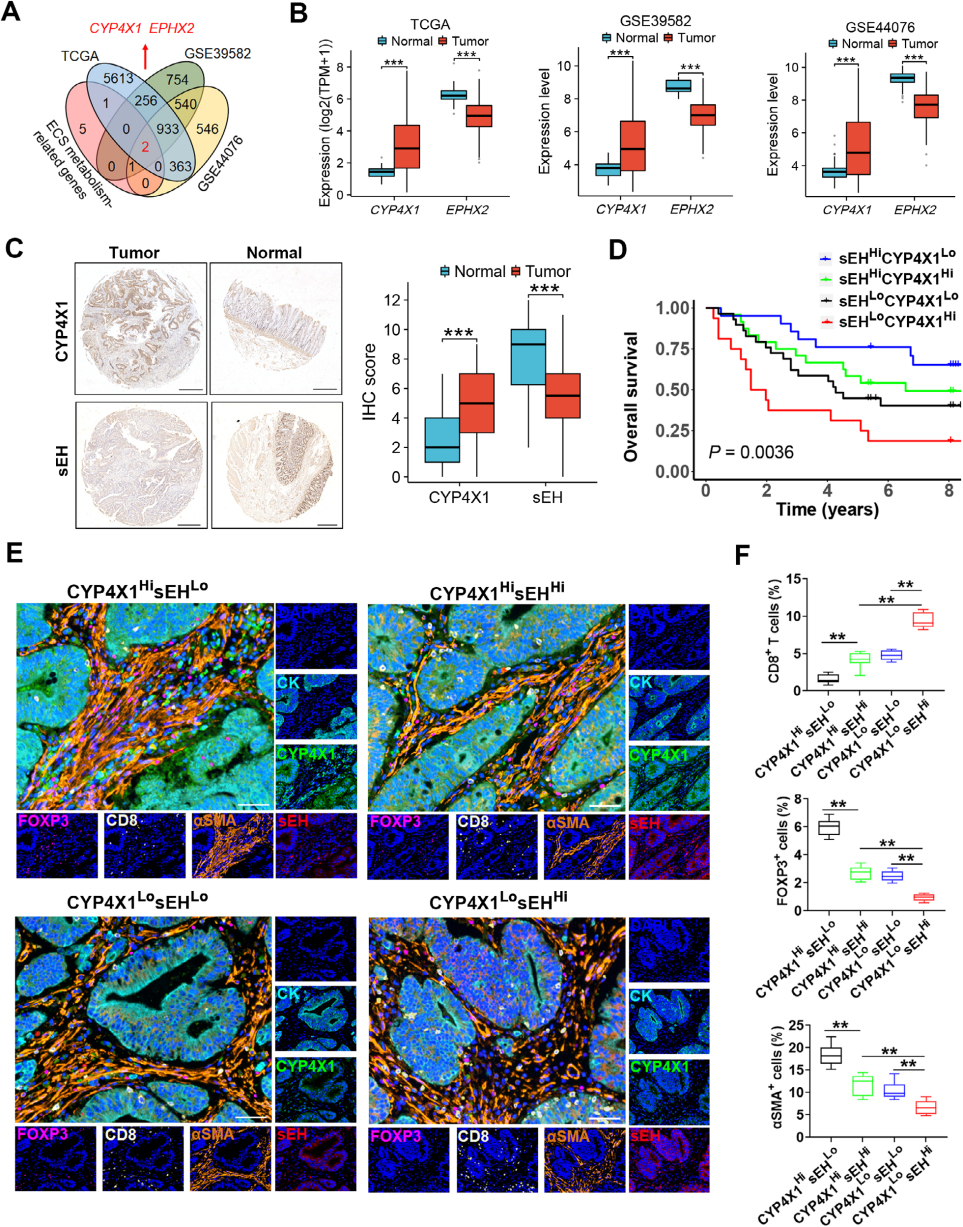
CYP4X1 and sEH jointly predict prognosis and immune infiltration in colon cancer. A) Venn diagram showing the endocannabinoid metabolism‐related differentially expressed genes (DEGs) in the TCGA colon adenocarcinoma (TCGA‐COAD) and colon cancer‐related GEO datasets (GSE39582 and GSE44076). B) *CYP4X1* and *EPHX2* gene expression levels in human colon cancer tissues and normal tissues from the TCGA and GEO databases (GSE39582 and GSE44076). C) Representative immunohistochemistry (IHC) images and IHC scores of CYP4X1 and sEH staining in human colon carcinoma tissue microarrays (*n* = 90). Scale bar, 500 µm. D) Kaplan‐Meier survival curves of overall survival for patients with colon cancer in the high CYP4X1 and low sEH expression (CYP4X1^Hi^sEH^Lo^), high CYP4X1 and high sEH expression (CYP4X1^Hi^sEH^Hi^), low CYP4X1 and low sEH expression (CYP4X1^Lo^sEH^Lo^), and low CYP4X1 and high sEH expression (CYP4X1^Lo^sEH^Hi^) groups stratified by the median expression of CYP4X1 and sEH. E) Representative images of multiplex immunofluorescence (mIF) staining among four groups stratified by the median expression of CYP4X1 and sEH. Scale bar, 50 µm. F) Quantification of CD8^+^ T cells, FOXP3^+^ Tregs, and α‐SMA^+^ CAFs as a proportion of total cells (*n* = 8). Data are shown as mean ± SEM. *P* values were determined using Mann‐Whitney tests (B), Wilcoxon matched‐pairs signed‐rank tests (C), log‐rank test (D), or one‐way ANOVA (F). ^**^ *P* < 0.01; ^***^ *P* < 0.001.

The differentially expressed genes (DEGs) between tumor and normal tissue samples were identified in the TCGA colon adenocarcinoma (TCGA‐COAD) cohort (455 colon cancer and 41 normal samples) and the GEO database (443 colon cancer and 19 normal samples in GSE39582; 98 colon cancer and 148 normal samples in GSE44076). The DEGs were intersected with the endocannabinoid oxidative metabolism‐related genes, including *CYP4X1*, *CYP2D6*, *CYP3A4*, *CYP2J2*, *CYP4F2*, *ALOX12*, *ALOX15*, *PTGS2*, and *EPHX2*.^[^ ^16^ ^]^ We obtained two common genes, *CYP4X1* and *EPHX2* (**Figure** **1**A). The *CYP4X1* gene was upregulated, whereas the *EPHX2* gene was downregulated in human colon cancer tissues as compared with adjacent normal tissues (TCGA, GSE39582, and GSE44076; Figure 1B). Similarly, CYP4X1 protein was overexpressed, while *EPHX2*‐encoded sEH protein was repressed in tumor tissues as compared to the corresponding para‐carcinoma normal tissues in a tissue microarray of human colon cancer (*n* = 90) (Figure 1C). High *CYP4X1* gene expression (*CYP4X1* ^Hi^) tended to have poor overall survival (OS) in patients with colon cancer, although the difference did not reach significance (Figure S1A, Supporting Information). *CYP4X1* ^Hi^ was correlated with shorter recurrence‐free survival (RFS; Figure S1A, Supporting Information). Obviously, patients with high *EPHX2* gene expression (*EPHX2* ^Hi^) had longer OS and disease‐free survival (DFS) than those with low *EPHX2* gene expression (*EPHX2* ^Lo^) (Figure S1B, Supporting Information). Similar results were obtained by analyzing the expression microarray data from 90 patients with colon cancer (Figure S1C, Supporting Information). Next, the colon cancer patients with *EPHX2* ^Lo^ were stratified into two subgroups based on the *CYP4X1* gene level. Surprisingly, *CYP4X1* ^Hi^ significantly predicted poorer OS as compared to low *CYP4X1* gene expression (*CYP4X1* ^Lo^) in colon cancer patients with *EPHX2* ^Lo^ (TCGA‐COAD, GSE39582, and GSE17538; Figure S1D,E, Supporting Information). To further decipher the combined impact of CYP4X1 and sEH on prognosis, the patients with colon cancer were stratified into four subtypes based on CYP4X1 and sEH levels. We found that patients with CYP4X1^Lo^ in conjunction with sEH^Hi^ had the best prognosis, whereas those with CYP4X1^Hi^ accompanied by sEH^Lo^ showed the most unfavorable OS in human colon cancer tissue microarray and GSE17538 dataset (Figure 1D; Figure S1F, Supporting Information). Interestingly, fecal *Fusobacterium nucleatum* (*F. nucleatum*) abundance in patients with colorectal cancer was significantly higher than that in healthy individuals, and treatment of HCT116 colon cancer cells with *F. nucleatum* resulted in an upward trend in *CYP4X1* gene expression, while *EPHX2* gene expression was significantly downregulated (GSE141805), suggesting that gut microbiota *F. nucleatum* may regulate *CYP4X1* and *EPHX2* (Figure S2, Supporting Information). These results indicate that CYP4X1 and sEH jointly predict prognosis in human colon cancer.

### 2.2 CYP4X1 and sEH Jointly Predict Immunosuppression in Human Colon Cancer

The tumor immune microenvironment innately modulates tumor progression.^[^ ^23^ ^]^ We found that the *CYP4X1* gene was positively correlated with Tregs (TCGA‐COAD), CAFs (GSE33193), and endothelial cells (GSE33193), but negatively correlated with CD8^+^ T cells (GSE64857) in human colon cancer (Figure S3A, Supporting Information). Moreover, higher proportions of immunosuppressive cells, including Tregs, CAFs, and endothelial cells, but less infiltration of CD8^+^ T cells, were noticed in the *EPHX2* ^Lo^ group compared to the *EPHX2* ^Hi^ group (GSE41258 and TCGA‐COAD; Figure S3B, Supporting Information). Single‐cell RNA sequencing (scRNA‐seq) datasets of human colorectal cancer displayed that *CYP4X1* and *EPHX2* were mainly enriched in tumor cells (Figure S3C,D, Supporting Information). Next, multiplex immunofluorescence (mIF) staining was performed to clarify the relationships of CYP4X1/sEH with the immune landscape in human colon cancer. We observed an apparent colocalization of CYP4X1 and sEH with CK, indicating that CYP4X1 and sEH were expressed in tumor cells (Figure S4A,D, Supporting Information). Tumors with CYP4X1^Lo^ generally exhibited relatively fewer FOXP3^+^ cells (Tregs) and CD31^+^ cells (endothelial cells) but more CD8^+^ T cells than those with CYP4X1^Hi^ (Figure S4A,B, Supporting Information). Moreover, the CAF level was significantly higher in the CYP4X1^Hi^ group as compared with the CYP4X1^Lo^ group (Figure S4C, Supporting Information). Conversely, the human colon cancer tissues with sEH^Hi^ had more infiltration of CD8^+^ T cells but lower proportions of Tregs, CAFs, and endothelial cells compared with sEH^Lo^ (Figure S4D,E, Supporting Information). To decipher the combined impact of CYP4X1 and sEH on immunosuppressive TME in human colon cancer, the patients with colon cancer were stratified into four subtypes based on CYP4X1 and sEH protein contents. As expected, the patients with CYP4X1^Lo^sEH^Hi^ signature displayed the highest proportions of CD8^+^ T cells and the least infiltration of Tregs and CAFs, indicative of a “hot” tumor microenvironment (Figure 1E,F). Human colon cancer tissues with CYP4X1^Lo^sEH^Hi^ signature also showed the least tumor microvessel density and the strongest cytotoxic activity of CD8^+^ T cells (Figure S5, Supporting Information). These data suggest that CYP4X1^Hi^ and sEH^Lo^ are correlated with an immunosuppressive TME in human colon cancer.

### 2.3 CYP4X1/sEH‐14,15‐EET‐EA System Induces Immunosuppression in Colon Cancer

**Figure 2:**
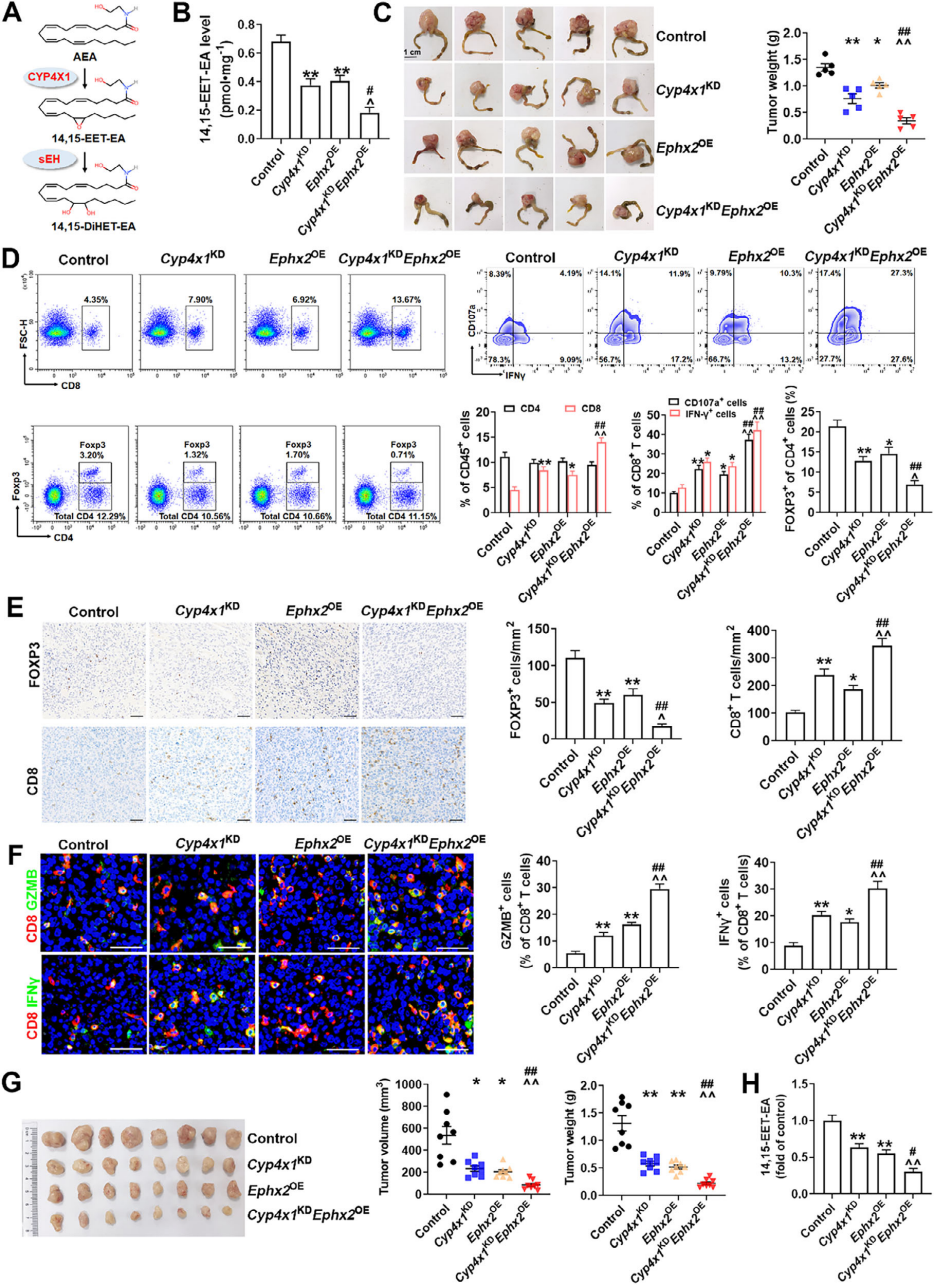
CYP4X1 and EPHX2 manipulate the immune microenvironment in murine colon cancer models. A) Biochemistry of the CYP4X1‐sEH pathway. AEA, anandamide; 14,15‐EET‐EA, 14,15‐epoxyeicosatrienoic acid‐ethanolamide; 14,15‐DiHET‐EA, 14,15‐dihydroxyeicosatrienoic acid‐ethanolamide. B) MC38 tumor chunks with *Cyp4x1* knockdown (*Cyp4x1* ^KD^), *Ephx2* overexpression (*Ephx2* ^OE^), or their combination (*Cyp4x1* ^KD^ *Ephx2* ^OE^) were orthotopically implanted into C57BL/6 mice (*n* = 5). 14,15‐EET‐EA level was determined by liquid chromatography tandem‐mass spectrometry (LC‐MS/MS). 14,15‐EET‐EA level was reported as pmol mg^−1^ of wet tissue weight. C) Representative ex vivo images of orthotopic MC38 tumors and tumor weights. D) Representative flow staining and quantification of CD8^+^ T cells, CD4^+^ T cells, Tregs, CD107a^+^ CD8^+^ T cells, and IFN‐γ^+^ CD8^+^ T cells in tumor tissues of the indicated groups. E) IHC staining and quantification of CD8 and FOXP3 in the indicated tumor tissues. Scale bar, 50 µm. F) Representative images and quantitative analysis of immunofluorescence (IF) staining of CD8 (red) and GZMB (green) or IFN‐γ (green) in tumor tissues. Scale bar, 50 µm. G) CT26 colon cancer cells with *Cyp4x1* ^KD^, *Ephx2* ^OE^, or *Cyp4x1* ^KD^ *Ephx2* ^OE^ expression were subcutaneously implanted into BALB/c mice (*n* = 8). Tumor weights and tumor volumes were presented. H) 14,15‐EET‐EA level was determined by LC‐MS/MS. Data are presented as mean ± SEM. *P* values were determined using one‐way ANOVA. ^*^ *P* < 0.05 and ^**^ *P* < 0.01 vs. control; ^^^ *P* < 0.05 and ^^^^ *P* < 0.01 vs. *Cyp4x1* ^KD^; ^#^ *P* < 0.05 and ^##^ *P* < 0.01 vs. *Ephx2* ^OE^ group.

**Figure 3:**
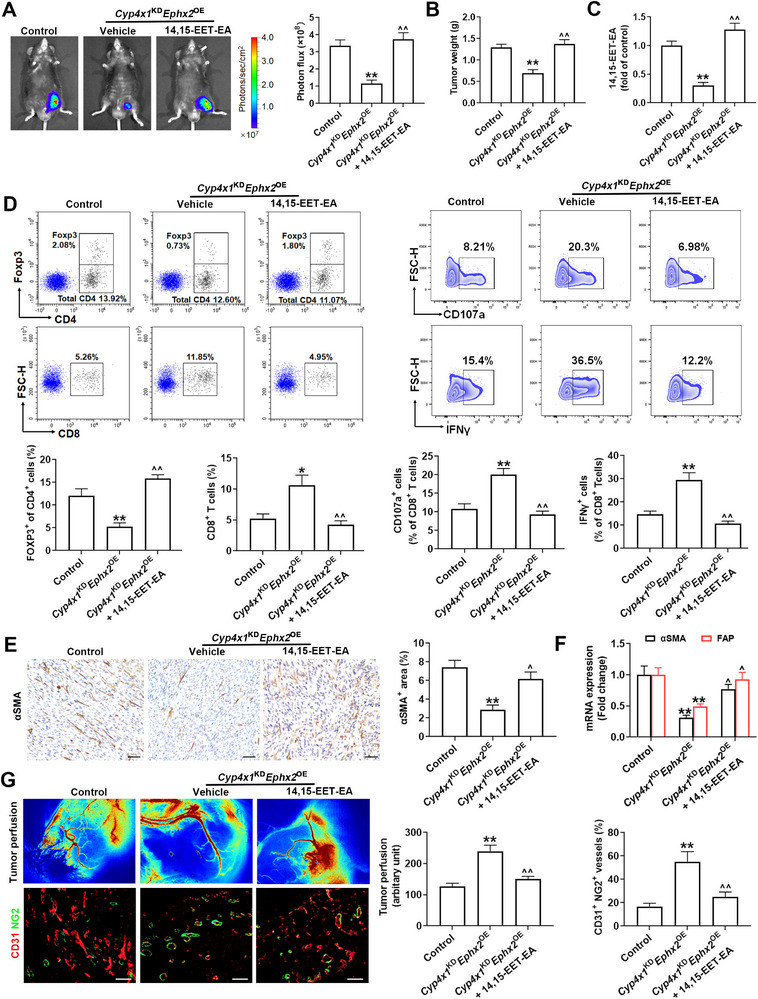
14,15‐EET‐EA supplementation restricts *Cyp4x1* ^KD^ *Ephx2* ^OE^‐mediated anti‐tumor immunity. A) Representative bioluminescence images of C57BL/6 mice bearing MC38 tumors with or without 14,15‐EET‐EA treatment. Signal intensity was measured as photon flux (photons/second) and coded to a color scale (*n* = 8). B) Tumor weights were measured for the indicated groups (*n* = 8). C) 14,15‐EET‐EA level in tumor tissues was determined by LC‐MS/MS (*n* = 8). D) The percentages of Tregs, CD8^+^ T cells, CD107a^+^ CD8^+^ T cells, and IFN‐γ^+^ CD8^+^ T cells were determined by flow cytometry (*n* = 5). E) Representative IHC staining and frequency analysis of αSMA expression in tumor tissues (*n* = 5). Scale bars, 50 µm. F) αSMA and FAP mRNA levels in the purified CAFs (*n* = 5). G) Tumor perfusion and IF analysis of tumor vessel normalization in the indicated groups (*n* = 5). Scale bar, 50 µm. Data are presented as mean ± SEM. *P* values were determined using one‐way ANOVA. ^*^ *P* < 0.05 and ^**^ *P* < 0.01 vs. control; ^^^ *P* < 0.05 and ^^^^ *P* < 0.01 vs. *Cyp4x1* ^KD^ *Ephx2* ^OE^ group.

CYP4X1 is a critical metabolic node that initiates the formation of 14,15‐EET‐EA from AEA, which is then catabolized by sEH (**Figure** **2**A).^[^ ^16^ ^]^ We constructed *Cyp4x1*‐knockdown (*Cyp4x1* ^KD^) and/or *Ephx2*‐overexpression (*Ephx2* ^OE^) MC38 orthotopic xenograft model in C57BL/6 mice, and found that *Cyp4x1* ^KD^ significantly reduced 14,15‐EET‐EA level in tumor tissues, accompanied by a non‐significant increasing trend in its precursor AEA (Figure 2B; Figure S6A, Supporting Information). Moreover, *Cyp4x1* ^KD^ did not significantly alter the levels of other N‐acylethanolamines (NAEs), including oleoylethanolamide (OEA) and palmitoylethanolamide (PEA) (Figure S6A, Supporting Information). Importantly, *Cyp4x1* ^KD^ or *Ephx2* ^OE^ significantly hindered orthotopic tumor growth, restrained the infiltration of Tregs, and promoted the expansion of CD8^+^ T cells, along with their expression of IFNγ, GZMB, and CD107a, without influencing the levels of CD4^+^ T cells (Figure 2C–F). The combination (*Cyp4x1* ^KD^ *Ephx2* ^OE^) of both resulted in a superior anti‐tumor immune response and greatly decreased 14,15‐EET‐EA level compared with monotherapy (Figure 2B–F). CAFs and vascular abnormalities regulate the tumor‐infiltrating lymphocyte population in the TME and facilitate immune evasion.^[^ ^24^, ^25^ ^]^ We found that *Cyp4x1* ^KD^ or *Ephx2* ^OE^ markedly diminished CAF activation and promoted vascular normalization (Figure S6B,C, Supporting Information). Unsurprisingly, the combination of *Cyp4x1* ^KD^ and *Ephx2* ^OE^ was more efficient in inhibiting CAF activation and inducing vascular normalization than either *Cyp4x1* ^KD^ or *Ephx2* ^OE^ alone (Figure S6B,C, Supporting Information). Consistent results were obtained in the subcutaneous CT26 syngeneic mouse model (Figure 2G,H; Figure S6D–I, Supporting Information). We next explored the therapeutic potential of a CYP4X1 inhibitor and a sEH inducer in the MC38 colon cancer model. Similarly, the combination of CYP4X1 inhibitor CH625 and sEH inducer clofibrate suppressed the growth of MC38 tumors and greatly alleviated tumor immunosuppression (Figure S7, Supporting Information). To verify whether CYP4X1^Hi^sEH^Lo^ contributes to colon cancer immune evasion through 14,15‐EET‐EA accumulation, we performed a 14,15‐EET‐EA supplementation experiment. As shown in **Figure** **3**A–C, exogenous replenishment of 14,15‐EET‐EA reversed *Cyp4x1* ^KD^ *Ephx2* ^OE^‐mediated tumor growth arrest and elevated the 14,15‐EET‐EA level in tumor tissues. 14,15‐EET‐EA compromised the effects of *Cyp4x1* ^KD^ *Ephx2* ^OE^ on the Treg infiltration, CD8^+^ T cell effector function, CAF activation, and vascular normalization (Figure 3D–G). To study whether CYP4X1^Hi^sEH^Lo^ mediates colon cancer immunosuppression via CD8^+^ T cells and Tregs, we performed the depletion experiments of CD8^+^ T cells and Tregs. Notably, the depletion of CD8^+^ T cells with anti‐CD8 antibody restored the growth of *Cyp4x1* ^KD^ *Ephx2* ^OE^ tumors (Figure S8A,B, Supporting Information). Conversely, the exhaustion of Tregs with anti‐CD25 antibody failed to further strengthen *Cyp4x1* ^KD^ *Ephx2* ^OE^‐induced tumor growth inhibition, CD8^+^ T cell infiltration, and effector function (Figure S8C–F, Supporting Information). These observations illustrate that CYP4X1/sEH‐derived 14,15‐EET‐EA orchestrates an immunosuppressive microenvironment, characterized by increased CAFs and Tregs, as well as reduced CD8^+^ T cells.

### 2.4 CYP4X1/sEH‐derived 14,15‐EET‐EA Mediates Tumor Immunosuppression via PD‐L1, CXCL12, and TGF‐β in CAFs

**Figure 4:**
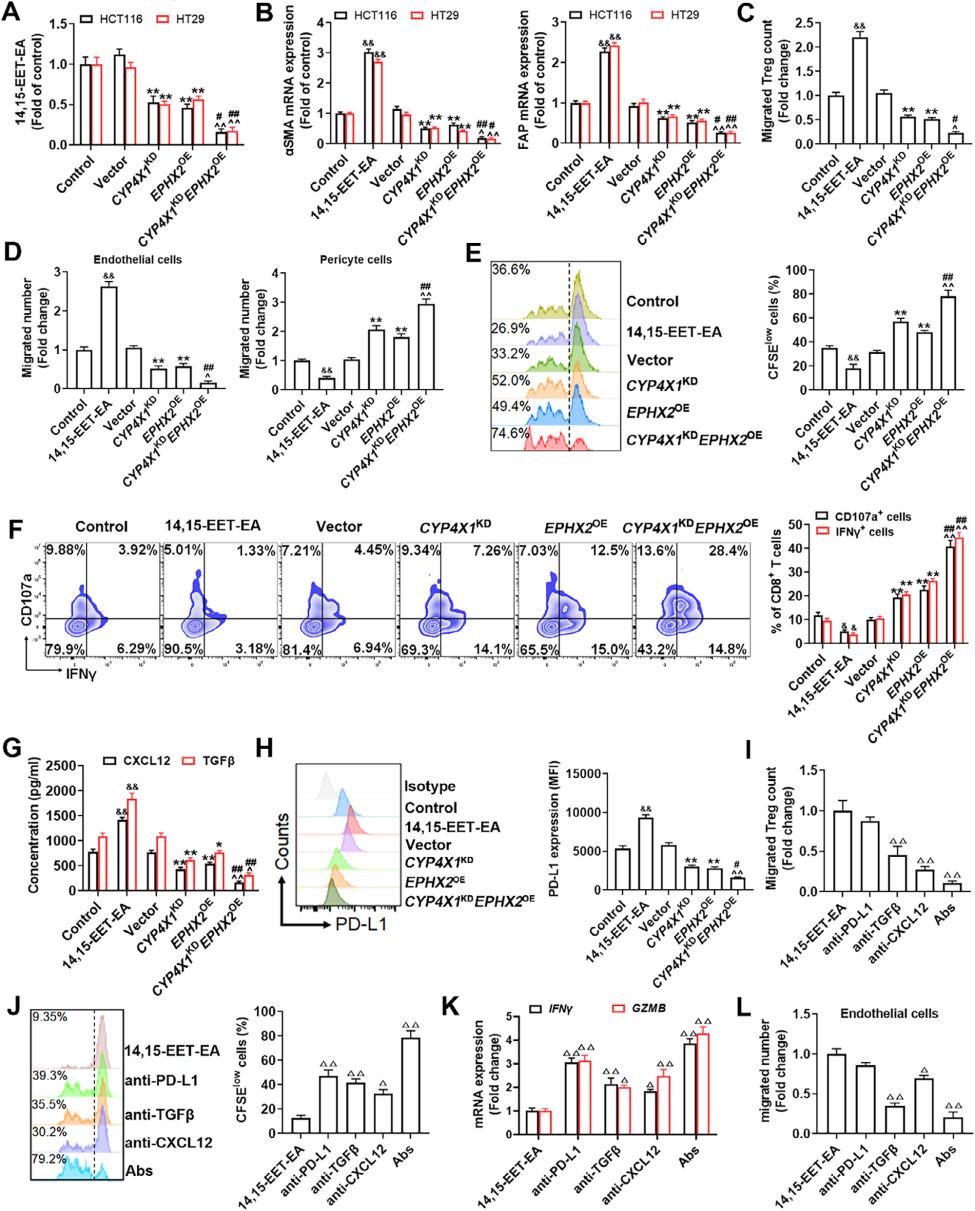
CYP4X1/sEH‐derived 14,15‐EET‐EA mediates tumor immunosuppression via upregulation of PD‐L1, CXCL12, and TGF‐β in CAFs. A) The production of 14,15‐EET‐EA in HCT116 and HT29 colon cancer cells expressing *CYP4X1* ^KD^, *EPHX2* ^OE^, or their combination (*CYP4X1* ^KD^ *EPHX2* ^OE^) was measured by LC‐MS/MS (*n* = 5). MRC5 fibroblasts were incubated with 14,15‐EET‐EA or conditioned medium (CM) from HCT116 or HT29 cells of the indicated groups (*n* = 5). B) αSMA and FAP mRNA expression levels in MRC5 fibroblasts incubated with 14,15‐EET‐EA or CM from HCT116 or HT29 cells of the indicated groups were measured by qPCR. C) Transwell migration analysis of Tregs cocultured with the above‐treated MRC5 fibroblasts. D) Transwell migration analysis of endothelial cells (HUVECs) and pericyte cells (human brain vascular pericytes) cocultured with the CM from above‐treated MRC5 fibroblasts. E) Carboxyfluorescein succinimidyl ester (CFSE) dilution was used to assess the proliferation of CD8^+^ T cells cocultured with the above‐treated MRC5 fibroblasts. F) The percentages of CD107a^+^ CD8^+^ T cells and IFN‐γ^+^ CD8^+^ T cells were determined by flow cytometry. G) CXCL12 and TGF‐β production in MRC5 fibroblasts of the indicated groups was measured by ELISA. H) PD‐L1 expression level in CAFs was determined by flow cytometry. MRC5 fibroblasts pretreated with 14,15‐EET‐EA were incubated with the treatment of alone or a combination (Abs) of neutralizing anti‐PD‐L1, anti‐CXCL12, and anti‐TGF‐β antibodies (*n* = 3). I) Transwell migration analysis of Tregs cocultured with the above‐treated MRC5 fibroblasts. J) CFSE dilution was used to measure the proliferation of CD8^+^ T cells cocultured with the above‐treated MRC5 fibroblasts. K) GZMB and IFN‐γ mRNA levels in CD8^+^ T cells were determined by qPCR. L) Transwell migration analysis of endothelial cells cocultured with the CM from the above‐treated MRC5 fibroblasts. Data are presented as mean ± SEM. *P* values were determined using one‐way ANOVA. ^&^ *P* < 0.05 and ^&&^ *P* < 0.01 vs. control; ^*^ *P* < 0.05 and ^**^ *P* < 0.01 vs. vector; ^^^ *P* < 0.05 and ^^^^ *P* < 0.01 vs. *CYP4X1* ^KD^; ^#^ *P* < 0.05 and ^##^ *P* < 0.01 vs. *EPHX2* ^OE^; ^Δ^ *P* < 0.05 and ^ΔΔ^ *P* < 0.01 vs. 14,15‐EET‐EA group.

Next, functional experiments were conducted to examine whether CYP4X1/sEH directly affects tumor cells or TME. We observed that *CYP4X1* ^KD^, *EPHX2* ^OE^ (Figure S9A, Supporting Information), and their combination (*CYP4X1* ^KD^ *EPHX2* ^OE^) had no significant effect on in vitro tumor cell proliferation, accompanied by an apparent reduction in 14,15‐EET‐EA production (**Figure** **4**A; Figure S9B, Supporting Information). Next, human MRC5 fibroblasts were treated with 14,15‐EET‐EA or conditioned medium (CM) from HCT116 or HT29 colon cancer cells expressing *CYP4X1* ^KD^, *EPHX2* ^OE^, or *CYP4X1* ^KD^ *EPHX2* ^OE^. We found that 14,15‐EET‐EA treatment potently upregulated αSMA and FAP in MRC5 fibroblasts, which were greatly diminished by *CYP4X1* ^KD^ or *EPHX2* ^OE^ CM (Figure 4B). MRC5 fibroblasts treated with *CYP4X1* ^KD^ or *EPHX2* ^OE^ CM decreased the migration of Tregs and endothelial cells, increased the migration of pericytes, and augmented the proliferation of CD8^+^ T cells and their expression of CD107a and IFNγ, which were restrained by 14,15‐EET‐EA (Figure 4C–F). Notably, the combination of *CYP4X1* ^KD^ and *EPHX2* ^OE^ was more efficient in inhibiting CAF activation, Treg and endothelial cell migration, promoting pericyte migration, and enhancing CD8^+^ T cell proliferation and effector function than *CYP4X1* ^KD^ or *EPHX2* ^OE^ alone (Figure 4B–F). These data imply that CYP4X1/sEH‐derived 14,15‐EET‐EA promotes CAF‐mediated immune escape in colon cancer.

To confirm whether the CYP4X1/sEH‐14,15‐EET‐EA system mediates immunosuppression via CAFs, MC38 cells with or without *Cyp4x1* ^KD^ *Ephx2* ^OE^ expression were mixed with GFP^+^ L929 fibroblasts and subcutaneously injected into the left flank of C57BL/6 mice. We observed that the co‐injection of *Cyp4x1* ^KD^ *Ephx2* ^OE^ MC38 cells with L929 fibroblasts significantly induced tumor growth arrest and CAF inactivation, along with an obvious decrease in intratumoral 14,15‐EET‐EA accumulation (Figure S10A–C, Supporting Information). Importantly, the co‐injection alleviated tumor immunosuppression, as evidenced by the reduced amount of Tregs, the enhanced CD8^+^ T cell infiltration and effector function, and the improved tumor perfusion (Figure S10D–G, Supporting Information). Next, 14,15‐EET‐EA‐ or vehicle‐treated GFP^+^ L929 fibroblasts mixed with MC38 cells were subcutaneously injected into the left flank of C57BL/6 mice. We discovered that co‐injection of 14,15‐EET‐EA‐treated L929 fibroblasts with MC38 cells significantly fostered immunosuppression in colon cancer (Figure S10A,C–G, Supporting Information). To further support the results obtained in the co‐implantation experiment, an in vivo CAF function inhibition experiment was carried out by using tranilast (a suppressor of CAFs).^[^ ^26^ ^]^ Unsurprisingly, tranilast treatment obviously attenuated 14,15‐EET‐EA‐induced immune evasion (Figure S10H–N, Supporting Information). These findings indicate that CYP4X1/sEH‐derived 14,15‐EET‐EA contributes to immune escape via CAFs.

CAFs produce cytokines CXCL12 and TGF‐β and express immune checkpoints to promote cancer immune escape.^[^ ^27^ ^]^ We took the intersection of *EPHX2*‐ and *CYP4X1*‐correlated cytokines in human colon cancer, and obtained six CAF‐derived cytokines, including *CXCL12*, *TGFB1*, *ANGPTL4*, *CSF2*, *GREM1*, and *IL1B* (GSE71187 and GSE21510; Figure S11A–C, Supporting Information). CAF‐expressed immune checkpoint *CD274* (PD‐L1) was positively correlated with *CYP4X1* but negatively correlated with *EPHX2* gene level in human colon cancer (GSE128435 and GSE71187; Figure S11D–G, Supporting Information). Next, qPCR was used to verify the CAF‐derived factors regulated by CYP4X1/sEH‐derived 14,15‐EET‐EA. We found that *CXCL12*, *TGFB1*, and *CD274*, rather than *ANGPTL4*, *CSF2*, *GREM1*, or *IL1B*, were downregulated by the CM from *CYP4X1* ^KD^ or *EPHX2* ^OE^ HCT116 cells, but upregulated by 14,15‐EET‐EA in human MRC5 fibroblasts (Figure S11H, Supporting Information). Especially, the combination of *CYP4X1* ^KD^ and *EPHX2* ^OE^ exerted more potent inhibitory effects on *CXCL12*, *TGFB1*, and *CD274* than either single treatment (Figure S11H, Supporting Information). Similar results were observed in CXCL12 and TGF‐β production and PD‐L1 protein expression in MRC5 fibroblasts (Figure 4G,H). Neutralizing antibodies against PD‐L1, CXCL12, or TGF‐β partially counteracted the effects of 14,15‐EET‐EA on the proliferation and effector function of CD8^+^ T cells, as well as the migration of Tregs and endothelial cells (Figure 4I–L). Remarkably, the combined blockade of PD‐L1, CXCL12, and TGF‐β achieved better efficacy than single blockade in abolishing the effects of 14,15‐EET‐EA (Figure 4I–L). These data reveal that CYP4X1/sEH‐derived 14,15‐EET‐EA promotes immunosuppression in colon cancer through the upregulation of PD‐L1, CXCL12, and TGF‐β in CAFs.

### 2.5 CYP4X1/sEH‐Derived 14,15‐EET‐EA Mediates Immunosuppression via GPR119 Activation in CAFs

**Figure 5:**
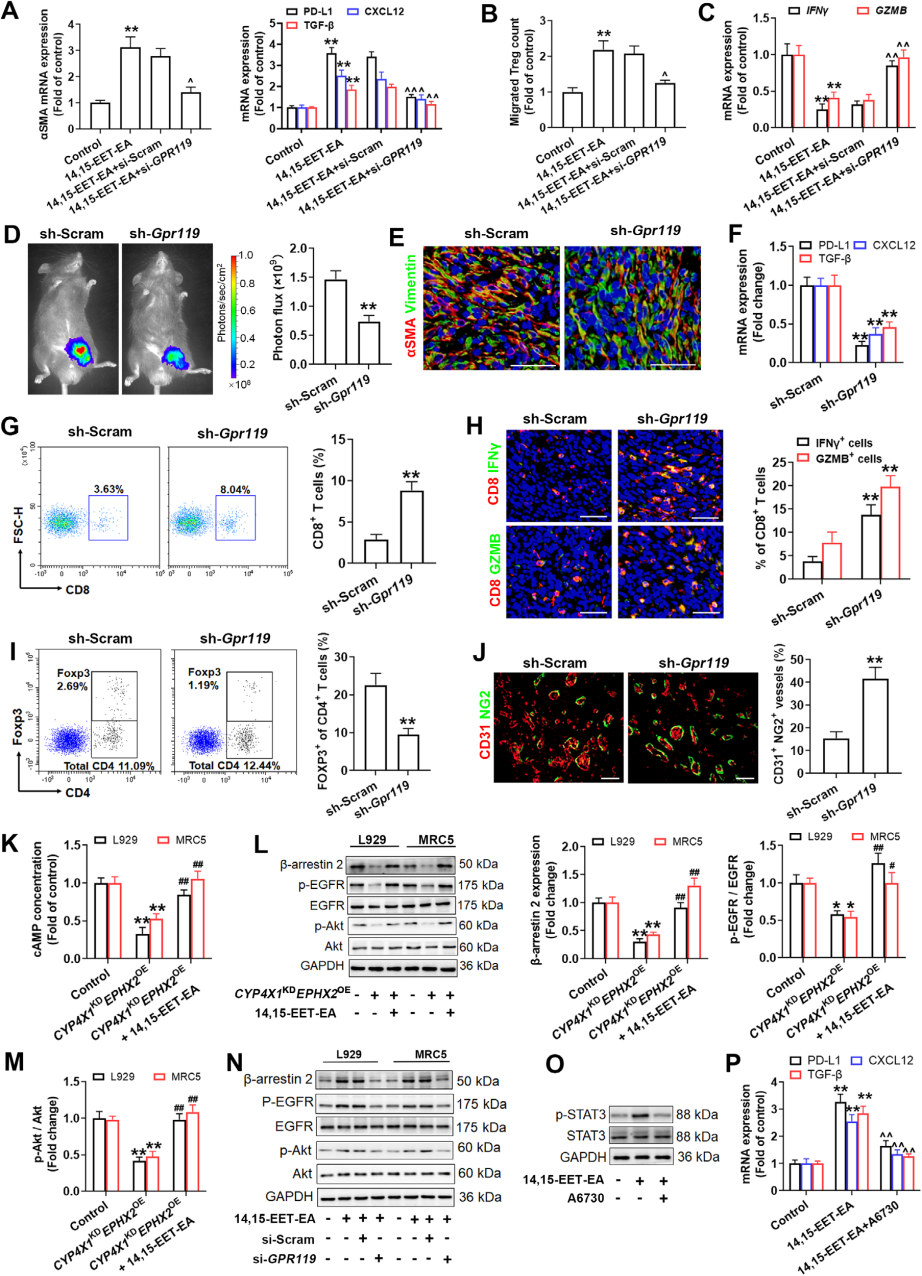
CYP4X1/sEH‐derived 14,15‐EET‐EA triggers immunosuppression via GPR119‐Gs/β‐arrestin 2 signaling. MRC5 fibroblasts were treated with or without GPR119 knockdown, followed by stimulation with 14,15‐EET‐EA. A) αSMA, PD‐L1, CXCL12, and TGF‐β mRNA levels in the above‐treated MRC5 fibroblasts (*n* = 5). B) Transwell migration analysis of Tregs cocultured with the above‐treated MRC5 fibroblasts (*n* = 5). C) GZMB and IFNγ mRNA levels in CD8^+^ T cells cocultured with the above‐treated MRC5 fibroblasts were measured by qPCR (*n* = 5). D) MC38‐luciferase cells were mixed with *Gpr119* shRNA‐ or scramble shRNA‐treated L929 fibroblasts, and then co‐implanted into C57BL/6 mice. In vivo bioluminescent images and quantification of the indicated groups (*n* = 8). E) Representative IF staining of αSMA (red) and vimentin (green) in MC38 tumor tissues (*n* = 5). Scale bar, 50 µm. F) PD‐L1, CXCL12, and TGF‐β mRNA levels in the CAFs (*n* = 5). G) The infiltration of CD8^+^ T cells in tumor tissues was determined by flow cytometry (*n* = 5). H) Representative IF staining and frequency analysis of CD8 (red) and GZMB (green) or IFN‐γ (green) in tumor tissues (*n* = 5). Scale bar, 50 µm. I) Treg accumulation in tumor tissues was analyzed and quantified by flow cytometry (*n* = 5). J) IF analysis of tumor vessel normalization in the indicated groups (*n* = 5). Scale bar, 50 µm. K) cAMP level in MRC5 and L929 fibroblasts incubated with the CM from *CYP4X1* ^KD^ *EPHX2* ^OE^‐expressed colon cancer cells with or without 14,15‐EET‐EA supplementation (*n* = 5). L,M) β‐arrestin 2, p‐EGFR, and p‐Akt protein levels in MRC5 and L929 fibroblasts incubated with CM from *CYP4X1* ^KD^ *EPHX2* ^OE^‐expressed colon cancer cells with or without 14,15‐EET‐EA supplementation (*n* = 5). N) β‐arrestin 2, p‐EGFR, and p‐Akt protein levels in L929 and MRC5 fibroblasts with or without *GPR119* knockdown, followed by stimulation with 14,15‐EET‐EA. O,P) p‐STAT3 protein level, PD‐L1, CXCL12, and TGF‐β mRNA levels in MRC5 fibroblasts of the indicated groups (*n* = 5). Data are presented as mean ± SEM. *P* values were determined using one‐way ANOVA (A‐C, K‐M, and P) or Student's *t*‐tests (D, F‐J). ^*^ *P* < 0.05 and ^**^ *P* < 0.01 vs. control or sh‐Scram; ^^^ *P* < 0.05 and ^^^^ *P* < 0.01 vs. 14,15‐EET‐EA+si‐Scram or 14,15‐EET‐EA; ^#^ *P* < 0.05 and ^##^ *P* < 0.01 vs. *CYP4X1* ^KD^ *EPHX2* ^OE^ group.

To explore the biological functions of the differentially expressed genes (DEGs) between *CYP4X1* ^Hi^ *EPHX2* ^Lo^ and *CYP4X1* ^Lo^ *EPHX2* ^Hi^ groups, we performed Gene Ontology (GO) enrichment analysis in the TCGA‐COAD dataset, and found that these DEGs were enriched in G protein‐coupled receptor (GPCR) binding (Figure S12A, Supporting Information). We subsequently overlapped *CYP4X1*‐related GPCRs (GSE33193 and GSE128435) with *EPHX2*‐related GPCRs (GSE33193), and identified *GPR119* and *GRM4* (Figure S12B, Supporting Information). Moreover, *GPR119* exhibited a stronger correlation with CAF‐derived *CD274*, *CXCL12*, and *TGFB1* than *GRM4* did in human colon cancer (GSE75500; Figure S12C, Supporting Information). Importantly, the *CYP4X1* ^KD^ *EPHX2* ^OE^ CM downregulated GPR119 but not GRM4 in CAFs, and 14,15‐EET‐EA supplementation remarkably reversed *CYP4X1* ^KD^ *EPHX2* ^OE^‐mediated GPR119 downregulation (Figure S12D, Supporting Information). Bioinformatic analyses revealed that *GPR119* was upregulated in human colon cancer tissues compared with normal tissues (GSE71187 and GSE128435; Figure S12E,F, Supporting Information). The IHC staining confirmed that the GPR119 protein level was higher in human colon cancer tissues than in adjacent normal tissues (Figure S12G, Supporting Information). Hence, GPR119 was selected for further research. Molecular docking analysis demonstrated that 14,15‐EET‐EA effectively bound to the active pocket of GPR119 (Figure S13A, Supporting Information). Specifically, the ligand formed hydrogen bonds with the receptor residues E261 and S156 (Figure S13B, Supporting Information). Besides, several hydrophobic interactions were formed by the skeleton of 14,15‐EET‐EA with the receptor residues, including W238, F241, F157, V93, and A89 (Figure S13B, Supporting Information). The cellular thermal shift assay demonstrated that 14,15‐EET‐EA induced thermal stabilization of the GPR119 protein in MC38 CM‐treated L929 fibroblasts (Figure S13C,D, Supporting Information). To determine whether GPR119 in CAFs contributes to 14,15‐EET‐EA‐mediated immunosuppression, we performed GPR119 knockdown in an in vitro co‐cultured model comprising CAFs and immune cells. As shown in **Figure** **5**A–C, *GPR119* siRNA markedly restrained the activation of CAFs and their expression of PD‐L1, CXCL12, and TGF‐β, and counteracted the increased Treg migration and CD8^+^ T cell anergy in response to 14,15‐EET‐EA stimulation. To study whether GPR119 deletion in CAFs phenocopies *Cyp4x1* ^KD^ *Ephx2* ^OE^‐mediated anti‐tumor immune response in vivo, sh*Gpr119*‐treated L929 cells were co‐injected with MC38 cells to establish a subcutaneous colon cancer model. We found that *Gpr119* knockdown in CAFs induced MC38 tumor growth arrest and greatly attenuated CAF‐mediated immunosuppression (Figure 5D–J). These findings indicate that GPR119 in CAFs is responsible for immune escape triggered by CYP4X1/sEH‐derived 14,15‐EET‐EA in colon cancer.

### 2.6 CYP4X1/sEH‐Derived 14,15‐EET‐EA Mediates Immunosuppression Partially via GPR119‐Gs‐cAMP Pathway

GPR119 is known to signal through multiple G proteins (Gs, Gq, and Gi) as well as β‐arrestins.^[^ ^28^ ^]^ We conducted GO enrichment analysis of the DEGs between the *GPR119* high expression (*GPR119* ^Hi^) and *GPR119* low expression (*GPR119* ^Lo^) groups, and found that these DEGs were enriched in the G protein‐coupled receptor signaling pathway coupled to cyclic nucleotide second messenger (Figure S14A, Supporting Information). Moreover, the levels of *CYP4X1*, *EPHX2*, and *GPR119* were correlated with *ARRB2* (encoding β‐arrestin 2) but not with *ARRB1* (encoding β‐arrestin 1) in human colon cancer (Figure S14B–D, Supporting Information). Next, we combined genetic and pharmacological approaches to investigate the potential roles of different G protein and arrestin subtypes. Intriguingly, 14,15‐EET‐EA‐mediated activation of CAFs was weakened by the Gs inhibitor (NF449) and β‐arrestin 2 siRNA, rather than by the Gq inhibitor (YM‐254890) or the Gi inhibitor (pertussis toxin, PTX) (Figure S14E, Supporting Information). *CYP4X1* ^KD^ *EPHX2* ^OE^ CM notably inhibited cAMP accumulation, which was restored by supplementing with 14,15‐EET‐EA (Figure 5K). *GPR119* siRNA markedly dampened the cAMP production in response to 14,15‐EET‐EA stimulation in MRC5 fibroblasts (Figure S14F, Supporting Information). Next, the concentration‐dependent effect of 14,15‐EET‐EA on GPR119 was examined in a heterologous expression system. OEA, the most potent endogenous agonist of GPR119, was used as the positive control.^[^ ^29^ ^]^ Similar to a previous report,^[^ ^21^ ^]^ 14,15‐EET‐EA induced a concentration‐dependent cAMP accumulation (EC_50_ = 3.74 µM) and exhibited a lower maximal response than OEA (Figure S14G, Supporting Information). Alanine mutations of E261 and W238 significantly attenuated 14,15‐EET‐EA‐induced GPR119 activation (Figure S14H, Supporting Information). To map the downstream signaling cascade of 14,15‐EET‐EA‐GPR119, we performed KEGG enrichment analysis for DEGs between the *GPR119* ^Hi^ and *GPR119* ^Lo^ groups, and observed significant enrichment in the PI3K/Akt pathway (Figure S14I, Supporting Information). Accordingly, 14,15‐EET‐EA supplementation restored the *CYP4X1* ^KD^ *EPHX2* ^OE^ CM‐mediated Akt inactivation (Figure 5L,M). *GPR119* siRNA markedly suppressed 14,15‐EET‐EA‐mediated Akt activation in MRC5 fibroblasts (Figure 5N). Forskolin, a direct adenylyl cyclase‐cAMP activator, partially weakened the *GPR119* siRNA‐induced reduction in PD‐L1, CXCL12, TGF‐β, and p‐Akt levels in MRC5 fibroblasts exposed to 14,15‐EET‐EA (Figure S14J,K, Supporting Information). These data suggest that CYP4X1/sEH‐derived 14,15‐EET‐EA mediates immunosuppression partially via the GPR119‐Gs‐cAMP pathway.

### 2.7 CYP4X1/sEH‐Derived 14,15‐EET‐EA Induces Immunosuppression Partially via GPR119‐Mediated EGFR Transactivation

Activation of the Gs‐cAMP pathway only partially reversed the downregulation of CAF‐derived immunosuppressive factors elicited by GPR119 knockdown, indicating the involvement of additional signaling cascades. GPCRs induce transactivation of the epidermal growth factor receptor (EGFR) by recruiting β‐arrestins.^[^ ^30^ ^]^ GO enrichment analysis revealed that GPR119‐regulated genes were enriched in EGFR signaling in human colon cancer (Figure S14A, Supporting Information). The scRNA‐seq analysis manifested that the *EGFR* gene was highly enriched in the fibroblast cluster (Figure S15A, Supporting Information). The PathHunter β‐arrestin recruitment assay substantiated that GPR119 dose‐dependently recruited β‐arrestin 2 in response to 14,15‐EET‐EA stimulation (EC_50_ = 3.0 µM; Figure S15B, Supporting Information). Notably, *CYP4X1* ^KD^ *EPHX2* ^OE^ CM downregulated β‐arrestin 2 and p‐EGFR in CAFs, which were reversed by 14,15‐EET‐EA (Figure 5L). *GPR119* siRNA attenuated the augmentation of β‐arrestin 2 and the p‐EGFR in L929 and MRC5 fibroblasts upon 14,15‐EET‐EA stimulation (Figure 5N). Both β‐arrestin 2 siRNA and EGFR inhibitor impaired the effects of 14,15‐EET‐EA on p‐EGFR or p‐AKT (Figure S15C,D, Supporting Information). The PI3K/AKT signaling pathway promotes PD‐L1 and CXCL12 transcription by increasing the p‐STAT3 content.^[^ ^31^, ^32^ ^]^ We performed promoter prediction using the JASPAR database and identified *TGFB1* as a STAT3 target gene (Figure S16A, Supporting Information). As expected, the Akt inhibitor A6730 reversed 14,15‐EET‐EA‐induced STAT3 activation, leading to an obvious reduction in PD‐L1, CXCL12, and TGF‐β levels (Figure 5O,P). Colivelin, a STAT3 activator,^[^ ^33^ ^]^ counteracted the inhibitory effects of A6730 on PD‐L1, CXCL12, and TGF‐β levels in response to 14,15‐EET‐EA stimulation (Figure S16B, Supporting Information). These results indicate that CYP4X1/sEH‐derived 14,15‐EET‐EA drives immunosuppression through GPR119‐mediated EGFR transactivation in addition to classical activation.

### 2.8 The CYP4X1/sEH‐14,15‐EET‐EA‐GPR119 Axis Regulates Sensitivity to Anti‐PD‐1 Therapy

**Figure 6:**
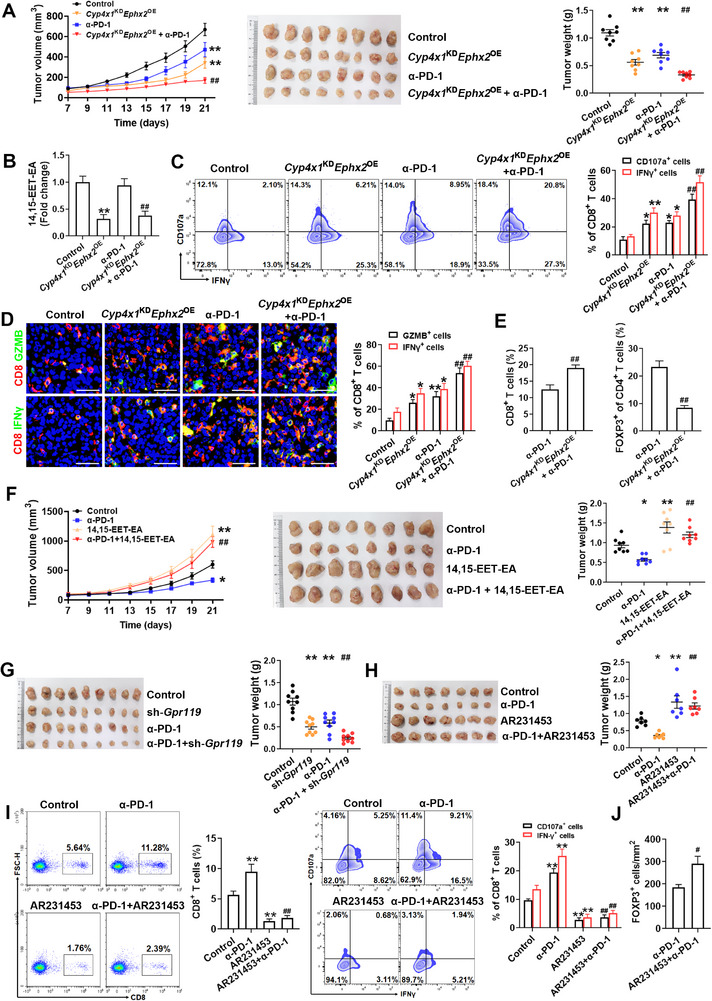
The CYP4X1/sEH‐14,15‐EET‐EA‐GPR119 axis regulates sensitivity to anti‐PD‐1 therapy. A) MC38 colon cancer cells with or without *Cyp4x1* ^KD^ *Ephx2* ^OE^ expression were subcutaneously implanted into C57BL/6 mice and treated with IgG or anti‐PD‐1 antibody (α‐PD‐1). Tumor growth curves (Two‐way ANOVA) and tumor weights were presented (*n* = 8). B) 14,15‐EET‐EA level was determined by LC‐MS/MS (*n* = 6). C) Representative flow staining and quantification of CD107a^+^ CD8^+^ T cells and IFN‐γ^+^ CD8^+^ T cells in tumor tissues of the indicated groups (*n* = 6). D) Representative IF staining and quantification of CD8 (red) and GZMB (green) or IFN‐γ (green) in tumor tissues (*n* = 6). Scale bar, 50 µm. E) Percentages of CD8^+^ T cells and Tregs (*n* = 6). F) Tumor growth curves (Two‐way ANOVA) and tumor burdens of MC38 tumor‐bearing mice treated with IgG control, anti‐PD‐1 alone, or in combination with 14,15‐EET‐EA (*n* = 8). G) C57BL/6 mice were co‐implanted with MC38 cells and *Gpr119* shRNA‐ or scramble shRNA‐treated L929 fibroblasts, followed by anti‐PD‐1 treatment. Tumor weights were measured in the indicated groups (*n* = 9). H) C57BL/6 mice inoculated with MC38 cells were treated with GPR119 agonist AR231453, anti‐PD‐1 alone, or their combination. Tumor weights were measured in the indicated groups (*n* = 7). I) Representative flow staining and quantification of CD8^+^ T cells, CD107a^+^ CD8^+^ T cells, and IFN‐γ^+^ CD8^+^ T cells in tumor tissues of the indicated groups (*n* = 5). J) Treg accumulation in tumors was analyzed and quantified by IHC staining (*n* = 5). Data are presented as mean ± SEM. *P* values were determined using one‐way ANOVA (A‐D, F‐I) and Student's *t*‐tests (E and J). ^*^ *P* < 0.05 and ^**^ *P* < 0.01 vs. control; ^#^ *P* < 0.05 and ^##^ *P* < 0.01 vs. α‐PD‐1 group.

To explore whether CYP4X1/sEH‐14,15‐EET‐EA‐GPR119 axis regulates sensitivity to anti‐PD‐1 therapy in colon cancer, MC38 tumor‐bearing C57BL/6 mice were treated with *Cyp4x1* ^KD^ *Ephx2* ^OE^ and an anti‐PD‐1 antibody (α‐PD‐1). Although both monotherapy arms exhibited partial tumor control, the combination of *Cyp4x1* ^KD^ *Ephx2* ^OE^ with α‐PD‐1 therapy significantly reduced tumor volumes and weights, accompanied by a pronounced reduction in 14,15‐EET‐EA level in the tumor tissues (**Figure** **6**A,B). Importantly, the combination treatment boosted the infiltration of CD8^+^ T cells and their expression of CD107a, IFNγ, and GZMB beyond α‐PD‐1 alone, along with an apparent decline in Treg frequency in the MC38 tumors (Figure 6C–E). Similar results were obtained in the CT26 colon cancer model (Figure S17, Supporting Information). In another experiment, we observed that exogenous supplementation of 14,15‐EET‐EA impaired the efficacy of anti‐PD‐1 therapy (Figure 6F). G protein‐coupled receptors (GPCRs) currently serve as molecular targets of more than one‐third of all approved therapeutic agents, underscoring the potential of GPR119 as a druggable target to enhance the efficacy of anti‐PD‐1 immunotherapy.^[^ ^34^ ^]^ We treated C57BL/6 mice bearing orthotopic MC38 tumors with si‐*Gpr119* plus α‐PD‐1 (α‐PD‐1 monotherapy as a control), and verified that *Gpr119* knockdown by specific siRNA potentiated the response to anti‐PD‐1 therapy (Figure S18A, Supporting Information). Next, we evaluated whether the pharmacological inhibition of GPR119 by arvanil^[^ ^21^ ^]^ is effective in sensitizing colon cancer to anti‐PD‐1 therapy. The combination of arvanil with anti‐PD‐1 treatment significantly inhibited tumor growth and Treg enrichment, and promoted the infiltration of CD8^+^ T cells and their expression of IFNγ and GZMB in colon cancer without apparent additional toxicity (Figure S18B–E and Table S1, Supporting Information). Given that arvanil may act on non‐GPR119 receptors,^[^ ^35^ ^]^ we utilized a co‐injection model of MC38 cells and sh*Gpr119*‐transduced L929 cells to evaluate the role of GPR119 in anti‐PD‐1 therapy. As shown in Figure 6G, the combination of sh*Gpr119* with α‐PD‐1 therapy achieved a stronger growth inhibitory effect against MC38 tumors. In stark contrast to anti‐PD‐1 monotherapy, treatment with the GPR119 agonist AR231453 induced immunosuppression and conferred resistance to anti‐PD‐1 therapy in colon cancer (Figure 6H–J). These findings demonstrate that the CYP4X1/sEH‐14,15‐EET‐EA‐GPR119 axis regulates sensitivity to anti‐PD‐1 therapy in colon cancer.

### 2.9 CYP4X1/sEH‐GPR119 Axis Predicts Response to Anti‐PD‐1 Therapy in Human Colon Cancer

**Figure 7:**
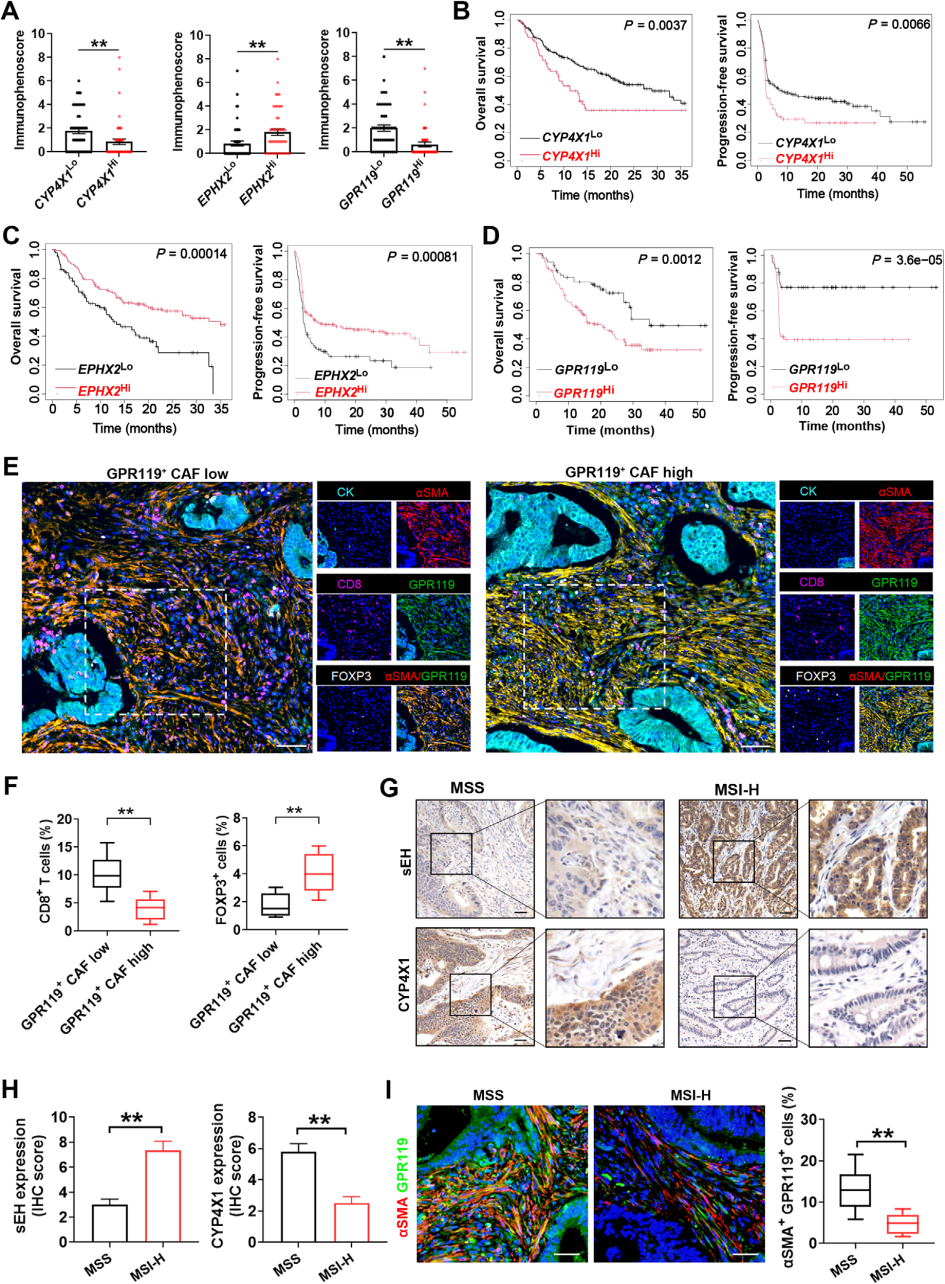
CYP4X1/sEH‐GPR119 axis predicts the response to anti‐PD‐1 therapy in colon cancer. A) Immunophenoscore in the *CYP4X1* ^Hi^ vs. *CYP4X1* ^Lo^ groups, *EPHX2* ^Hi^ vs. *EPHX2* ^Lo^ groups, and the *GPR119* ^Hi^ and *GPR119* ^Lo^ groups (GSE33193). B‐D) Kaplan‐Meier plots of overall survival and progression‐free survival for patients receiving PD‐1 blockade therapy according to *CYP4X1*, *EPHX2*, or *GPR119* expression. E) Representative images of mIF staining in human colon cancer tissues with low GPR119^+^ CAF or high GPR119^+^ CAF abundance. Scale bar, 50 µm. F) Quantification of CD8^+^ T cells and FOXP3^+^ Tregs as a proportion of total cells (*n* = 6). G,H) Representative images of IHC staining and quantification of CYP4X1 and sEH in microsatellite stability (MSS; *n* = 15) and microsatellite instability‐high (MSI‐H; *n* = 6) human colon cancer tissues. Scale bar, 50 µm. I) Representative IF staining and quantification of αSMA (red) and GPR119 (green) in MSS (*n* = 15) and MSI‐H (*n* = 6) human colon cancer tissues. Scale bar, 50 µm. Data are shown as mean ± SEM. *P* values were determined using Mann‐Whitney tests (A), log‐rank tests (B‐D), or Student's *t*‐tests (F, H, and I). ^**^ *P* < 0.01.

The immunophenoscore is known to determine tumor immunogenicity and predict response to immunotherapy in multiple tumor types.^[^ ^36^ ^]^ Our analysis revealed that colon cancer patients with *CYP4X1* ^Lo^, *EPHX2* ^Hi^, or *GPR119* ^Lo^ expression exhibited elevated immunophenoscores compared to their respective counterparts (GSE33193; **Figure** **7**A), indicating that these patients might respond better to immunotherapy. Due to the lack of accessible datasets for colon cancer patients treated with immunotherapy, we analyzed publicly available datasets from other cancer patients treated with immune checkpoint blockade. Non‐responders to PD‐1 blockade exhibited higher *CYP4X1* and lower *EPHX2* expression than responders (Figure S19A, Supporting Information; https://rocplot.org/immune). In a pan‐cancer cohort of patients treated with PD‐1 blockade, both *CYP4X1* ^Lo^ and *EPHX2* ^Hi^ were associated with improved OS (*n* = 238) and PFS (*n* = 283) (Figure 7B,C; http://kmplot.com/analysis/). In the independent IMvigor210 cohort or the RCC‐Braun_2020 dataset, patients with *CYP4X1* ^Lo^ or *EPHX2* ^Hi^ experienced significantly prolonged OS under PD‐1 blockade therapy (Figure S19B,C, Supporting Information). Patients with *GPR119* ^Hi^ were closely associated with shorter OS and PFS in cancer patients receiving PD‐1 blockade therapy (Figure 7D; http://kmplot.com/analysis/). The *GPR119* gene level was positively correlated with Treg (GSE106582) and CAF (GSE31905) levels but negatively correlated with CD8^+^ T cell (GSE71187) infiltration (Figure S20, Supporting Information). The mIF staining verified a lower CD8^+^ T cell infiltration and a higher Treg proportion in patients with high GPR119^+^ CAF abundance in human colon cancer tissues (Figure 7E,F). CAF subtypes have been reported to serve as predictive biomarkers for immunotherapy response.^[^ ^37^ ^]^ Thus, the scRNA‐seq dataset (GSE132465) was employed to classify the subpopulations of CAFs based on their gene expression profiles: myofibroblastic CAFs (myCAFs), inflammatory CAFs (iCAFs), tumor‐like CAFs (tCAFs), dividing CAFs (dCAFs), and antigen‐presenting CAFs (apCAFs) (Figure S21A, Supporting Information). We found that CAF subtypes exhibited uneven abundances between the *CYP4X1* ^Hi^ *EPHX2* ^Lo^ and *CYP4X1* ^Lo^ *EPHX2* ^Hi^ groups. Specifically, myCAFs and iCAFs tended to increase in the *CYP4X1* ^Hi^ *EPHX2* ^Lo^ group than in the *CYP4X1* ^Lo^ *EPHX2* ^Hi^ group, while the opposite distribution pattern was observed for apCAFs, dCAFs, and tCAFs (Figure S21B, Supporting Information). The mIF staining enables the simultaneous detection of multiple markers on a single tissue section, enabling comprehensive analysis of cell composition and function.^[^ ^38^ ^]^ Next, mIF staining was used to verify CAF subtypes known to regulate tumor immunity. We observed that the proportions of myCAFs (PDPN^+^ αSMA^+^) and iCAFs (PDPN^+^ CXCL12^+^) were significantly lower in human colon cancer tissues with CYP4X1^Lo^sEH^Hi^ compared to those with CYP4X1^Hi^sEH^Lo^. In contrast, the proportion of apCAFs (PDPN^+^ HLA‐DRA^+^) did not differ between the CYP4X1^Lo^sEH^Hi^ and CYP4X1^Hi^sEH^Lo^ groups (Figure S21C,D, Supporting Information). We also observed notable colocalization of GPR119 with the myCAF marker αSMA and the iCAF marker CXCL12, but not with the apCAF marker HLA‐DRA, indicating that GPR119⁺ fibroblasts may belong to the myCAF and iCAF subpopulations rather than apCAFs (Figure S21C, Supporting Information). Patients with microsatellite instability‐high (MSI‐H) exhibit greater sensitivity to immunotherapy compared to those with microsatellite stability (MSS).^[^ ^39^ ^]^ Therefore, we assessed the correlations of CYP4X1 and sEH protein levels with tumor MSI status, and observed higher CYP4X1 levels and lower sEH levels in human colon cancer with MSS compared to those with MSI‐H (Figure 7G,H). Consistently, a significant increase in GPR119^+^ CAF infiltration was observed in MSS colon cancer tissues relative to MSI‐H tissues (Figure 7I). These data indicate that the CYP4X1/sEH‐GPR119 axis is a predictive biomarker for anti‐PD‐1 therapy in colon cancer.

## 3 Discussion

Our previous study validated that CYP4X1‐catalyzed endocannabinoid metabolism is implicated in tumor growth and angiogenesis.^[^ ^20^ ^]^ Here, we provided the first direct evidence that CYP4X1/sEH‐dependent endocannabinoid metabolism drives CAF‐mediated tumor immunosuppression and immunotherapy resistance in colon cancer. To our knowledge, this is the first study that directly demonstrated the key role of CYP4X1/sEH‐14,15‐EET‐EA system in tumor immune evasion. In addition, we uncovered for the first time that CYP4X1/sEH‐derived 14,15‐EET‐EA triggers tumor immunosuppression via GPR119‐Gs/β‐arrestin 2 signaling. Importantly, targeting the CYP4X1/sEH‐14,15‐EET‐EA‐GPR119 axis may provide a novel therapeutic strategy for sensitizing anti‐PD‐1 therapy in human colon cancer.

The ECS maintains immune homeostasis and functions as a gatekeeper of the immune system.^[^ ^40^ ^]^ The ECS, including AEA and its receptor CB_2_, shapes the tumor immune microenvironment.^[^ ^40^ ^]^ However, the complete role of the ECS in cancer immune evasion remains unknown. Here, we observed an upregulation of *CYP4X1* and a downregulation of *EPHX2* in human colon cancer tissues, similar to previous reports.^[^ ^17^, ^41^ ^]^ Moreover, CYP4X1 knockdown significantly reduced 14,15‐EET‐EA production in tumor tissues without significantly elevating its precursor AEA. When a specific lipid metabolic pathway is blocked, metabolic flux is rapidly rerouted to alternative pathways to maintain balance.^[^ ^42^, ^43^ ^]^ A previous study demonstrated that AEA is metabolized by cyclooxygenase‐2 (COX‐2) in addition to CYP4X1.^[^ ^9^ ^]^ Thus, CYP4X1 knockdown led to a reduction in 14,15‐EET‐EA without a significant increase in AEA, presumably because of compensatory metabolism of the excess AEA by COX‐2. Additionally, we demonstrated that CYP4X1 inhibition and sEH overexpression synergistically shaped an anti‐tumor microenvironment, thereby sensitizing colon cancer to anti‐PD‐1 therapy. In comparison, pharmacologic sEH inhibition enhances cancer immunotherapy.^[^ ^44^ ^]^ The divergence may be due to differences in the intervention strategies (*CYP4X1* inhibition plus *EPHX2* overexpression vs. pharmacologic sEH inhibition) and the tumor models (colon cancer vs. bladder, prostate cancer, and melanoma), as well as differences in the substrates of sEH (14,15‐EET‐EA vs. epoxy‐fatty acids). Several studies have shown that gut microbiota, including *Fusobacterium nucleatum* (*F. nucleatum*), modulate the enzymes responsible for the synthesis and degradation of endocannabinoids.^[^ ^45^, ^46^ ^]^ Here, we observed that *F. nucleatum* provoked an increasing trend in *CYP4X1* expression and a repression in *EPHX2* expression (GSE141805), suggesting that *CYP4X1* and *EPHX2* may be regulated by *F. nucleatum*. Further experiments are being performed to investigate the possibility.

Few efforts have been made to elucidate the lipid metabolic interactions between CAFs and tumor cells.^[^ ^47^ ^]^ Our recent study manifested that the tumor metabolite 20‐HETE triggers the initial signaling events in the TME to alter the CAF phenotype.^[^ ^48^ ^]^ CAFs promote CD8^+^ T cell anergy and exhaustion, as well as Treg infiltration.^[^ ^37^ ^]^ Targeting the inhibition of CAFs alleviates immunosuppression and consequently sensitizes tumors to immunotherapy.^[^ ^49^ ^]^ However, the role of lipid metabolite‐mediated tumor‐fibroblast crosstalk in immune escape and immunotherapy resistance has only begun to be appreciated. Here, we identified a previously unknown tumor‐stromal crosstalk mediated by 14,15‐EET‐EA (an endocannabinoid‐like lipid molecule) as an essential regulator of immune escape and immunotherapy resistance in colon cancer. CAFs reshape the TME and cause immunosuppression through the production of immunosuppressive cytokines (e.g., TGF‐β, CXCL12) and the expression of coinhibitory molecule PD‐L1/2.^[^ ^49^ ^]^ Our data revealed that CYP4X1/sEH‐derived metabolite 14,15‐EET‐EA fostered the production of CAF‐derived cytokines (TGF‐β, CXCL12) and induced the expression of PD‐L1, thereby instigating the expansion of Tregs and the anergy and exhaustion of CD8^+^ T cells. Notably, combined blockade of PD‐L1, TGF‐β, and CXCL12 exerted better efficacy than single blockade in abolishing the immunosuppressive effects of 14,15‐EET‐EA. Considering that CYP4X1 and sEH dysregulation trigger immunosuppressive signaling via multiple CAF‐derived cytokines, novel targeted therapies based on CYP4X1/sEH‐dependent AEA metabolism, not any single cytokine, may provide a potential therapeutic strategy for disrupting tumor‐fibroblast crosstalk to sensitize checkpoint blockade therapy in human colon cancer.

GPCRs play a pivotal role in cancer immune evasion and may serve as novel targets for sensitizing cancer immunotherapy.^[^ ^50^ ^]^ GPR119 is implicated in the induction of Tregs in high‐fat diet‐induced obese mice.^[^ ^22^ ^]^ However, the role of GPR119 in the tumor immune microenvironment and immunotherapy resistance has not been reported. Here, we demonstrated that GPR119 inhibition reversed the immunosuppressive microenvironment, thereby enhancing the efficacy of anti‐PD‐1 therapy in colon cancer with no extra toxic effects. GPR119, a lipid sensor, has emerged as a promising therapeutic target for type 2 diabetes (T2D) and obesity.^[^ ^51^, ^52^ ^]^ The agonists of GPR119 have recently received wide attention as potential therapeutics for T2D and obesity.^[^ ^52^ ^]^ A previous study has shown that *Gpr119* ^−/−^ mice exhibit normal body weight and glucose tolerance on a regular chow diet.^[^ ^53^ ^]^ Further experiments are being conducted to elucidate the precise mechanisms by which GPR119 antagonists sensitize colon cancer to anti‐PD‐1 therapy without increasing the risk of developing hyperglycemia or obesity. Obesity has been linked to an increased risk of colon cancer.^[^ ^54^, ^55^ ^]^ Here, we observed that in both obese and non‐obese colon cancer patients, *CYP4X1* was upregulated and *EPHX2* was downregulated in tumor tissues compared to adjacent normal tissues (TCGA; Figure S22A,B, Supporting Information). Furthermore, *GPR119* expression was significantly higher in obese colon cancer patients than in non‐obese counterparts (TCGA; Figure S22C, Supporting Information). We therefore propose that, in both obese and non‐obese patients with colon cancer, CYP4X1^Hi^sEH^Lo^ leads to the accumulation of 14,15‐EET‐EA, thereby powerfully activating GPR119 to promote immunosuppression in colon cancer. Here, 14,15‐EET‐EA level was measured in four types of human colon cancer tissues (CYP4X1^Hi^sEH^Lo^, CYP4X1^Hi^sEH^Hi^, CYP4X1^Lo^sEH^Lo^, CYP4X1^Lo^sEH^Hi^). We observed that human colon cancer tissues with the CYP4X1^Hi^sEH^Lo^ signature exhibited the highest concentration of 14,15‐EET‐EA at ≈2.8 pmol mg^−1^ (corresponding to ≈2.8 µM, assuming a tissue density of 1 g mL^−1^) (Figure S23, Supporting Information), a level close to the EC_50_ for GPR119 activation. These data indicate that 14,15‐EET‐EA activates GPR119 to mediate immunosuppression in human colon cancer. Our previous study confirmed that 14,15‐EET‐EA upregulates PPARα.^[^ ^56^ ^]^ Here, we found that, apart from activating GPR119, 14,15‐EET‐EA increased GPR119 expression level. A positive correlation between *PPARA* and *GPR119* gene expression levels was also observed in the GSE75500 dataset (Figure S24, Supporting Information). Thus, we speculate that the upregulation of GPR119 may be attributed to the activation of PPARα by 14,15‐EET‐EA.

GPR119 primarily signals through coupling to multiple G protein subtypes (Gs, Gq, and Gi) as well as β‐arrestins.^[^ ^28^ ^]^ Our earlier study reported that 14,15‐EET‐EA induces EGFR activation, leading to tumor angiogenesis.^[^ ^20^ ^]^ The classical activation and transactivation modes of GPCR are associated with cancer immune escape and subsequent cancer progression.^[^ ^57^, ^58^, ^59^ ^]^ Here, our data indicated that 14,15‐EET‐EA mediated the formation of an immunosuppressive TME via both GPR119‐mediated classical activation and EGFR transactivation mechanisms, thereby targeting inhibition of both GPR119 and EGFR may yield better therapeutic efficacy in human colon cancer. Given the fact that the 14,15‐EET‐EA‐GPR119‐Gs‐PI3K/Akt axis may serve as a compensatory mechanism to maintain an immunosuppressive TME, our findings provide a potential explanation for why anti‐EGFR therapy exhibits resistance in a subset of colon cancer patients. Given that different CAF subtypes, including iCAFs and myCAFs, play crucial roles in promoting or restraining tumor progression,^[^ ^37^ ^]^ it is essential to identify specific markers of the tumor‐promoting CAF subsets and precisely target them. Our recent study identified GPR75^+^ FAP^+^ CAFs as a new protumor CAF subset in NSCLC.^[^ ^48^ ^]^ Herein, we demonstrated that *GPR119* ^Hi^ was closely associated with shorter OS and PFS in the cancer patients receiving PD‐1 blockade therapy and that GPR119 knockdown in CAFs notably suppressed intratumoral Treg enrichment and CD8^+^ T cell exhaustion. Obviously, the abundance of GPR119^+^ CAFs was negatively correlated with CD8^+^ T cell accumulation and positively correlated with Treg infiltration in human colon cancer. GPR119⁺ fibroblasts expressed the myCAF marker αSMA and the iCAF marker CXCL12, suggesting that GPR119⁺ fibroblasts may represent a heterogeneous population comprising both myCAFs and iCAFs. These data imply that GPR119 not only presents a promising therapeutic target in human colon cancer but also serves as a surface marker for protumor CAF subsets.

The current study still has limitations. We demonstrated that CYP4X1/sEH‐dependent endocannabinoid metabolism drives CAF‐mediated immunosuppression and immunotherapy resistance in colon cancer. The therapeutic potential of targeting the CYP4X1/sEH‐14,15‐EET‐EA‐GPR119 axis needs to be validated in patient‐derived tumor models. Additionally, obesity and type 2 diabetes are recognized risk factors for several cancer types, including colon cancer. GPR119 agonists provide pharmacological benefits for treating obesity and type 2 diabetes, prompting the question that GPR119 blockade may increase the risk of developing obesity and type 2 diabetes. Further investigations are warranted to examine GPR119 inhibition and its potential impact on anti‐tumor immunity in obesity‐associated tumor models. Lastly, AEA can be metabolized to produce 14,15‐EET‐EA by CYP2J2, CYP2D6, and CYP3A4 in addition to CYP4X1. Thus, the inhibition of CYP4X1 may increase the catalytic activity of these alternative CYP450 isoforms, sustaining 14,15‐EET‐EA production and compromising the efficacy of CYP4X1‐targeted therapies. Further studies are required to clarify this possibility.

In summary, CYP4X1/sEH‐dependent AEA metabolism promotes tumor immunosuppression through the upregulation of PD‐L1, CXCL12, and TGF‐β in CAFs via the 14,15‐EET‐EA‐GPR119‐Gs/β‐arrestin 2 signaling (Figure S25, Supporting Information). Our results delineate a previously unexplored lipidic interaction between CAFs and colon cancer cells, linking this novel crosstalk to tumor immunosuppression. Our findings may provide promising strategies for improving anti‐PD‐1 therapy in human colon cancer.

## 4 Experimental Section

### Human Specimens

Human colon cancer tissue microarray (HColAde180Sur‐05) containing 90 patients with colon cancer and adjacent normal tissues was obtained from Shanghai Outdo Biotech Co., Ltd. Human colon cancer samples were collected from Zhongnan Hospital and Renmin Hospital of Wuhan University. The study was conducted in accordance with the Declaration of Helsinki and approved by the Ethical Committee of the Medical School of Wuhan University. The ethics approval number is WHU‐LFMD‐IRB2025035.

### Mice

All animal experiments were approved by the animal ethics committee of the Animal Research Committee of Wuhan University, and maintained in accordance with the guidelines of the Association for Assessment and Accreditation of Laboratory Animal Care International. C57BL/6 (male, 6–8 weeks old) and BALB/c mice (male, 6–8 weeks old) were purchased from the Centers for Disease Control and Prevention (Hubei, China). All mice were housed in a pathogen‐free environment on a 12 h light/dark cycle with ad libitum access to water and standard rodent diet.

### Tumor Models and Treatments

For the subcutaneous colon tumor challenge, CT26 cells (2 × 10^5^) with *Cyp4x1* knockdown (*Cyp4x1* ^KD^), *Ephx2* overexpression (*Ephx2* ^OE^), or their combination (*Cyp4x1* ^KD^ *Ephx2* ^OE^) were subcutaneously injected into the left flank of BALB/c mice. For the orthotopic colon tumor challenge, MC38 tumor chunks derived from *Cyp4x1* ^KD^‐, *Ephx2* ^OE^‐, or *Cyp4x1* ^KD^ *Ephx2* ^OE^‐expressed MC38 cells were implanted into the cecum wall via absorbable surgical sutures as previously described.^[^ ^60^ ^]^ The animals were euthanized on day 21, and the tumor tissues were collected for further analysis.

The subcutaneous MC38 colon cancer model was used to evaluate whether pharmacological regulation of CYP4X1 and sEH could improve colon cancer immunosuppression. In brief, MC38 cells were subcutaneously inoculated into the left flank of each mouse. After 7 days, the mice were administered with CYP4X1 inhibitor CH625 (25 mg kg^−1^ daily, i.p.) and sEH inducer clofibrate (40 mg kg^−1^ daily, i.p.). The doses of CH625 and clofibrate were selected according to previously published studies.^[^ ^20^, ^61^ ^]^ 24 h after the last injection, the mice were euthanized, and the tumors were collected and analyzed.

For the CD8^+^ T cell depletion experiment, mice were intraperitoneally injected with 200 µg of CD8α antibody (BioXCell, #BE0061) every 3 days. MC38 cells (2 × 10^5^) with or without *Cyp4x1* ^KD^ *Ephx2* ^OE^ expression were subcutaneously injected into the left flank of mice 2 days after the first time of anti‐CD8α antibody injection. Mice were euthanized 15 days after tumor cell implantation, and the tumor tissues were collected and analyzed.

For the Treg depletion experiment, anti‐mouse CD25 monoclonal antibody (BioXCell, #BE0012) was intraperitoneally injected into mice (150 µg per mouse) twice weekly, starting 1 week before tumor inoculation, with a total of four injections following a previously published protocol.^[^ ^62^ ^]^ Mice were euthanized 15 days after tumor cell implantation, and the tumor tissues were excised and analyzed.

For the 14,15‐EET‐EA supplementation experiment, MC38‐luciferase cells (2 × 10^5^) expressing *Cyp4x1* ^KD^ *Ephx2* ^OE^ were subcutaneously injected into the left flank of C57BL/6 mice, followed by supplementation with or without 14,15‐EET‐EA (30 µg kg^−1^ every 2 days, i.v.) starting on day one after tumor cell inoculation. The dose of 14,15‐EET‐EA was selected according to the preliminary experiments. Tumor growth was monitored weekly using in vivo bioluminescence imaging, and fluorescence analysis was conducted with Living Image software (PerkinElmer). After 21 days, the mice were euthanized, and the tumors were isolated for further study.

For the in vivo co‐injection experiment, MC38 cells with or without *Cyp4x1* ^KD^ *Ephx2* ^OE^ expression were mixed with GFP^+^ L929 fibroblasts, and MC38 cells were mixed with vehicle‐ or 14,15‐EET‐EA‐treated GFP^+^ L929 fibroblasts at a 1:5 ratio in 200 µL of PBS and subcutaneously co‐injected into the left flank of C57BL/6 mice. Two weeks after inoculation, tumors were isolated for further study.

For the in vivo CAF function inhibition experiment, tranilast (a CAF suppressor) was employed.^[^ ^26^, ^48^ ^]^ Briefly, C57BL/6 mice were subcutaneously injected with MC38 cells (2 × 10^5^) in the left flank, and then treated with 14,15‐EET‐EA (30 µg kg^−1^ every 2 days, i.v.), tranilast (200 mg kg^−1^ daily, i.g.), or their combination for 2 weeks on day 7 after tumor cell implantation. The mice were sacrificed on day 21, and the tumors were collected for further evaluation.

The effects of CYP4X1/sEH on PD‐1 blockade therapy were evaluated in subcutaneous colon cancer models. CT26 cells (2 × 10^5^) or MC38 (2 × 10^5^) cells with or without *Cyp4x1* ^KD^ *Ephx2* ^OE^ expression were subcutaneously injected into the left flank of mice. On day 7 post‐implantation, mice were treated with anti‐PD‐1 antibody (200 µg mouse^−1^, i.p.) or control IgG every 3 days for 4 times. Mice were sacrificed 3 weeks after tumor cell inoculation, and tumors were collected for further evaluation.

To evaluate the effects of 14,15‐EET‐EA on PD‐1 blockade therapy, MC38 cells (2 × 10^5^) were subcutaneously inoculated into the left flank of each mouse. After 7 days, the mice were administered 14,15‐EET‐EA (30 µg kg^−1^ every 2 days, i.v.), anti‐PD‐1 antibody (200 µg mouse^−1^ every 3 days, i.p.), or a combination of both. After euthanizing the mice on day 21, the tumors were collected and analyzed.

Pharmacological and genetic approaches were used to evaluate the effects of GPR119 on PD‐1 blockade therapy. The established chitosan nanoparticles were adopted for *Gpr119* siRNA delivery.^[^ ^60^ ^]^ For orthotopic injection, MC38 cells were dispersed in a 20 µL mixture of DMEM medium and Matrigel (3:1, v/v) and inoculated into the cecal submucosa using a 30 G insulin syringe. On day 7 post‐implantation, mice were treated with anti‐PD‐1 antibody (200 µg mouse^−1^ every 3 days, i.p.), or *Gpr119* siRNA (250 µg kg^−1^ every 3 days, i.v.). Mice were sacrificed 3 weeks after tumor cell inoculation, and tumors were collected for further evaluation. For pharmacological inhibition, mice were subcutaneously inoculated with MC38 (2 × 10^5^) or CT26 (2 × 10^5^) cells in the left flank. Seven days later, the mice were treated with anti‐PD‐1 antibody (200 µg mouse^−1^ every 3 days, i.p.), arvanil (1 mg kg^−1^ every 3 days, i.p.), or a combination of both. The dose of arvanil was selected according to the preliminary experiments. For the in vivo co‐injection experiment, sh‐NC or sh*Gpr119*‐treated L929 cells were co‐injected with MC38 cells to establish a subcutaneous colon cancer model. On day 7 post‐implantation, mice were treated with anti‐PD‐1 antibody (200 µg mouse^−1^, i.p.) or control IgG every 3 days for 4 times. For pharmacological activation, mice were subcutaneously inoculated with MC38 cells in the left flank. 7 days later, the mice were treated with an anti‐PD‐1 antibody (200 µg mouse^−1^ every 3 days, i.p.), a GPR119 agonist AR231453 (3 mg kg^−1^ every 2 days, i.p.), or a combination of both. The dose of AR231453 was selected according to the preliminary experiments. 24 h after the last injection, the mice were euthanized, and the tumors were collected and analyzed.

### Multiplex Immunofluorescence Analysis

Multiplex immunofluorescence staining (mIF) was conducted to characterize the immune landscape in human colon cancer tissues using Opal Polaris 7 Color IHC Manual Detection Kit (#NEL861001KT, Akoya Bioscience, USA) according to the manufacturer's protocol. Briefly, formalin‐fixed and paraffin‐embedded tumor sections were deparaffinized and rehydrated, followed by antigen retrieval with citric acid buffer (pH 6.0)/Tris‐EDTA buffer (pH 9.0), and then blocked in Opal Antibody Diluent/Block diluent. Next, slides were incubated with primary antibodies against CYP4X1 (1:100, Invitrogen, #PA5‐101319), sEH (1:200, Proteintech, #10833‐1‐AP), GPR119 (1:50, Affinity, #DF4892), α‐SMA (1:300, BOSTER, #BM0002), FOXP3 (1:400, Cell Signaling Technology, #12653), CD31 (1:300, Santa Cruz, #sc‐376764), CD8 (1:2, DAKO, #IR623), cytokeratin (CK, 1:2, DAKO, #IS05330‐2), PDPN (1:200, Huabio, #ET1703‐61), HLA‐DRA (1:1000, Huabio, #ET1610‐66), or CXCL12 (1:100, Huabio, #ER1902‐35). Each primary antibody was visualized using tyramide signal amplification linked to a specific fluorochrome from the mIF kit. Once all markers were stained, spectral DAPI was used to counterstain the slides. Multiplex‐stained slides were scanned using a Vectra 3 multispectral microscope (Akoya Bioscience) at 20 nm wavelength intervals from 420 nm to 720 nm with the same fixed exposure time. The images were sequentially spectrally unmixed using InForm 2.7 software.

### Human Colon Cancer Tissue Microarray Analysis

Human colon cancer tissue microarray (HColAde180Sur‐05) containing 90 patients with colon cancer and adjacent normal tissues was obtained from Shanghai Outdo Biotech Co., Ltd. Immunohistochemistry (IHC) was conducted by using the sEH (1:200) and CYP4X1 (1:100) antibodies to investigate the expression levels of sEH and CYP4X1 proteins in human colon cancer tissues. Immunostaining was graded by multiplying the positivity score and intensity score as previously described.^[^ ^63^ ^]^ Two pathologists independently evaluated the staining status and reached a consensus on the grading. Patients were divided into high‐ and low‐expression groups according to the median IHC score.

### Tumor Perfusion Measurement

Tumor perfusion was assessed by laser Doppler analysis according to a previously described method.^[^ ^48^ ^]^ In brief, MC38 or CT26 tumor‐bearing mice, as described above, were anesthetized with 0.6% pentobarbital and maintained at a temperature of 37 °C during the imaging. The cutaneous envelope over each tumor was carefully excised to preserve the vascular network. Then, the tumor perfusion in the mice was blindly evaluated utilizing laser Doppler perfusion imaging (LDPI; Moor Instruments). Tumor perfusion in arbitrary perfusion units was graphically monitored.

Chemicals and reagents; cell culture; ELISA; IHC and IF staining; flow cytometry analysis; migration assays; tumor conditioned medium preparation; lentiviral transfection; isolation of mouse CAFs; AEA, OEA, PEA, and 14,15‐EET‐EA content measurement; real‐time qPCR; western blot analysis; cAMP measurement; molecular docking; cellular thermal shift assay; β‐arrestin recruitment experiment and bioinformatics analysis were described in Supplementary Materials and Methods.

### Statistical Analysis

Statistical analysis and visualization were performed using R software (version 4.0.1) and GraphPad Prism (version 9.0.1). All described results were representative of at least three independent experiments, and all values were recorded as mean ± standard error of the mean (SEM). Statistical significance between two groups was determined using a two‐tailed Student's t‐test (normal distribution) or a Mann‐Whitney test (abnormal distribution). One‐way ANOVA followed by Tukey's multiple comparison test was used for the multiple comparisons. For non‐normally distributed data, the Kruskal‐Wallis test was used to compare three or more groups. The overall survival (OS), recurrence‐free survival (RFS), disease‐free survival (DFS), and progression‐free survival (PFS) rates were estimated using the Kaplan‐Meier method, and the statistical significance was determined using the log‐rank test. *P* values < 0.05 were considered significant.

### Ethical Approval Statement

The studies involving humans were approved by the Ethical Committee of the Medical School of Wuhan University. The ethics approval number is WHU‐LFMD‐IRB2025035. Informed consent was obtained from all patients before surgery and participation in this study. All animal experiments were conducted in accordance with the approved protocol and other relevant guidelines and regulations.

## Conflict of Interest

The authors declare no conflict of interest.

## Author contributions

M.M. conceived and designed the study and finalized the manuscript. X.C., Y.L., C.W., and X.C. contributed to the statistical analyses, and writing‐review and editing. N.Z., N.H., J.W., and Y.L. performed the experiments. H.C. contributed to the acquisition of the data, provided technical assistance, and edited the draft. J.Y. initiated and directed the whole project, revised and finalized the manuscript. All the authors drafted the manuscript. The manuscript has been reviewed and approved by all authors.

## Supporting information



Supporting Information

## Data Availability

The data that support the findings of this study are available from the corresponding author upon reasonable request.
