# Supplementary material for: CYP4X1/sEH‐Dependent Endocannabinoid Metabolism Drives Fibroblast‐Mediated Immunosuppression to Limit Immunotherapy in Colon Cancer
Source: Adv Sci (Weinh). 2025 Nov 23;13(5):e07695. doi: 10.1002/advs.202507695 (PMC12849875; doi:10.1002/advs.202507695)
Supplement: Supplementary file 1 — Supporting Information [file ADVS-13-e07695-s001.docx]

**Supporting Information**

**CYP4X1/sEH-Dependent Endocannabinoid Metabolism** **Drives Fibroblast-Mediated Immunosuppression to Limit Immunotherapy in Colon Cancer**

*Min Mo, Xuewei Chen, Yanzhuo Liu, Chenlong Wang, Xuehan Chen, Nan Zhang, Nan He,* *Ying Li, Jingyi Wang, Honglei Chen^☆^, Jing Yang^☆^*

The supporting information includes:

1) Figure S1 to S25

2) One supplementary table

3) Supplementary materials and methods


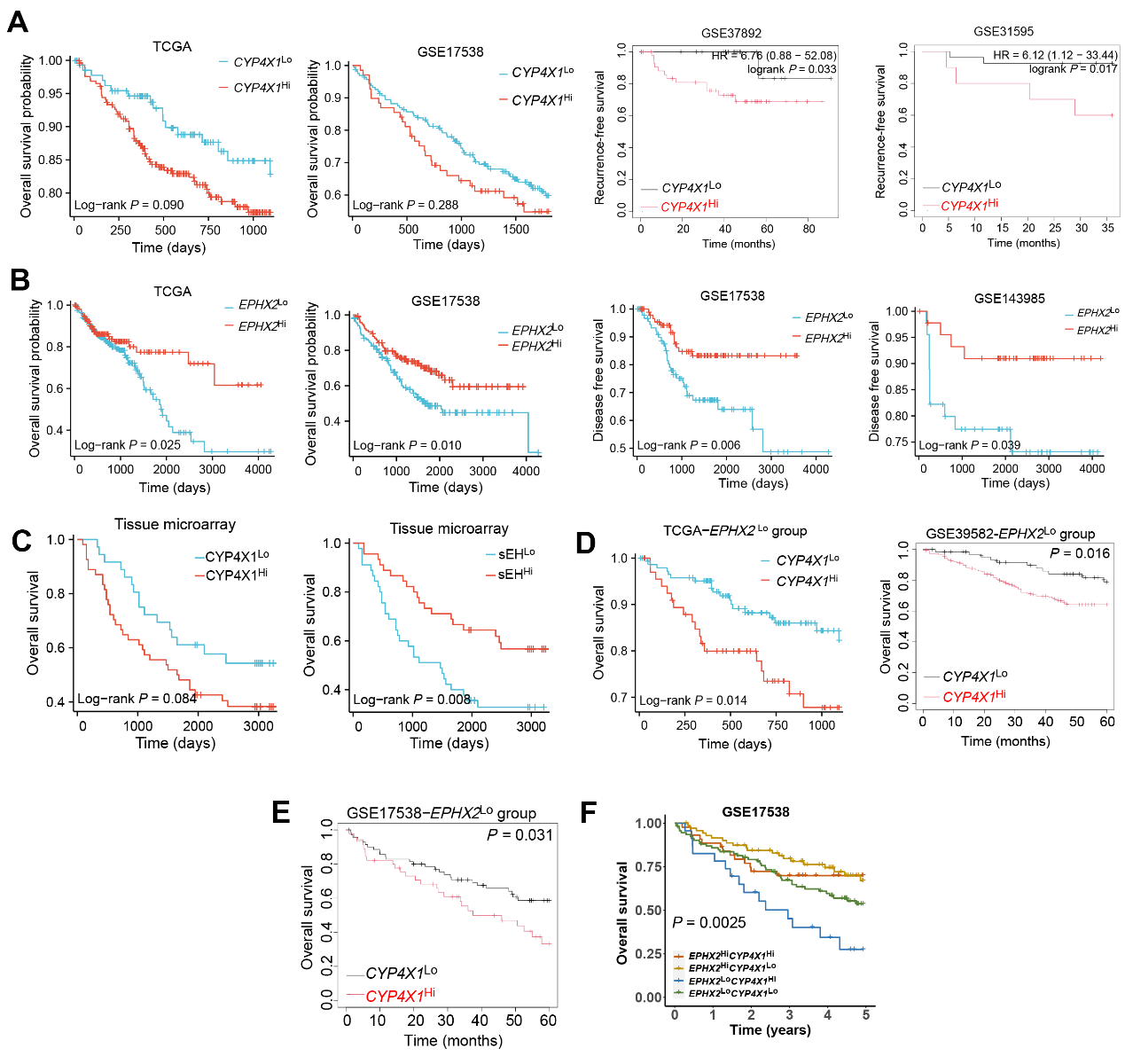


**Figure S1.** CYP4X1 and sEH jointly predict prognosis in human colon cancer. A) Kaplan-Meier analysis of overall survival (OS) and recurrence-free survival in patients with colon cancer stratified by *CYP4X1* gene expression (TCGA-COAD, GSE17538, GSE37892, and GSE31595). B) Kaplan-Meier analysis of OS and disease-free survival in patients with colon cancer stratified by *EPHX2* gene expression (TCGA-COAD, GSE17538, and GSE143985). C) Kaplan-Meier OS curves of CYP4X1- and sEH-high/low groups in human colon cancer tissue microarrays (*n* = 90). Patients were divided into high- and low-expression groups using the median IHC score as the cutoff point. D,E) Kaplan-Meier analysis of OS in colon cancer patients with low *EPHX2* expression, stratified by *CYP4X1* gene expression (TCGA-COAD, GSE39582, and GSE17538). F) Kaplan-Meier survival curves of OS among four groups stratified by *CYP4X1* and *EPHX2* gene expression in the GSE17538 dataset. *P* values were determined using log-rank tests.


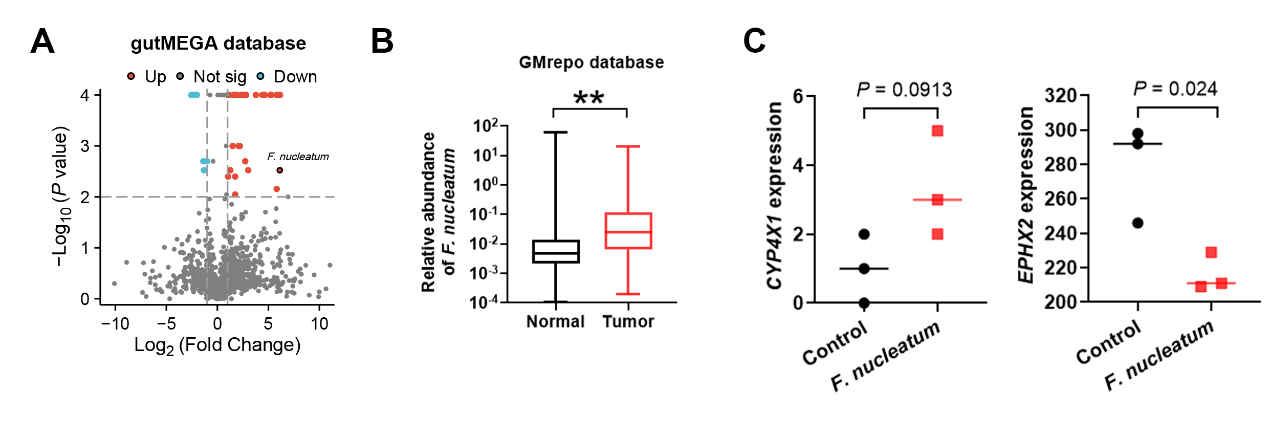


**Figure S2.** *F. nucleatum* is correlated with *CYP4X1* and *EPHX2* gene expression in colorectal cancer patients. A) The volcano plot displays the differences in the microbiota abundance at the species level between colorectal cancer patients and healthy individuals in the gutMEGA database. B) The relative fecal abundance of *F. nucleatum* in healthy individuals and patients with colorectal cancer in the GMrepo database. C) *CYP4X1* and *EPHX2* gene expression levels in HCT116 colon cancer cells with or without *F. nucleatum* treatment in the GSE141805 dataset (*n* = 3). *P* values were determined using the Mann-Whitney test (B) or Student's t-test (C). ^**^ *P* < 0.01.


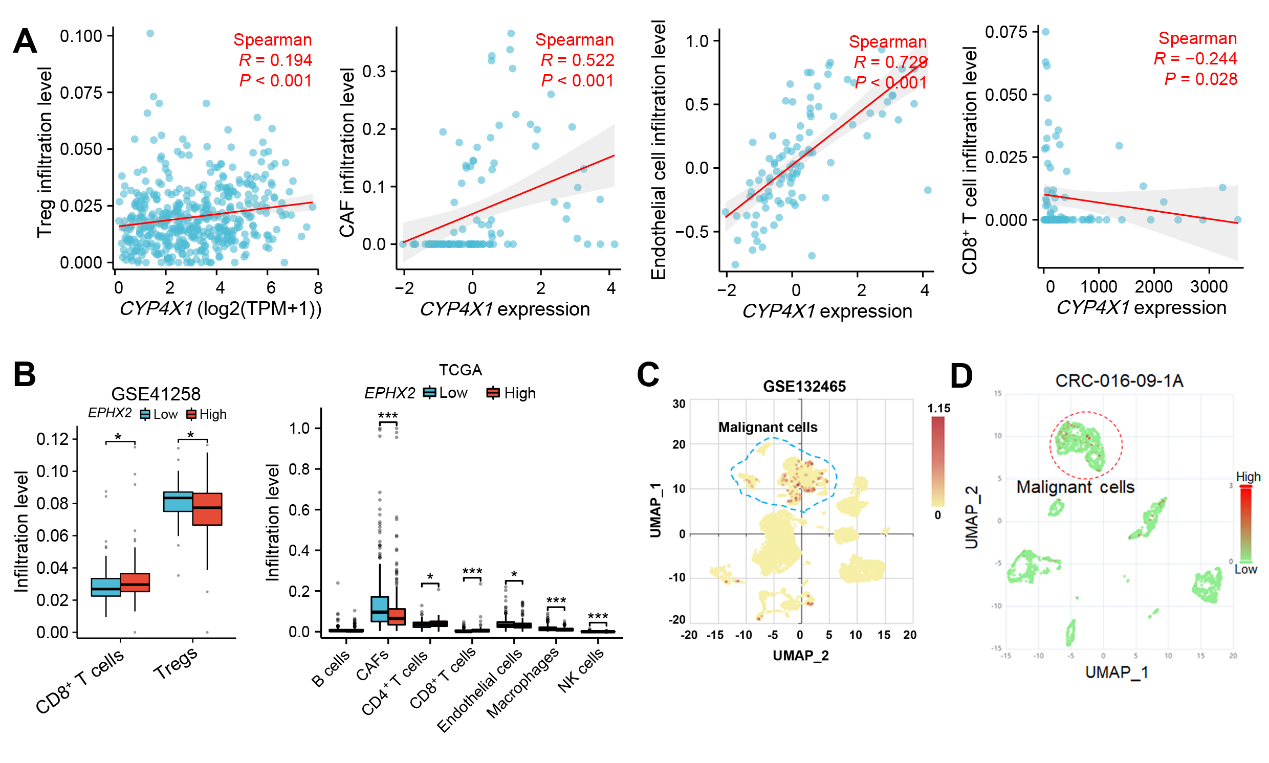


**Figure S3.** Correlation analysis of *CYP4X1* and *EPHX2* expression with immune and stromal cell infiltration and scRNA-seq analysis of *CYP4X1* and *EPHX2* expression in human colon cancer tissues. A) Correlation analysis of *CYP4X1* expression with immune and stromal cell infiltration in TCGA-COAD, GSE33193, and GSE64857 datasets. B) Correlation analysis of *EPHX2* expression with immune and stromal cell infiltration in GSE41258 and TCGA-COAD datasets. C) ScRNA-seq analysis of *CYP4X1* expression in human colorectal cancer tissues (GSE132465). D) ScRNA-seq analysis of *EPHX2* expression in human colorectal cancer tissues using the CancerSCEM database. *P* values were determined using Mann-Whitney tests (B). ^*^ *P* < 0.05 and ^***^ *P* < 0.001.


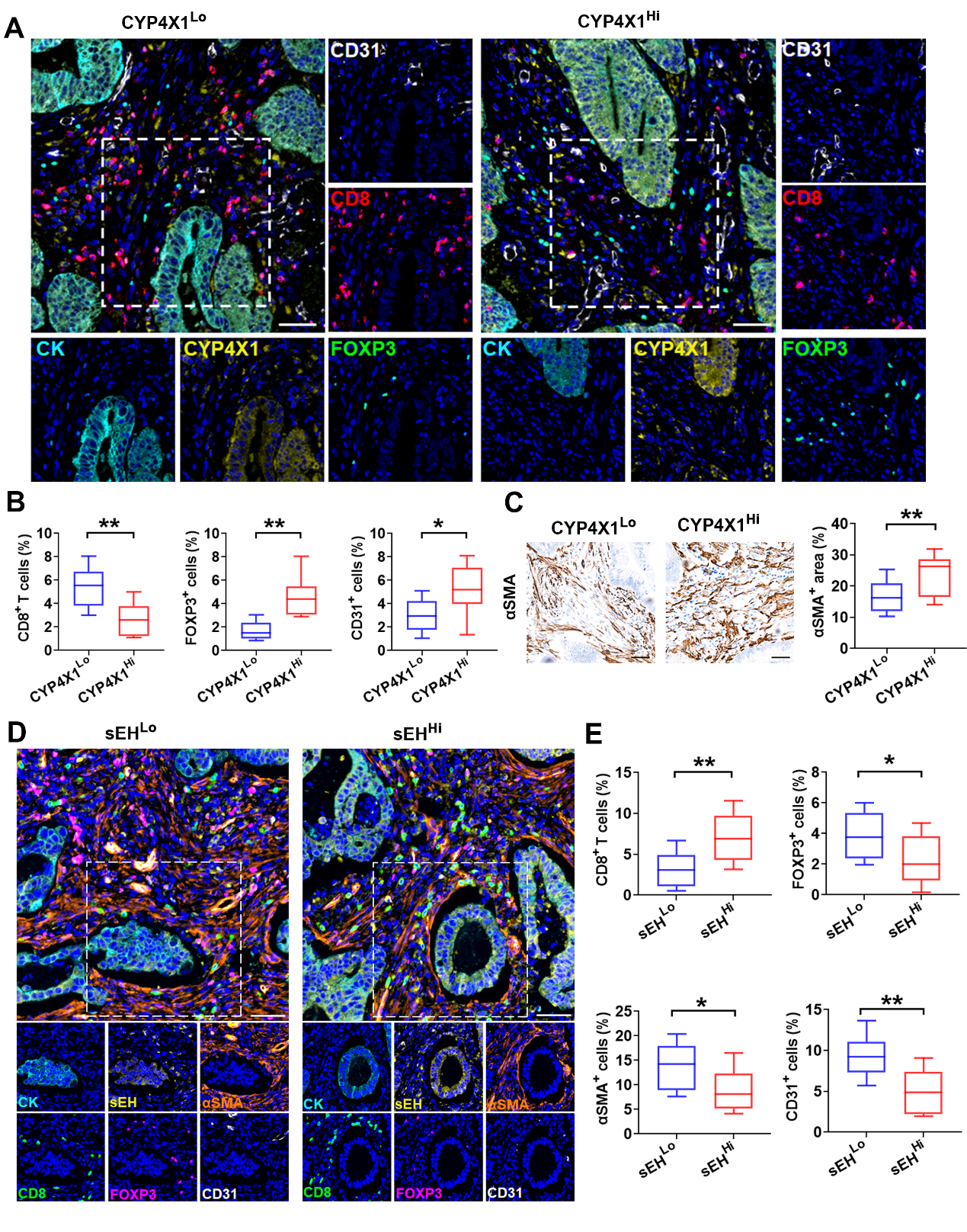


**Figure S4.** High CYP4X1 or low sEH expression is correlated with immunosuppressive TME in human colon cancer. A) Representative images of multiplex immunofluorescence (mIF) staining in CYP4X1^Lo^ and CYP4X1^Hi^ human colon cancer tissues. Scale bar, 50 μm. B) Quantification of CD8^+^ T cells, FOXP3^+^ Tregs, and CD31^+^ endothelial cells as a proportion of total cells. C) Representative IHC staining and quantification of α-SMA in human colon cancer tissues with CYP4X1^Lo^ or CYP4X1^Hi^ expression. Scale bars, 50 μm. D) Representative images of mIF staining in sEH^Lo^ and sEH^Hi^ human colon cancer tissues. Scale bar, 50 μm. E) Quantification of CD8^+^ T cells, FOXP3^+^ Tregs, α-SMA^+^ CAFs, and CD31^+^ endothelial cells as a proportion of total cells. *n* = 10. *P* values were determined using unpaired Student's t-tests. ^*^ *P* < 0.05; ^**^ *P* < 0.01.


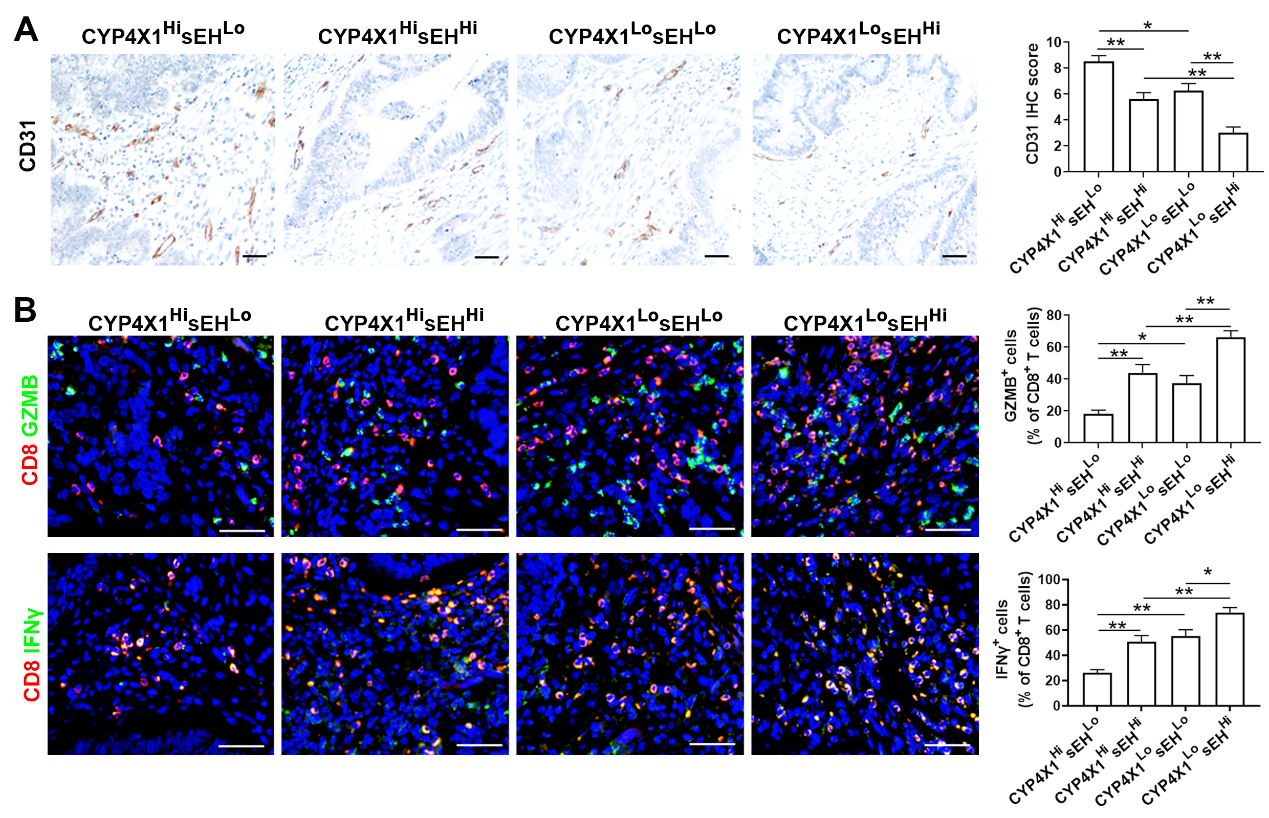


**Figure S5.** Joint effects of CYP4X1 and sEH on angiogenesis and CD8^+^ T cell function. A) Representative images and quantification of IHC staining of CD31 in human colon cancer tissues. Scale bar, 50 μm. B) Representative images and quantification of IF staining of CD8 (red) and GZMB (green) or IFN-γ (green) in human colon cancer tissues. Scale bar, 50 μm. Data are presented as mean ± SEM, *n* = 8. *P* values were determined using one-way ANOVA. ^*^ *P* < 0.05 and ^**^ *P* < 0.01.


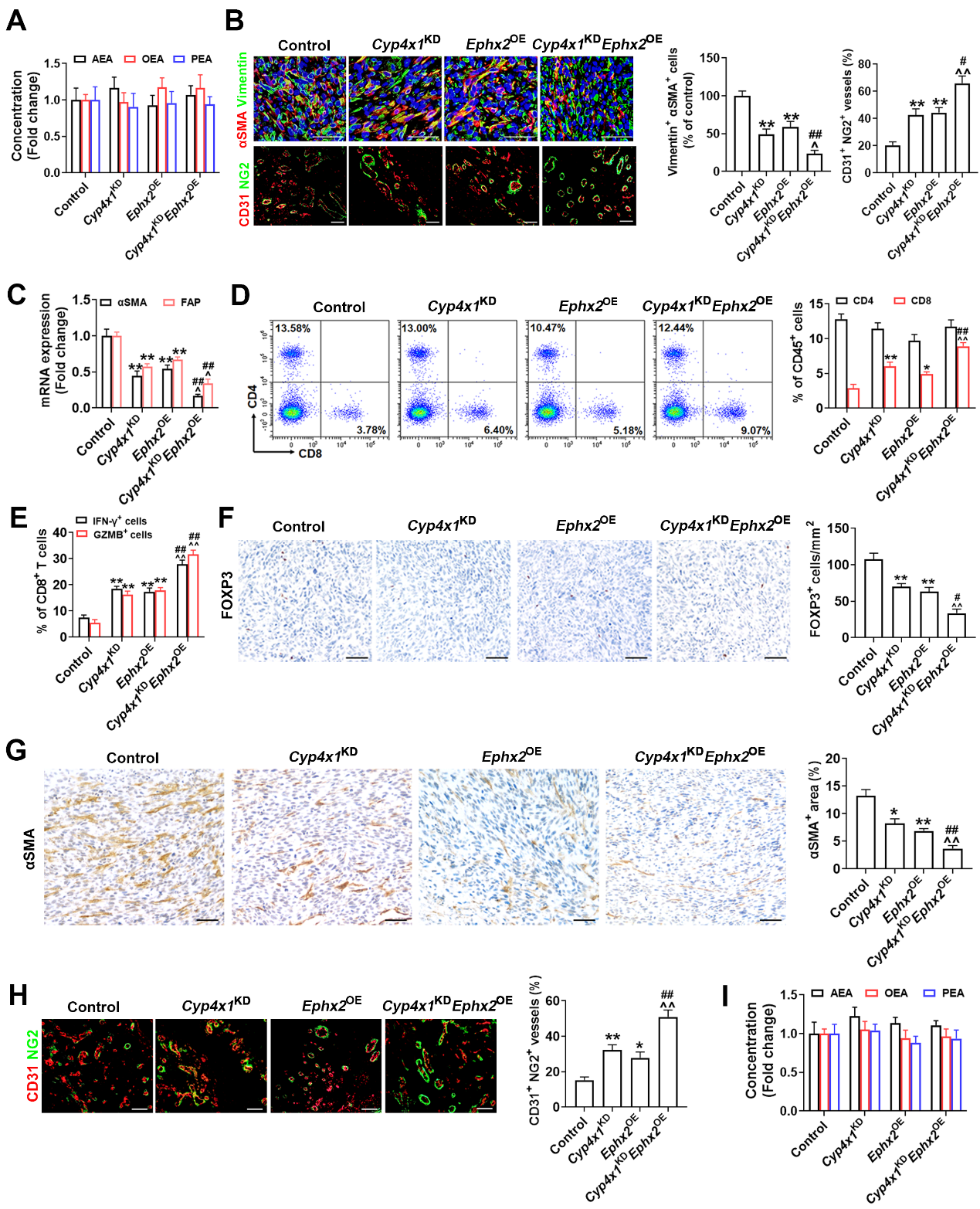


**Figure S6.** CYP4X1 knockdown or EPHX2 overexpression improves colon cancer immune microenvironment. A) AEA, OEA, and PEA levels in orthotopic MC38 tumor tissues (*n* = 4). B) IF analysis of CAF activation and tumor vessel normalization in orthotopic MC38 colon cancer tissues (*n* = 5). Scale bar, 50 μm. C) αSMA and FAP mRNA levels in the purified CAFs from orthotopic MC38 tumor tissues (*n* = 5). CT26 colon cancer cells with *Cyp4x1*^KD^, *Ephx2*^OE^, or *Cyp4x1*^KD^*Ephx2*^OE^ expression were subcutaneously implanted into BALB/c mice (*n* = 8). D) The percentages of CD4^+^ T and CD8^+^ T cells in subcutaneous CT26 colon cancer tissues were analyzed by flow cytometry. E) Percentages of GZMB^+^ CD8^+^ T cells and IFN-γ^+^ CD8^+^ T cells in CT26 tumor tissues. F) IHC staining and quantification of FOXP3 in CT26 tumor tissues. Scale bar, 50 μm. G) Representative IHC staining and quantification of α-SMA in CT26 tumor tissues. Scale bars, 50 μm. H) IF analysis of tumor vessel normalization in CT26 tumors. Scale bar, 50 μm. I) AEA, OEA, and PEA levels in CT26 tumor tissues (*n* = 5). Data are presented as mean ± SEM, *n* = 8. *P* values were determined using one-way ANOVA. ^*^ *P* < 0.05 and ^**^ *P* < 0.01 vs. control; ^^^ *P* < 0.05 and ^^^^ *P* < 0.01 vs. *Cyp4x1*^KD^; ^#^ *P* < 0.05 and ^##^ *P* < 0.01 vs. *Ephx2*^OE^ group.


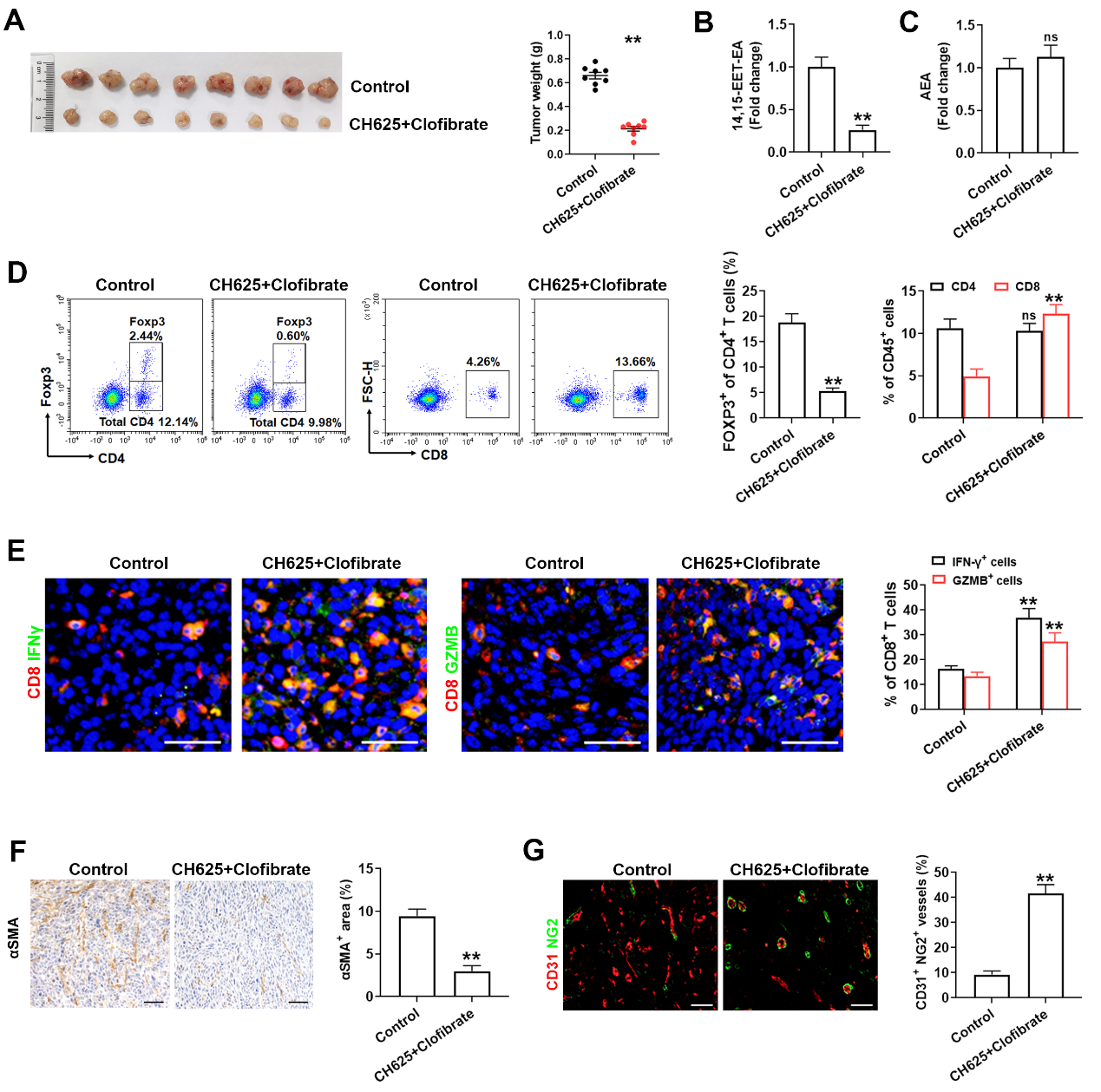


**Figure S7.** CYP4X1 inhibitor and sEH inducer improve colon cancer immune microenvironment. A) C57BL/6 mice inoculated with MC38 cells were treated with CYP4X1 inhibitor CH625 and sEH inducer clofibrate, or vehicle control. Tumor weights were measured in the indicated groups (*n* = 8). B) 14,15-EET-EA level was determined by liquid chromatography tandem-mass spectrometry (LC-MS/MS). C) AEA level in tumor tissues. D) Representative flow staining and quantification of Tregs, CD4^+^ T cells, and CD8^+^ T cells in tumor tissues of the indicated groups. E) Representative images and quantitative analysis of IF staining of CD8 (red) and IFN-γ (green) or GZMB (green) in tumor tissues. Scale bar, 50 μm. F) Representative IHC staining and quantification of α-SMA expression in tumor tissues. Scale bars, 50 μm. G) IF analysis of tumor vessel normalization in tumors. Scale bar, 50 μm. Data are presented as the mean ± SEM, *n* = 5. *P* values were determined using Student's t-tests. ^**^ *P* < 0.01 vs. control; ns, not significant.


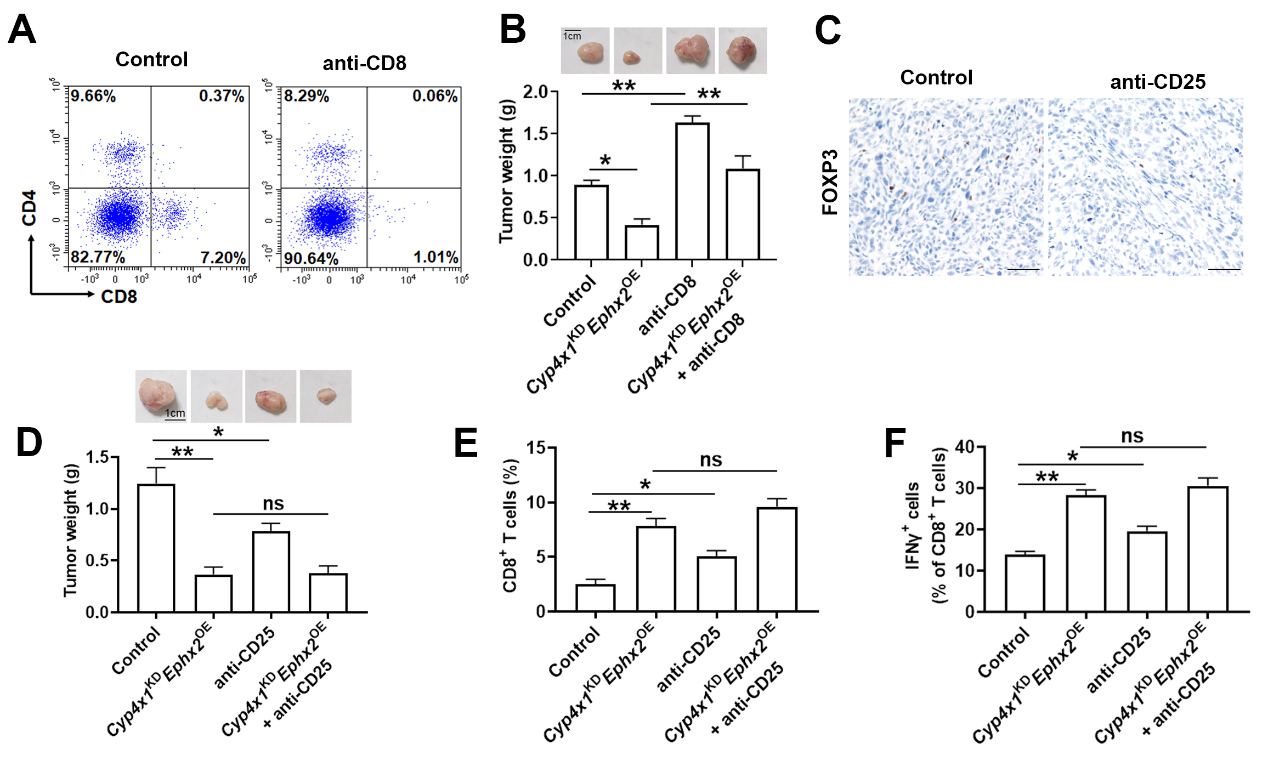


**Figure S8.** *Cyp4x1*^KD^*Ephx2*^OE^ mediates tumor rejection by inhibiting Treg infiltration and enhancing antitumoral CD8^+^ T-cell immunity. A) Flow cytometry analysis of CD8^+^ T cell percentage after anti-CD8 antibody treatment. B) Tumor weights of indicated groups. C) IHC staining of FOXP3 in MC38 tumors with or without anti-CD25 antibody treatment. Scale bar, 50 μm. D) Tumor weights of indicated groups. E) The percentage of CD8^+^ T cells was determined by flow cytometry. F) The percentage of IFN-γ^+^ CD8^+^ T cells in tumors was determined by flow cytometry. Data are presented as mean ± SEM, *n* = 6. *P* values were determined using one-way ANOVA (B and D-F). ^*^ *P* < 0.05; ^**^ *P* < 0.01; ns, not significant.


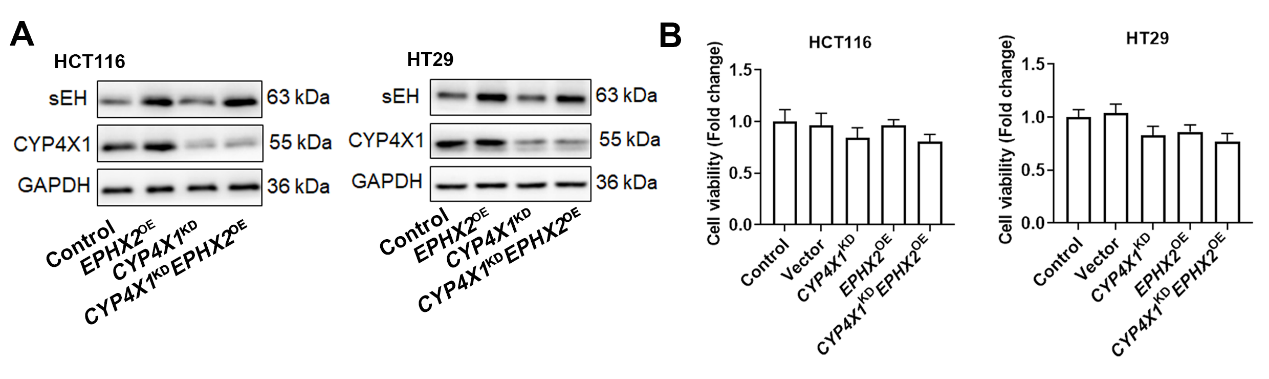


**Figure S9.** CYP4X1 knockdown and EPHX2 overexpression does not directly alter the proliferation of tumor cells. A) CYP4X1 and sEH protein levels in HCT116 and HT29 colon cancer cells with *CYP4X1* knockdown and/or *EPHX2* overexpression. B) Relative cell viabilities of *in vitro* cultured HCT116 and HT29 cells with *CYP4X1* knockdown and/or *EPHX2* overexpression compared with control cells. Data are presented as mean ± SEM, *n* = 5. *P* values were determined using one-way ANOVA.


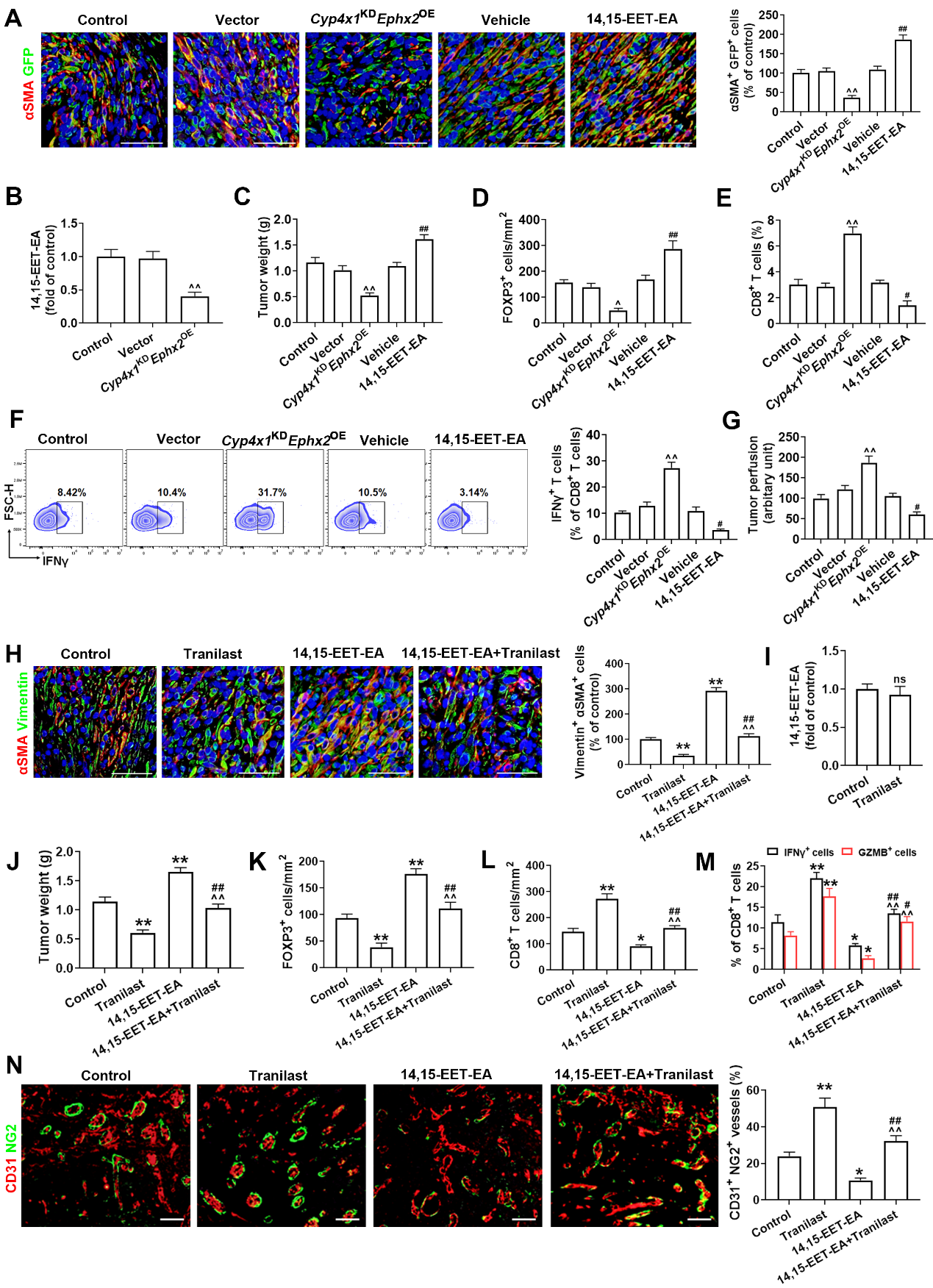


**Figure S10.** CYP4X1/sEH-derived 14,15-EET-EA drives tumor immune escape via CAFs. MC38 cells with or without *Cyp4x1*^KD^*Ephx2*^OE^ expression were mixed with GFP^+^ L929 fibroblasts, and MC38 cells were mixed with vehicle- or 14,15-EET-EA-treated GFP^+^ L929 fibroblasts at a 1:5 ratio in 200 µL of PBS and subcutaneously co-injected into the left flank of C57BL/6 mice. A) Representative IF staining and frequency analysis of αSMA (red) and GFP (green) in xenograft tumor tissues (*n* = 8). Scale bar, 50 μm. B) 14,15-EET-EA level was determined by LC-MS/MS (*n* = 8). C) Tumor weights in the indicated groups (*n* = 8). D) Treg accumulation in tumors was analyzed and quantified by IHC staining. E,F) The infiltration of CD8^+^ T cells and their expression of IFN-γ in tumor tissues of the indicated groups were determined by flow cytometry. G) Tumor perfusion in MC38 tumors of the indicated groups was measured using a laser Doppler analyzer. C57BL/6 mice were subcutaneously injected with MC38 cells in the left flank, and then treated with 14,15-EET-EA, tranilast, or their combination for 2 weeks starting on day 7 after tumor cell implantation. H) Representative IF staining and frequency of αSMA (red) and vimentin (green) in tumor tissues (*n* = 8). Scale bar, 50 μm. I) 14,15-EET-EA level was determined by LC-MS/MS (*n* = 8). J) Tumor weights in indicated groups (*n* = 8). K,L) The infiltration of CD8^+^ T cells and FOXP3^+^ cells in tumor tissues was analyzed. M) The percentages of GZMB^+^ CD8^+^ T cells and IFN-γ^+^ CD8^+^ T cells were analyzed by IF staining in tumor tissues. N) Representative IF staining and frequency analysis of CD31 (red) and NG2 (green) in tumor tissues from the indicated groups. Scale bar, 50 μm. Data are presented as mean ± SEM, *n* = 5. *P* values were determined using one-way ANOVA (A-H and J-N) or Student's t-test (I). ^*^ *P* < 0.05 and ^**^ *P* < 0.01 vs. control; ^#^ *P* < 0.05 and ^##^ *P* < 0.01 vs. vehicle or tranilast; ^^^ *P* < 0.05 and ^^^^ *P* < 0.01 vs. vector or 14,15-EET-EA; ns, not significant.


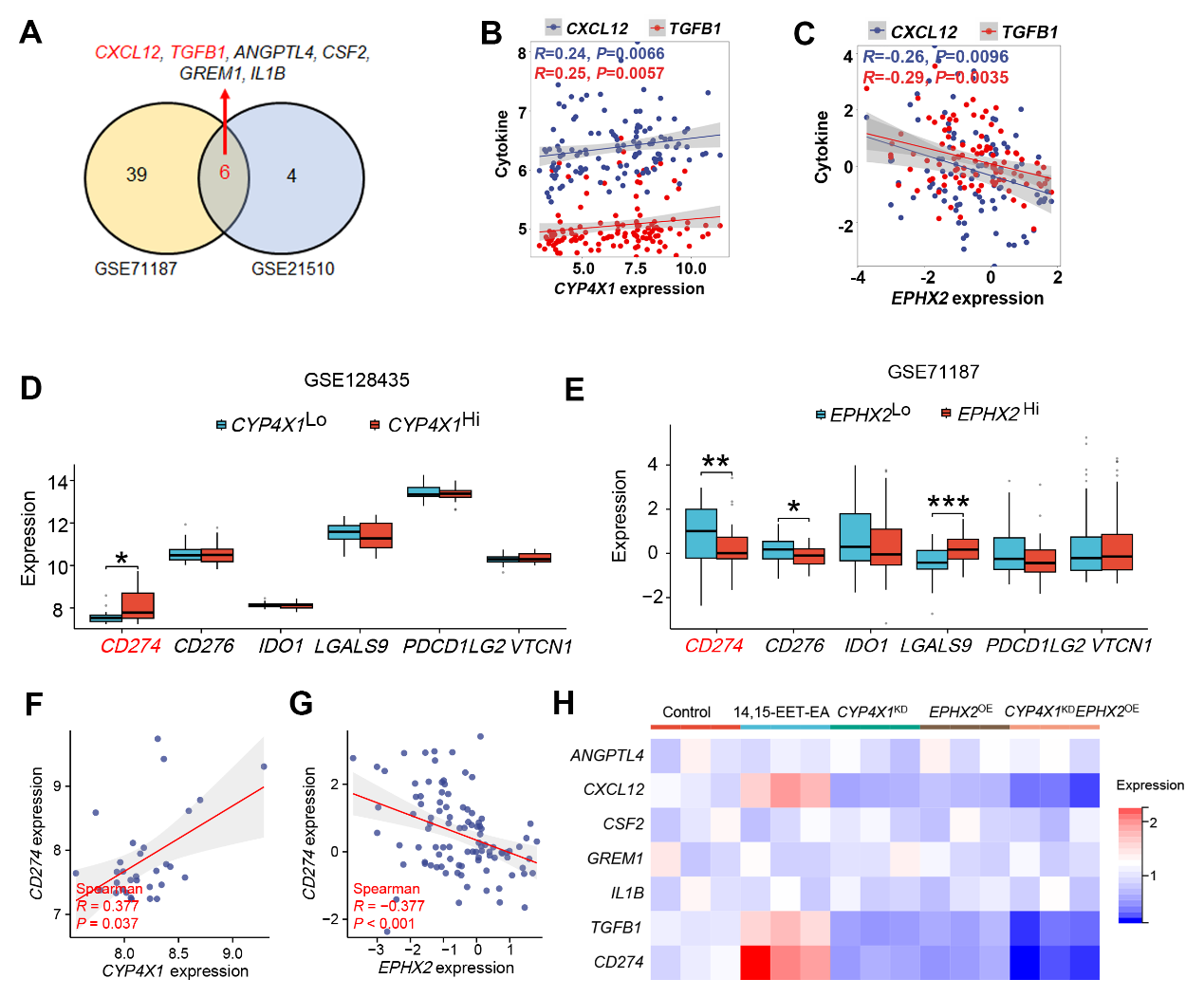


**Figure S11.** CYP4X1 knockdown or EPHX2 overexpression downregulates PD-L1, CXCL12, and TGF-β in CAFs. A) Venn diagram illustrating the overlap of cytokines concurrently correlated with *CYP4X1* and *EPHX2* expression in GEO datasets (GSE71187 and GSE21510). B) Spearman correlation analysis of *CYP4X1* with *CXCL12* and *TGFB1* of colon cancer patients in the GSE21510 dataset. C) Spearman correlation analysis of *EPHX2* with *CXCL12* and *TGFB1* of colon cancer patients in the GSE71187 dataset. D) Immune checkpoint gene expression levels in *CYP4X1*^Hi^ and *CYP4X1*^Lo^ expression groups (GSE128435). E) Immune checkpoint gene expression levels in *EPHX2*^Hi^ and *EPHX2*^Lo^ expression groups (GSE71187). F) Spearman correlation analysis of *CYP4X1* with *CD274* (PD-L1) of patients in the GSE128435 dataset. G) Spearman correlation analysis of *EPHX2* with *CD274* of patients in the GSE71187 dataset. H) The mRNA levels of immunosuppressive cytokines and coinhibitory molecules in MRC5 fibroblasts incubated with 14,15-EET-EA or the CM from HCT116 cells with *CYP4X1*^KD^, *EPHX2*^OE^, or *CYP4X1*^KD^*EPHX2*^OE^ expression (*n* = 3). *P* values were determined using Mann-Whitney tests (D and E). ^*^ *P* < 0.05; ^**^ *P* < 0.01; ^***^ *P* < 0.001.


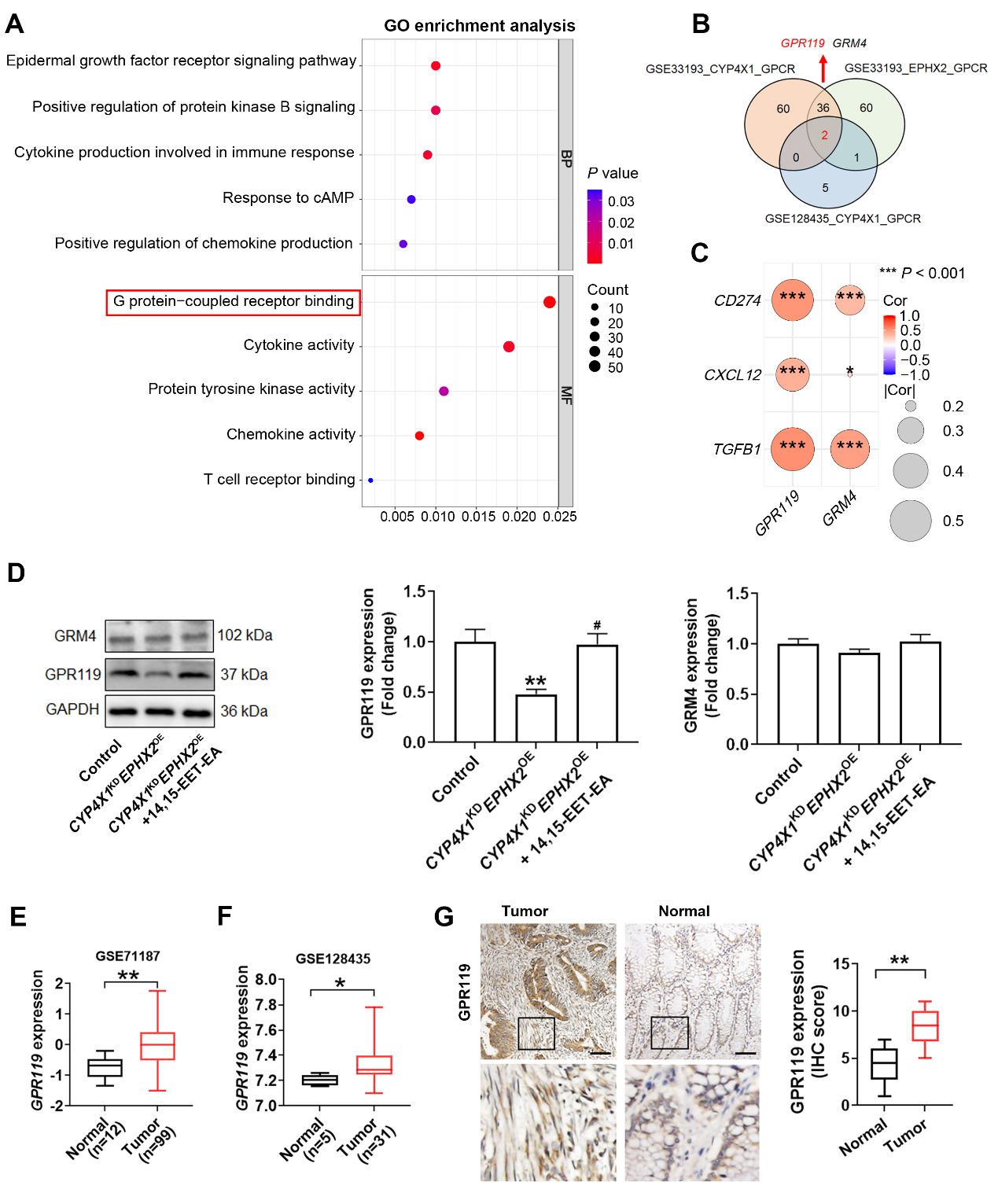


**Figure S12.** CYP4X1/sEH-derived 14,15-EET-EA induces immunosuppression via GPR119. A) Gene ontology (GO) enrichment analysis was performed using the differentially expressed genes (DEGs) between *CYP4X1*^Hi^*EPHX2*^Lo^ and *CYP4X1*^Lo^*EPHX2*^Hi^ groups in the TCGA-COAD dataset. B) Venn diagram depicting the intersection of GPCRs that were simultaneously correlated with the expression of *CYP4X1* and *EPHX2* in GEO datasets. C) Heatmap of correlation analysis of *GPR119* and *GRM4* with *CD274*, *CXCL12*, and *TGFB1* in the GSE75500 dataset. D) GPR119 and GRM4 protein levels in MRC5 fibroblasts grown in the *CYP4X1*^KD^*EPHX2*^OE^-derived CM with or without 14,15-EET-EA supplementation (*n* = 5). E,F) *GPR119* gene expression level in human colon cancer tissues and normal tissues from the GSE71187 and GSE128435 datasets. G) Representative IHC staining and quantification of GPR119 expression in human colon cancer tissues and adjacent normal tissues (*n* = 10). Scale bar, 100 μm. Data are presented as mean ± SEM. *P* values were determined using one-way ANOVA (D), Welch’s t test (E), Mann-Whitney test (F), or two-tailed paired Student's t-test (G). ^**^ *P* < 0.01 vs. control; ^#^ *P* < 0.05 vs. *CYP4X1*^KD^*EPHX2*^OE^ group.


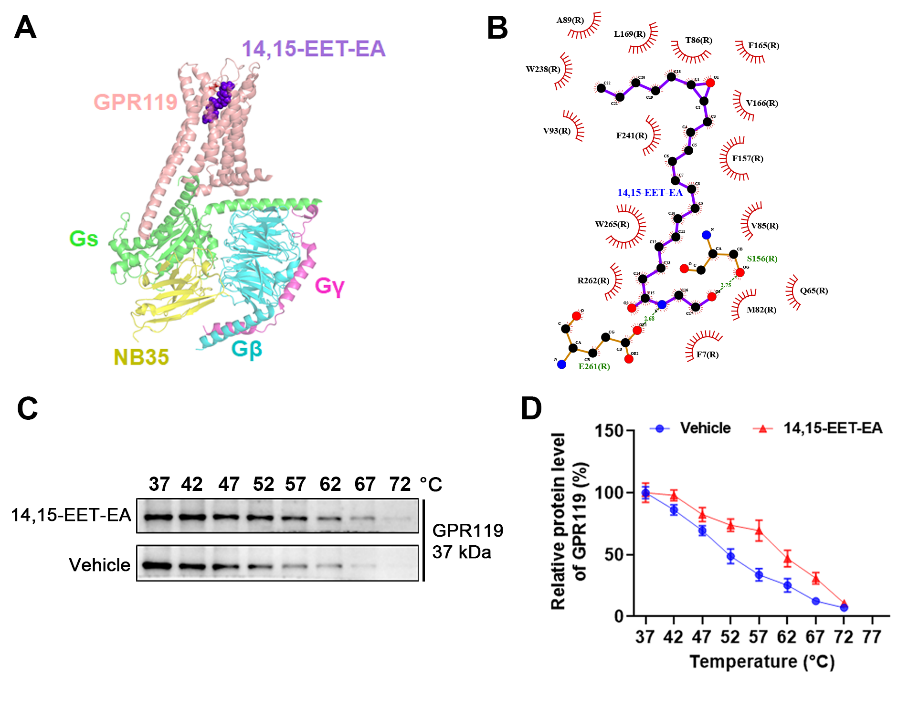


**Figure S13.** 14,15-EET-EA binds to GPR119 in CAFs. A) Molecular docking result of 14,15-EET-EA in the GPR119 ligand binding pocket. B) LigPlot diagram detailing the interaction of 14,15-EET-EA (purple ball-and-stick representation) with GPR119 receptor. Hydrogen bonds are shown as green dashed lines, and the sunburst icons represent hydrophobic interactions. C,D) The thermal stability of GPR119 protein in CAFs treated with or without 14,15-EET-EA was measured by CETSA. Data are presented as the mean ± SEM, *n* = 3.


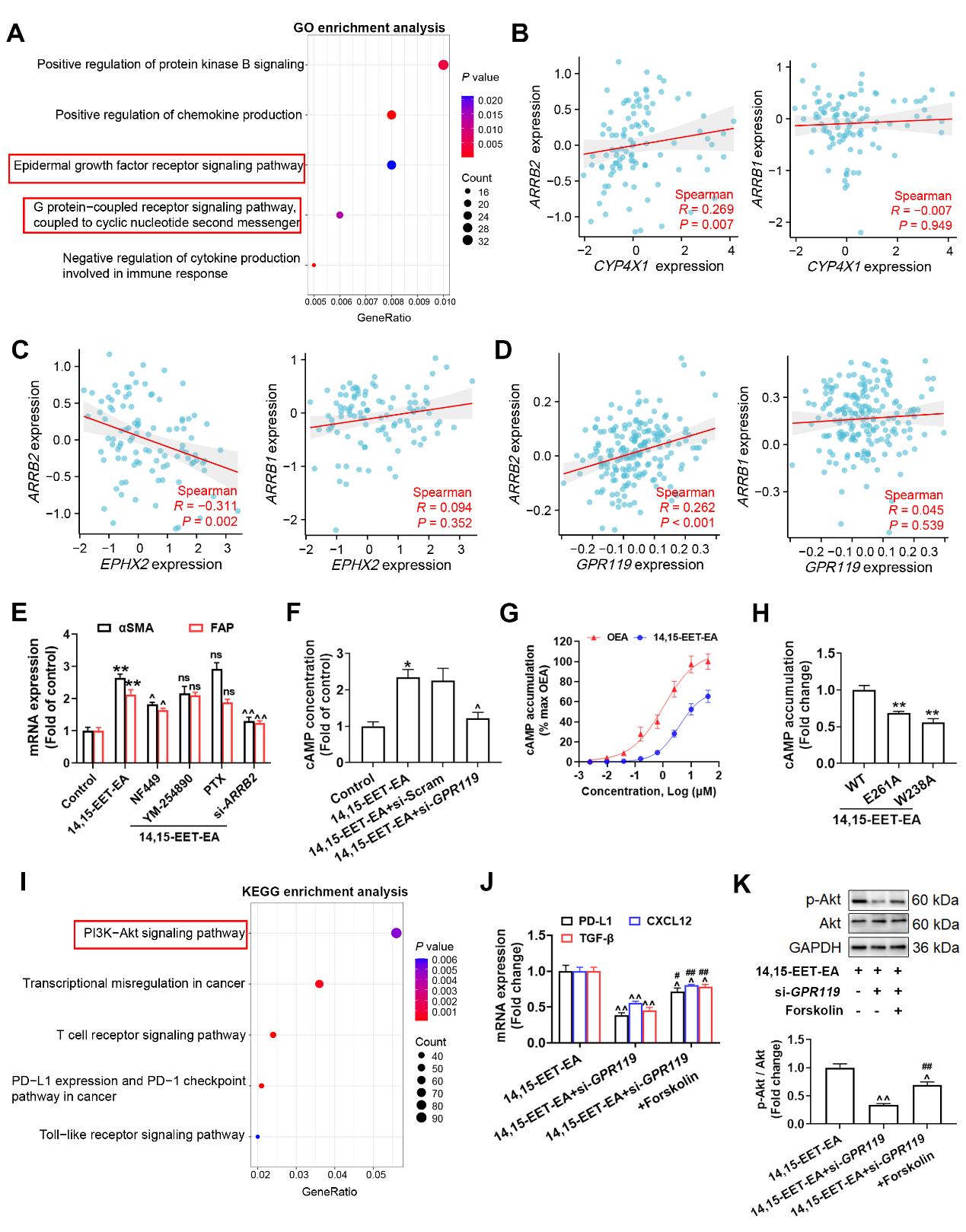


**Figure S14.** CYP4X1/sEH-derived 14,15-EET-EA mediates immunosuppression partially through the GPR119-Gs-cAMP pathway. A) GO enrichment analysis was conducted using the DEGs between *GPR119* high expression (*GPR119*^Hi^) and *GPR119* low expression (*GPR119*^Lo^) groups in the GSE31905 dataset. B,C) Spearman correlation analysis of *CYP4X1* and *EPHX2* with *ARRB2* and *ARRB1* in colon cancer patients (GSE33193). D) Spearman correlation analysis of *GPR119* with *ARRB1* and *ARRB2* in colon cancer patients (GSE42284). E) αSMA and FAP mRNA levels in MRC5 fibroblasts treated with Gs inhibitor (NF449), Gq inhibitor (YM-254890), Gi inhibitor (pertussis toxin, PTX), or *ARRB2* siRNA in the presence of 14,15-EET-EA were measured by qPCR. F) cAMP level in MRC5 fibroblasts with or without *GPR119* knockdown, followed by stimulation with 14,15-EET-EA. G) The GPR119 activation is tested by cAMP accumulation with OEA as a control. H) Agonistic activity of 14,15-EET-EA for wild-type GPR119 and mutant GPR119 measured by cAMP accumulation. I) KEGG enrichment analysis was conducted using the DEGs between the *GPR119*^Hi^ and *GPR119*^Lo^ groups in the GSE31905 dataset. J) PD-L1, CXCL12, and TGF-β mRNA levels in MRC5 fibroblasts transduced with *GPR119* siRNA and treated with or without forskolin (100 nM) in the presence of 14,15-EET-EA. K) p-Akt protein level in MRC5 fibroblasts of the indicated groups. Data are presented as mean ± SEM, *n* = 3. *P* values were determined using one-way ANOVA (E, F, H, J, and K). ^*^ *P* < 0.05 and ^**^ *P* < 0.01 vs. control or WT; ^^^ *P* < 0.05 and ^^^^ *P* < 0.01 vs. 14,15-EET-EA or 14,15-EET-EA + si-Scram; ^#^ *P* < 0.05 and ^##^ *P* < 0.01 vs. 14,15-EET-EA + si-*GPR119*; ns, not significant.


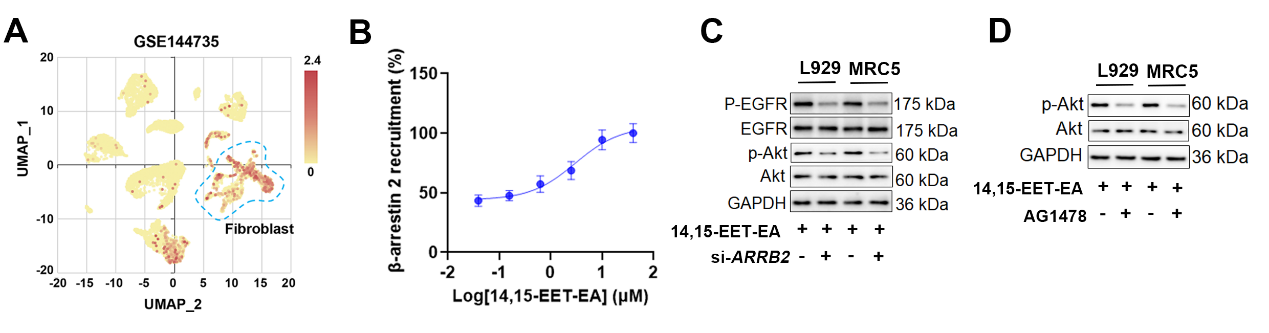


**Figure S15.** CYP4X1/sEH-derived 14,15-EET-EA mediates immunosuppression partially via GPR119-mediated EGFR transactivation. A) ScRNA-seq analysis of *EGFR* expression in human colorectal cancer tissues using the IMMUcan SingleCell RNAseq Database. B) 14,15-EET-EA dose-response curve in the PathHunter β-Arrestin recruitment assay for GPR119. C) The protein levels of p-EGFR and p-Akt in L929 and MRC5 fibroblasts treated with or without *ARRB2* siRNA in the presence of 14,15-EET-EA. D) The protein level of p-Akt in L929 and MRC5 fibroblasts treated with or without EGFR inhibitor AG1478, followed by stimulation with 14,15-EET-EA. Data are presented as mean ± SEM, *n* = 3.


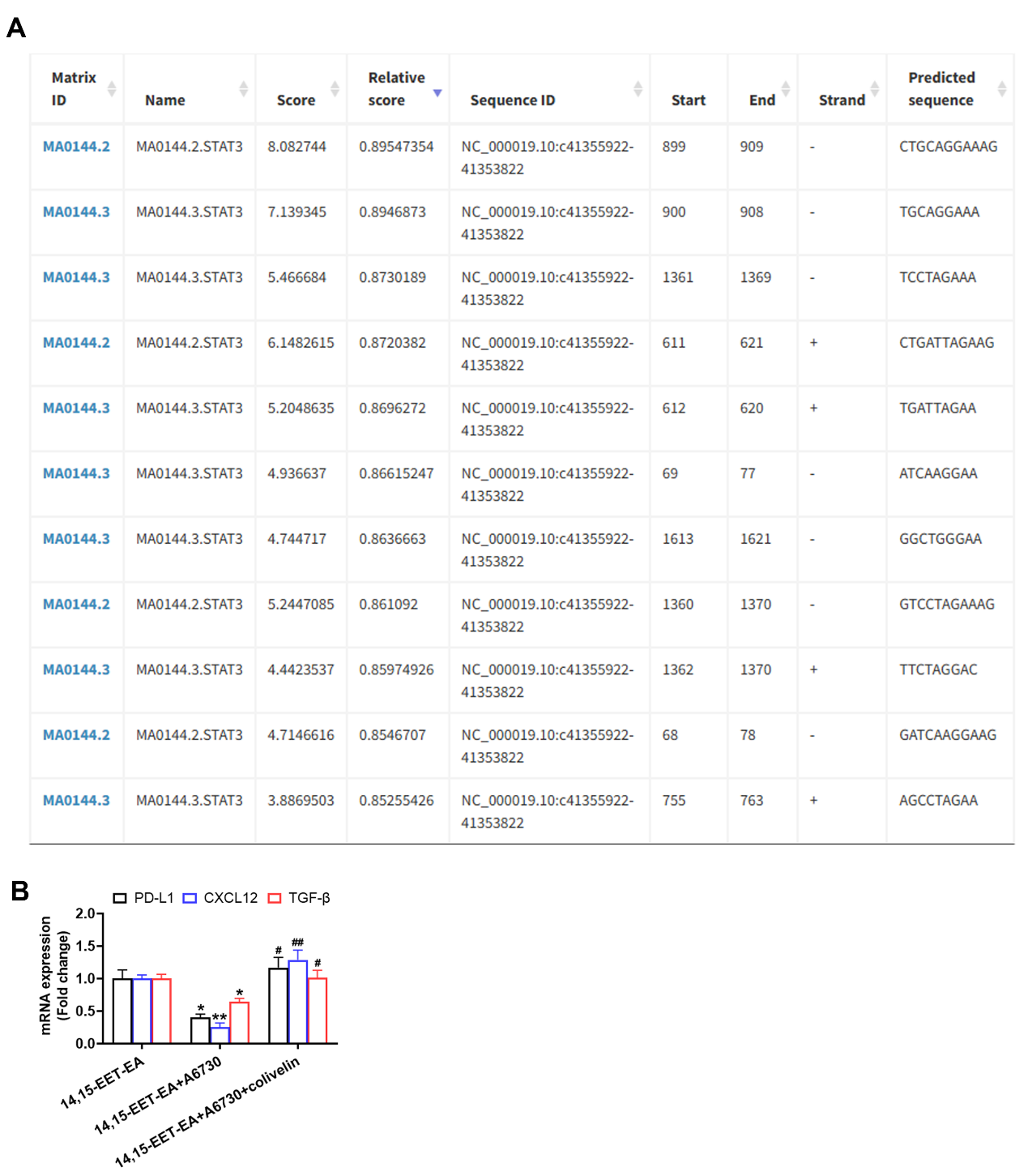


**Figure S16.** CYP4X1/sEH-derived 14,15-EET-EA upregulates PD-L1, CXCL12, and TGF-β in CAFs through PI3K/AKT/STAT3 signaling. A) The result chart of the prediction binding sites between STAT3 and the *TGFB1* promoter region by the JASPAR database. B) PD-L1, CXCL12, and TGF-β mRNA levels in MRC5 fibroblasts treated with A6730, colivelin, or their combination in the presence of 14,15-EET-EA. Data are presented as mean ± SEM, *n* = 3. *P* values were determined using one-way ANOVA. ^*^ *P* < 0.05 and ^**^ *P* < 0.01 vs. 14,15-EET-EA; ^#^ *P* < 0.05 and ^##^ *P* < 0.01 vs. 14,15-EET-EA + A6730.


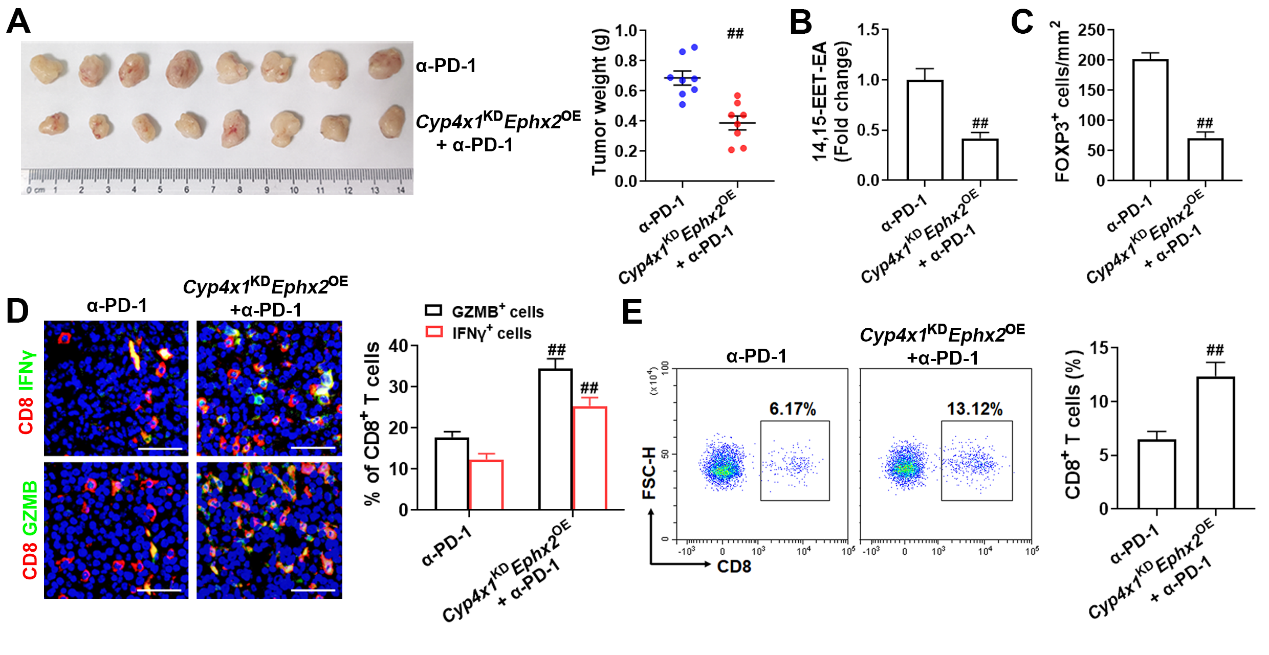


**Figure S17.** Targeting the regulation of CYP4X1 and EPHX2 potently enhances the efficacy of anti-PD-1 therapy in colon cancer. A) CT26 cells with or without *Cyp4x1*^KD^*Ephx2*^OE^ expression were subcutaneously implanted into BALB/c mice and treated with anti-PD-1 antibody. Tumor weights for the indicated groups were presented (*n* = 8). B) 14,15-EET-EA level was determined by LC-MS/MS in CT26 tumors. C) Treg accumulation in tumor tissues was analyzed and quantified by IHC staining. D) Representative IF staining and quantification of CD8 (red) and GZMB (green) or IFN-γ (green) in tumor tissues. Scale bar, 50 μm. E) The infiltration of CD8^+^ T cells in tumor tissues was determined by flow cytometry. Data are presented as mean ± SEM, *n* = 6. *P* values were determined using Student's t-tests. ^##^ *P* < 0.01 vs. α-PD-1 group.


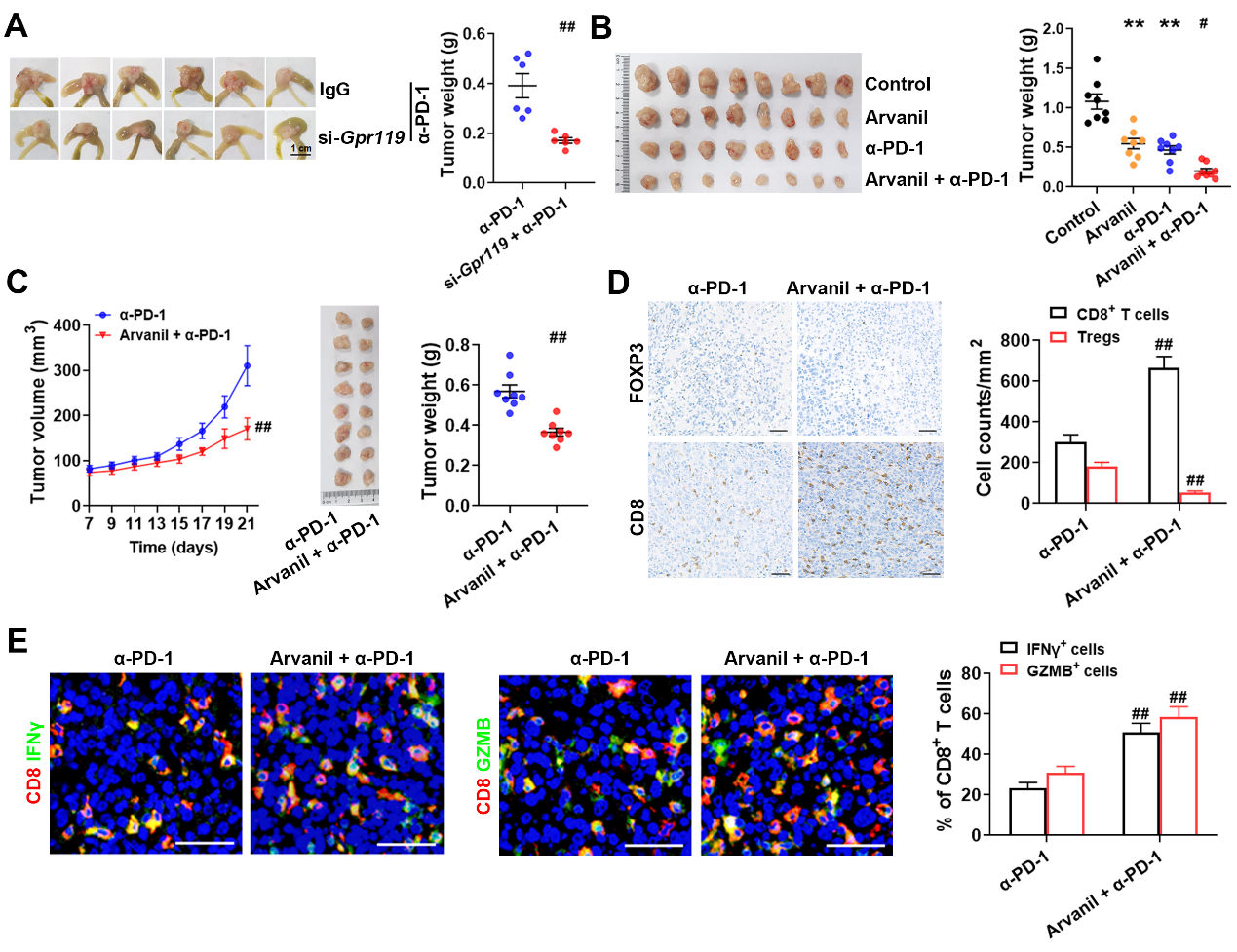


**Figure S18.** Targeted inhibition of GPR119 potently enhances the efficacy of anti-PD-1 therapy in colon cancer. A) C57BL/6 mice were orthotopically inoculated with MC38 cells and treated with anti-PD-1 or si-*Gpr119* plus anti-PD-1. Tumor weights were measured in the indicated groups (*n* = 6). B) C57BL/6 mice inoculated with MC38 cells were treated with arvanil, anti-PD-1 alone, or their combination. Tumor weights were measured in the indicated groups (*n* = 8). C) CT26 tumor growth curves (Two-way ANOVA) and tumor weights following treatment of arvanil or vehicle control in BALB/c mice treated with anti-PD-1 (*n* = 8). D) Representative IHC staining and quantitative analyses of CD8 and FOXP3 in tumor tissues treated with the indicated therapies (*n* = 6). Scale bar, 50 μm. E) Representative IF staining and quantification of CD8 (red) and IFN-γ (green) or GZMB (green) in tumor tissues (*n* = 6). Scale bar, 50 μm. Data are shown as mean ± SEM. *P* values were determined using Welch’s t test (A), one-way ANOVA (B), or Student's t-tests (C-E). ^**^ *P* < 0.01 vs. control; ^#^ *P* < 0.05 and ^##^ *P* < 0.01 vs. α-PD-1.


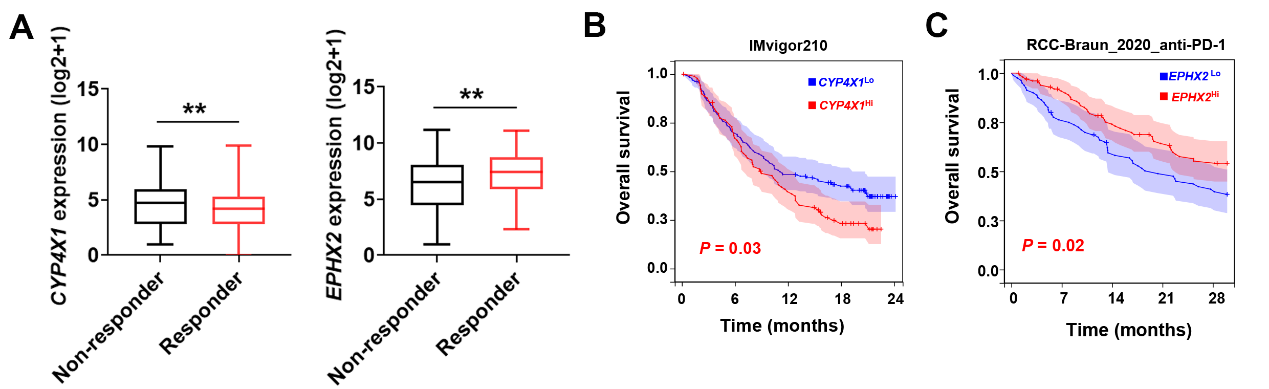


**Figure S19.** *CYP4X1* and *EPHX2* predict response to immunotherapy in human cancers. A) *CYP4X1* and *EPHX2* expression levels between responders and non-responders of patients with tumors who received PD-1 blockade therapy. B) Kaplan-Meier plots of overall survival for the patients according to the *CYP4X1* expression in the IMvigor210 cohort. C) Kaplan-Meier plots of overall survival for the patients according to the *EPHX2* expression in the RCC-Braun_2020 dataset. *P* values were determined using Mann-Whitney tests (A) or log-rank tests (B and C). ^**^ *P* < 0.01.


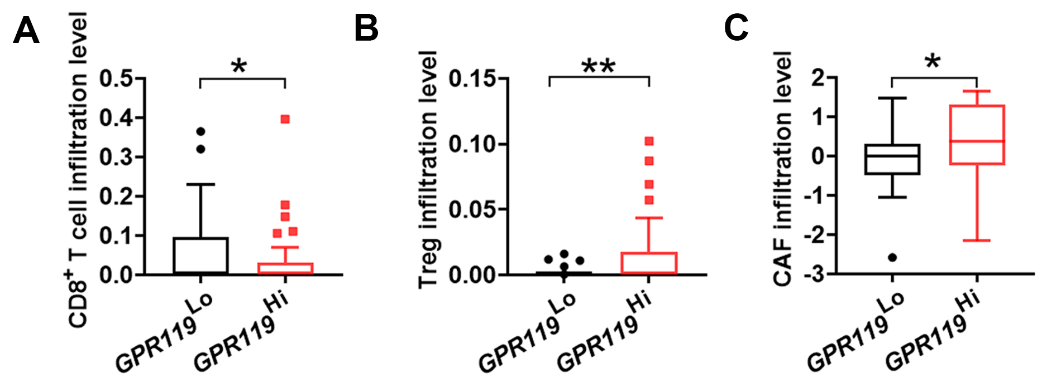


**Figure S20.** Correlation analysis of *GPR119* expression with immune and stromal cell infiltration. A-C) Correlation analysis of *GPR119* gene expression with immune and stromal cell infiltrations in the GEO database (GSE71187, GSE106582, and GSE31905). *P* values were determined using Mann-Whitney tests. ^*^ *P* < 0.05; ^**^ *P* < 0.01.


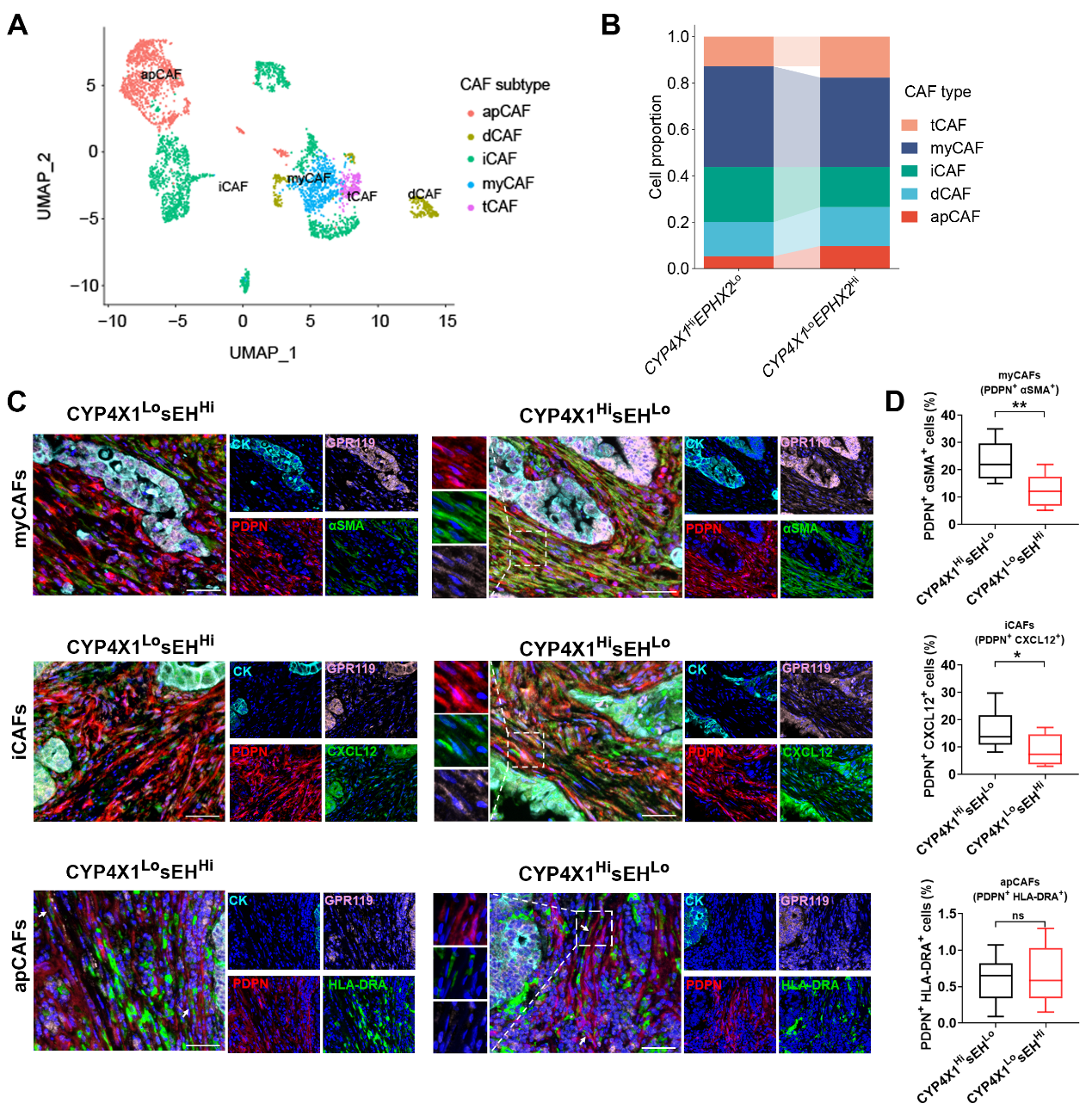


**Figure S21.** Correlation analysis of CYP4X1/sEH with CAF subtypes. A) UMAP showing identified CAF subtypes based on marker genes in the GSE132465 dataset. B) The proportion of CAF subtypes in *CYP4X1*^H^*^i^EPHX2*^Lo^ and *CYP4X1*^Lo^*EPHX2*^Hi^ groups. C) Representative images of mIF staining in human colon cancer tissues from CYP4X1^Lo^sEH^Hi^ and CYP4X1^Hi^sEH^Lo^ groups. D) Quantitative analysis of the proportions of myCAFs, iCAFs, and apCAFs. *P* values were determined using Student's t-tests. ^*^ *P* < 0.05; ^**^ *P* < 0.01; ns, not significant.





**Figure S22.** Correlation analysis of BMI with *CYP4X1*, *EPHX2*, and *GPR119* expression in colon cancer patients. A) *CYP4X1* gene expression level in human colon cancer tissues and normal tissues based on body mass index (BMI; TCGA). B) *EPHX2* gene expression level in human colon cancer tissues and normal tissues based on BMI (TCGA). C) *GPR119* gene expression level between the high- and low-BMI patients with colon cancer (TCGA). Data are shown as mean ± SEM. *P* values were determined using Mann-Whitney tests. ^**^ *P* < 0.01 vs. Normal or BMI ≤ 25.


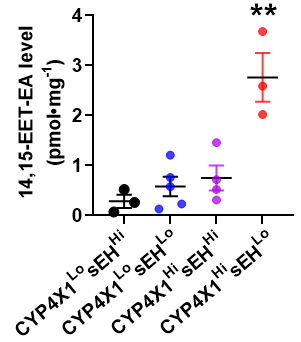


**Figure S23.** 14,15-EET-EA level in human colon cancer tissues. 14,15-EET-EA level was determined in four types of human colon cancer tissues (CYP4X1^Lo^sEH^Hi^, *n* = 3; CYP4X1^Lo^sEH^Lo^, *n* = 5; CYP4X1^Hi^sEH^Hi^, *n* = 4; CYP4X1^Hi^sEH^Lo^, *n* = 3) by LC-MS/MS. Data are presented as the mean ± SEM. *P* values were determined using one-way ANOVA. ^**^ *P* < 0.01 vs. CYP4X1^Lo^sEH^Hi^.


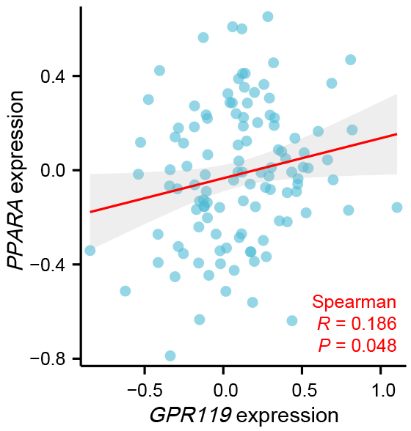


**Figure S24.** Spearman correlation analysis between *PPARA* and *GPR119* gene expression levels in colon cancer patients from the GSE75500 dataset.


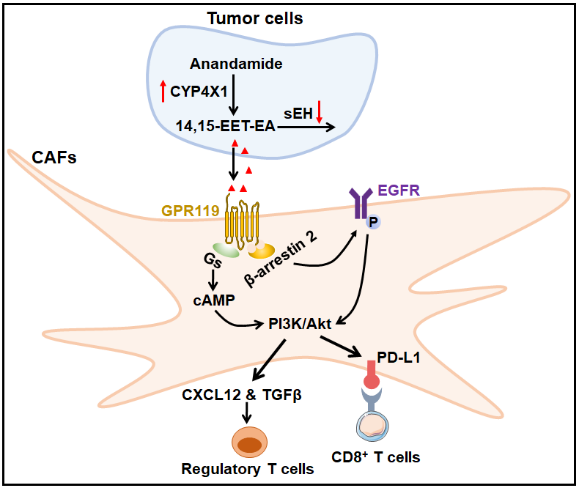


**Figure S25.** A proposed mechanism to explain the role of CYP4X1/sEH-14,15-EET-EA system in colon cancer immune escape.

**Supplementary Table S1.** Biochemical analyses of blood species from arvanil- or anti-PD-1-treated mice with MC38-derived colon cancer xenografts.

| Groups | Control | arvanil | α-PD-1 | arvanil+α-PD-1 |
| --- | --- | --- | --- | --- |
| Body weight [g] | 24.5 ± 0.4 | 23.1 ± 0.3 | 23.5 ± 0.4 | 23.9 ± 0.7 |
| ALT [IU/l] | 36.2 ± 2.1 | 39.1 ± 1.7 | 37.1 ± 1.6 | 40.6 ± 1.1 |
| BUN [mg/dl] | 23.8 ± 1.7 | 27.4 ± 2.0 | 25.0 ± 0.9 | 26.6 ± 1.9 |
| CREA [mg/dl] | 0.6 ± 0.1 | 0.6 ± 0.0 | 0.5 ± 0.0 | 0.7 ± 0.1 |
| AST [IU/l] | 110.0 ± 6.3 | 130.4 ± 9.5 | 120.4 ± 7.8 | 124.0 ± 8.4 |
| CK-MB [IU/l] | 101.7 ± 9.2 | 132.4 ± 11.8 | 117.2 ± 11.0 | 129.7 ± 10.7 |
| RBC [× 10^12^/l] | 8.7 ± 0.2 | 9.0 ± 0.4 | 8.6 ± 0.3 | 9.4 ± 0.3 |
| WBC [× 10^9^/l] | 3.8 ± 0.2 | 4.5 ± 0.3 | 4.1 ± 0.3 | 4.7 ± 0.4 |
| HCT [%] | 41.6 ± 1.5 | 46.7 ± 1.4 | 42.4 ± 1.9 | 43.6 ± 1.7 |
| MCV [fl] | 48.0 ± 1.4 | 44.6 ± 1.6 | 47.3 ± 1.3 | 45.0 ± 1.4 |
| PLT [× 10^9^/l] | 753.4 ± 10.4 | 762.3 ± 15.0 | 723.6 ± 21.8 | 717.2 ± 14.8 |

Abbreviations: ALT, alanine aminotransferase; BUN, blood urea nitrogen; CREA, creatine; AST, aspartate aminotransferase; CK-MB, creatine kinase-MB; RBC, red blood corpuscles; WBC, white blood corpuscles; HCT, hematocrit; MCV, mean cell volume; PLT, platelet. The values are presented as the mean ± standard error of the mean, *n* = 8.

**Supplementary materials and methods**

**Chemicals and reagents**

14,15-epoxyeicosatrienoic acid-ethanolamide (14,15-EET-EA) (#10008599, purity ≥ 95%) was purchased from Cayman Chemicals (Ann Arbor, MI). Rat monoclonal antibody to mouse PD-1 (#BE0146) was obtained from BioXCell (West Lebanon, NH, USA). Tranilast and AG1478 were acquired from TargetMol (Massachusetts, USA). Forskolin was obtained from MedChemExpress (Monmouth Junction, NJ, USA). A6730 was purchased from Sigma Chemical Co. (St. Louis, MO, USA). Arvanil was obtained from Tocris Bioscience (Bristol, UK). Opal Polaris 7 Color IHC Detection Kit (#NEL861001KT) was purchased from Akoya Bioscience (Menlo Park, CA, USA).

**Cell culture**

Human colon cancer HT29 and HCT116 cell lines, C57BL/6-derived MC38 colon carcinoma cells, BALB/c-derived CT26 colon carcinoma cells, human fetal lung fibroblast cell line MRC5, mouse fibroblast cell line L929, and human umbilical vein endothelial cells were purchased from the ATCC. Human brain vascular pericytes were purchased from ScienCell Research Laboratories. Human CD8^+^ T cells and Tregs were isolated from the human peripheral blood mononuclear cells (PBMCs) of healthy donors in accordance with the Helsinki Declaration. All volunteers were healthy nonsmoking males with written informed consent, and the study was approved by the Ethical Committee of the Medical School of Wuhan University. All cells were cultured in RPMI 1640 or DMEM medium supplemented with 10% fetal bovine serum and maintained at 37°C in an atmosphere of 5% carbon dioxide. For all cell lines, mycoplasma testing using the MycoAlert Mycoplasma Detection kit (Lonza, Slough, UK) was performed every 3 months. Cells were regularly assessed based on their morphology before use.

**Enzyme-linked immunosorbent assay (ELISA)**

TGF-β and CXCL12 in the culture supernatants from MRC5 fibroblasts were measured using ELISA kits (R&D Systems) according to the manufacturer's protocol.

**Immunohistochemistry and Immunofluorescence staining**

Paraffin-embedded tissues from human colon tumors or xenograft tumors were processed and stained following standard protocols. In brief, the paraffin-embedded slides were deparaffinized, rehydrated, and then subjected to antigen retrieval in a pH 6.0/pH 9.0 buffer using microwave treatment. After the inactivation of endogenous peroxidase, slides were blocked with 10% normal goat serum. Subsequently, slides were incubated overnight with different primary antibodies against GPR119 (1:100, Affinity, #DF4892), α-SMA (1:300, BOSTER, #BM0002), FOXP3 (1:200, Cell Signaling Technology, #12653), CD31 (1:300, Santa Cruz, #sc-376764), and CD8 (1:400, Cell Signaling Technology, #98941). After washing, slides were incubated with polymer HRP-goat anti-rabbit/mouse secondary antibody and detected using DAB chromogenic reagent. The nuclei were stained with hematoxylin. For immunofluorescence staining, all specimens were incubated with different primary antibodies against CD31 (1:300, Santa Cruz, #sc-376764), NG2 (1:200, Abcam, #ab129051), α-SMA (1:300, BOSTER, #BM0002), vimentin (1:200, Abcam, #ab92547), CD8 (1:200, Santa Cruz, #sc-1177), granzyme B (1:200, Abcam, #ab255598), or IFN-γ (1:200, Abcam, #ab231036) overnight at 4°C for colocalization detection. After washing, the sections were incubated with a mixture of Alexa Fluor 488- and Alexa Fluor 594-conjugated secondary antibodies for 1 h at room temperature in the dark. Finally, the slides were counterstained with 4′,6-diamidino-2-phenylindole (DAPI) to visualize the nuclei. Each section was observed using an Olympus BX51 microscope with CCD DP80 (Olympus, Tokyo, Japan).

**Tumor conditioned medium (CM) preparation**

Tumor conditioned medium (CM) was prepared as previously described.^[1]^ In brief, *CYP4X1*^KD^-, *EPHX2*^OE^-, or *CYP4X1*^KD^*EPHX2*^OE^-expressed colon cancer cell lines (HCT116 and HT29) at about 80% confluence were cultured in serum-free medium for 24 h. The culture supernatants of tumor cells from each group were collected and centrifuged at 1000 × g for 5 min to obtain tumor-primed medium. The CM was obtained by mixing the tumor-primed medium with regular medium (v/v = 1:1). Fibroblasts were then cocultured with the CM for 24 h *in vitro*.

**Flow cytometry analysis**

Mouse tumor tissues were finely minced and incubated in RPMI-1640 medium containing 5% fetal bovine serum (FBS), 1.5 mg/mL collagenase IV (Sigma, #C5138), and 0.1 mg/mL DNase I (Sigma-Aldrich, #DN25) at 37°C for 45 min, and then passed through 70 μm filters (BD) to obtain single-cell suspensions. All single cells were treated with Red Cell lysis buffer for 1 min at room temperature to lyse erythrocytes. The isolated cells were incubated with anti-mouse CD16/32 antibody (Biolegend, #101319) for 10 min on ice to block Fc-mediated reactions, followed by staining with the following fluorochrome-conjugated antibodies or isotype control for 30 min at 4°C: anti-CD45-APC/Cy7 (BD Biosciences, #557659), anti-CD4-FITC (Biolegend, #121606), anti-CD8-APC (BD Biosciences, #553035), and anti-CD107a-FITC (Biolegend, #121606). For intracellular staining, single-cell suspensions from tumors were pre-incubated in medium containing cell activation cocktail (Biolegend, #423301) and protein transport inhibitor monensin (Biolegend, #420701) for 4 h at 37°C before surface staining. After that, cells were fixed and permeabilized in Fixation/Permeabilization diluent (BD Biosciences, #562574) for intracellular staining of anti-IFNγ-PE-Cy7 (BD Biosciences, #557649) and anti-FOXP3-PE (Biolegend, #126404). All data were acquired on a flow cytometer (CytoFLEX S, Beckman Coulter) and analyzed with FlowJo software.

For the CD8^+^ T cell suppression assay, human peripheral blood mononuclear cells (PBMCs) were isolated from the blood of healthy donors by Ficoll density gradient centrifugation (Cytiva, #17544602), and CD8^+^ T cells were purified from PBMCs using EasySep enrichment kits (STEMCELL). Enriched naive CD8^+^ T cells were stimulated with 2 μg/ml plate-bound anti-CD3 and anti-CD28 antibodies for 48 h, and then labeled with carboxyfluorescein succinimidyl ester (CFSE; TargetMol). CFSE-labeled CD8^+^ T cells were co-cultured with MRC5 fibroblasts stimulated by CM from the *CYP4X1*^KD^-, *EPHX2*^OE^-, or *CYP4X1*^KD^*EPHX2*^OE^-expressed HCT116 cells for 48 h in the presence of anti-CD3 and anti-CD28 antibodies. CFSE was determined by flow cytometry to assess CD8^+^ T cell proliferation.

**Migration assays**

For the Treg migration assay, Tregs were isolated from the PBMCs of healthy donors according to the manufacturer’s instructions (Miltenyi Biotec, #130-091-301). Tregs were seeded to the upper chamber of 5-μm pore size Transwell inserts (Corning). MRC5 fibroblasts stimulated by CM from indicated colon cancer cells with *CYP4X1*^KD^, *EPHX2*^OE^, or *CYP4X1*^KD^*EPHX2*^OE^ expression were inoculated for 24 h on the bottom chamber of the transwell. After 24 h of co-incubation at 37˚C under 5% CO_2_, the number of migrated Tregs in the lower chamber was enumerated by flow cytometry.

For pericyte and endothelial cell migration assays, the CM from the MRC5 fibroblasts with the indicated treatment was added to the lower chamber. Pericytes (human brain vascular pericytes) or endothelial cells (HUVECs) were added to the upper chamber. Cells were incubated overnight to assess their ability to migrate across the 8-μm transwell insert toward the lower chamber. The number of cells that migrated to the lower chamber was counted.

**Lentiviral transfection**

HCT116, HT29, MC38, and CT26 cells were infected with EPHX2 lentiviral activation particles (Santa Cruz) and/or CYP4X1 shRNA lentivirus (Santa Cruz) in 6-well tissue culture plates for 24 h. To generate a stably transfected population, medium supplemented with the appropriate selection agent (puromycin dihydrochloride, hygromycin B, or blasticidin S HCl, all Life Technologies) was added and replaced every 3-4 days until resistant cells could be identified. Western blot was used to verify the efficiency of overexpression or knockdown.

**Isolation of mouse CAFs**

Mouse CAFs were isolated from MC38 or CT26 tumors. CAFs were isolated using the outgrowth isolation method according to a previous study.^[2]^ Briefly, excised tumors were finely minced and incubated for 2 h at 37°C in a digestion solution composed of collagenase type XI (0.5 mg/ml, Sigma Aldrich), dispase (0.2 mg/ml, Gibco), and 1% fetal bovine serum in RPMI 1640 medium before passage through a 70 μm cell strainer (BD) to obtain single-cell suspensions. The pellet was resuspended in serum-free RPMI 1640 medium, followed by purification using the differential time adherent method. The established cell culture was passaged for at least 3 generations to eliminate potential tumor cell contamination.

**AEA, OEA, PEA, and 14,15-EET-EA content measurement**

AEA, OEA, PEA, and 14,15-EET-EA were measured by LC-MS/MS as previously described.^[3]^ Briefly, aliquots (10 μL) of the sample extracts were injected onto a Discovery® HS C18 column (15 cm × 2.1 mm, 3 μm) with a flow rate of 1.0 mL/min. The mobile phase comprised (A) water containing 1 g/L ammonium acetate and 0.1% formic acid, and (B) acetonitrile. The gradient elution program was set as follows: initial condition was 40% B; from 2.5 to 6.0 min, the proportion of B was linearly increased to 60% and maintained for 2.1 min; from 8.1 to 9.0 min, B was raised to 100% and held for 3.1 min; at 12.1 min the mobile phase was return to 40% B. Mass spectra were acquired under positive electrospray ionization (ESI). The molecular ions were monitored at m/z 348.2/62.2 for AEA, m/z 326.0/62.0 for OEA, m/z 300.0/62.0 for PEA, and m/z 364.0/62.0 for 14,15-EET-EA.

**Real-time quantitative PCR**

Total cellular RNA was extracted using the TRIzol reagent (TIANGEN), and reverse-transcribed to cDNA using the HiScript III 1st Strand cDNA Synthesis Kit (Vazyme, R312-01) according to the manufacturer’s instructions. Subsequently, the resulting cDNA was used for real-time PCR utilizing SYBR Green PCR Master Mix (Vazyme, #Q121-02) on a CFX96 Real-Time System (Bio-Rad, Hercules, CA). The relative RNA expression levels were normalized to β-actin according to the 2^-ΔΔCt^ calculation method. Forward (F) and reverse (R) primers used were listed as follows: Human-TGF-β-F 5′-CCC ACA ACG AAA TCT ATG ACA AG-3′, Human-TGF-β-R 5′-CTA AGG CGA AAG CCC TCA AT-3′; Human-α-SMA-F 5′-GAC CGA ATG CAG AAG GAG AT-3′, Human-α-SMA-R 5′-CCA CCG ATC CAG ACA GAG TA-3′; Human-CXCL12-F 5′-ATT CTC AAC ACT CCA AAC TGT GC-3′, Human-CXCL12-R 5′-ACT TTA GCT TCG GGT CAA TGC-3′; Human-GZMB-F 5′-TGA AGC CAG GGC AGA CAT GC-3′, Human-GZMB-R 5′-GCC TCC AGA GTC CCC CTT AA-3′; Human-IFN-γ-F 5′-TGA CCA GAG CAT CCA AAA GA-3′, Human-IFN-γ-R 5′-CTC TTC GAC CTC GAA ACA GC-3′; Human-FAP-F 5′-GGG ATG GTC ATT GCC TTG GT-3′, Human-FAP-R 5′-CTC CAT AGG ACC AGC CCC ATA-3′; Human-PD-L1-F 5′-TGG CAT TTG CTG AAC GCA TTT-3′, Human-PD-L1-R 5′-TGC AGC CAG GTC TAA TTG TTT T-3′; Human-β-actin-F 5′-AAG ATC ATT GCT CCT CCT GA-3′; Human-β-actin-R 5′-CTC GTC ATA CTC CTG CTT GCT-3′; Mouse-TGF-β-F 5′-TGA GTG GCT GTC TTT TGA CG-3′, Mouse-TGF-β-R 5′-TCT CTG TGG AGC TGA AGC AA-3′; Mouse-PD-L1-F 5′-GAC GCA GGC GTT TAC TGC T-3′, Mouse-PD-L1-R 5′-GCG GTA TGG GGC ATT GAC TTT-3′; Mouse-CXCL12-F 5′-TGC ATC AGT GAC GGT AAA CCA-3′, Mouse-CXCL12-R 5′-CAC AGT TTG GAG TGT TGA GGA T-3′; Mouse-α-SMA-F 5′- CCA GAG CAA GAG AGG GAT CCT-3′, Mouse-α-SMA-R 5′-TGT CGT CCC AGT TGG TGA TG-3′; Mouse-FAP-F 5′-CAC CTG ATC GGC AAT TTG TG-3′, Mouse-FAP-R 5′-CCC ATT CTG AAG GTC GTA GAT GT-3′; Mouse-β-actin-F 5′-CAT CCG TAA AGA CCT CTA TGC CAA C-3′, Mouse-β-actin-R 5′-ATG GAG CCA CCG ATC CAC A-3′.

**Western blot analysis**

Protein extracts were separated by SDS-PAGE and then transferred to polyvinylidene fluoride membranes. After that, membranes were blocked in TBST containing 5% nonfat milk for 2 h at room temperature and then incubated overnight at 4˚C with the indicated primary antibodies against CYP4X1 (1:500, Invitrogen, #PA5-101319), sEH (1:500, Proteintech, #10833-1-AP), GPR119 (1:500, Affinity, #DF4892), β-Arrestin 2 (1:1000, Cell Signaling Technology, #3857), p-EGFR (1:1000, Abcam, #ab40815), EGFR (1:1000, Abcam, #ab52894), p-AKT (1:1000, Cell Signaling Technology, #9271), AKT (1:1000, Abcam, #ab8805), or GAPDH (1:5000, Abcam, #ab8245). After washing, the membranes were incubated with secondary antibodies. Protein bands were visualized using an ECL kit (YEASEN), and the intensity of the bands was quantified by densitometric analysis using ImageJ software.

**cAMP measurement**

The cAMP assays were performed in HEK293 cells transfected with GPR119. Cells were collected and resuspended in phosphate buffer saline (PBS) containing 0.5 mM 3-isobutyl-1-methylxanthine (IBMX). Cells were then plated onto 96-well assay plates at 1 × 10^3^ cells per 10 µl per well. Another 10 µl of PBS containing different concentrations of compounds (14,15-EET-EA and OEA) was added to the cells, and the incubation lasted for 30 min at 37 °C. Intracellular cAMP levels were measured using the LANCE Ultra cAMP kit (PerkinElmer, TRF0264) following the manufacturer’s instructions, with detection performed on a multimode microplate reader (Tecan SPARK 10 M).

CAFs were cultured and treated with *CYP4X1*^KD^*EPHX2*^OE^ CM with or without 14,15-EET-EA. The cAMP level was measured by using a cAMP enzyme-linked immunosorbent assay (ELISA) kit (Abcam, ab234585) according to the manufacturer’s protocol.

**Molecular docking**

The crystal structure of the human GPR119 (PDB ID: 7WCN) was obtained from the Research Collaboratory for Structural Bioinformatics Protein Data Bank (RCSB PDB, http://www.rcsb.org/pdb/), and the standard structure of 14,15-EET-EA (PubChem CID: 24778498) was retrieved from the PubChem Compound Database (https://pubchem.ncbi.nlm.nih.gov/). Molecular docking was carried out using SYBYL-X 2.0 software, and the docking result was visualized with PyMOL and LigPlot software.

**Cellular thermal shift assay**

Cellular thermal shift assay (CETSA) was conducted according to a previously described protocol.^[4]^ Briefly, MC38 CM-incubated L929 cells were pretreated with 14,15-EET-EA or vehicle for 1 h. After collection, the cells were heated at each temperature point from 36 to 72 °C for 3 min and then lysed using the freezing and thawing method. The supernatant obtained after centrifugation was subjected to western blot analysis to confirm the thermal stability of GPR119.

**β-arrestin recruitment experiment**

The PathHunter β-arrestin assay (DiscoverX) was used to measure β-arrestin 2 recruitment according to the manufacturer’s protocol. In brief, PathHunter U2OS GPR119 β-Arrestin cells were seeded in a total volume of 90 µL into 96-well microplates and incubated overnight at 37°C and 5% CO_2_ prior to the test. Stimulation with different concentrations of 14,15-EET-EA was performed for 90 min at 37°C, followed by incubation with the Detection Reagent Solution for 1 h. The chemiluminescence was measured using a multimode microplate reader.

**Bioinformatics analysis**

Data on gene expression and clinicopathologic characteristics of colon cancer patients were downloaded from the TCGA and Gene Expression Omnibus (GEO) databases (GSE39582 and GSE44076). According to the setting cut-off threshold (*P*-value < 0.05 and |log2(fold change)|> 1), the differentially expressed genes (DEGs) between the tumor and normal tissue samples in TCGA-COAD and COAD-related GEO datasets were identified using the “edgeR” package in R. The Jvenn online tool (<https://jvenn.toulouse.inrae.fr/app/example.html>) was used to perform the intersection analysis of DEGs (colon cancer tissues vs. normal tissues) and endocannabinoid oxidative metabolism-related genes. The Cancer Single-cell Expression Map database (<https://ngdc.cncb.ac.cn/cancerscem/index>) or the IMMUcan SingleCell RNAseq Database (https://immucanscdb.vital-it.ch/) was employed to assess the single-cell expression of *CYP4X1*, *EPHX2*, and *EGFR* in human colon cancer tissues. Gene Ontology (GO) enrichment analysis was performed with the DEGs between *CYP4X1*^Hi^*EPHX2*^Lo^ and *CYP4X1*^Lo^*EPHX2*^Hi^ groups by using the “clusterProfiler” R package in the TCGA-COAD dataset. The ROC plotter database (https://rocplot.org/immune) was utilized to explore the relationship of *CYP4X1* and *EPHX2* expression with immunotherapy responses in cancer patients. The Kaplan-Meier plotter online database (<http://kmplot.com/analysis/>) was used to analyze the effects of *CYP4X1* and *EPHX2* expression on the overall survival and progression-free survival in cancer patients receiving anti-PD-1 therapy. GMrepo (<https://gmrepo.humangut.info/home>) and gutMEGA (<http://gutmega.omicsbio.info/browse.php>) databases were utilized to obtain differential intestinal floras in the stool samples of the healthy individuals and colorectal cancer patients. The patients in the TCGA and GEO databases were stratified into high- and low-expression groups for *CYP4X1*, *EPHX2*, and *GPR119*, based on the median expression levels of each gene, respectively.

**Reference**

[1] C. Hu, W. Qiao, X. Li, Z.-K. Ning, J. Liu, S. Dalangood, H. Li, X. Yu, Z. Zong, Z. Wen, J. Gui, *Cell Metab* **2024**, *36*, 630.

[2] M. Panagi, F. Mpekris, C. Voutouri, A. G. Hadjigeorgiou, C. Symeonidou, E. Porfyriou, C. Michael, A. Stylianou, J. D. Martin, H. Cabral, A. Constantinidou, T. Stylianopoulos, *Clin. Cancer Res*. **2024**, 30, 2582.

[3] S. K. Dempsey, A. M. Gesseck, A. Ahmad, Z. Daneva, J. K. Ritter, J. L. Poklis, *J. Chromatogr. B* **2019**, *1126–1127*, 121748.

[4] M. Oh, S. Y. Jang, J.-Y. Lee, J. W. Kim, Y. Jung, J. Kim, J. Seo, T.-S. Han, E. Jang, H. Y. Son, D. Kim, M. W. Kim, J.-S. Park, K.-H. Song, K.-J. Oh, W. K. Kim, K.-H. Bae, Y.-M. Huh, S. H. Kim, D. Kim, B.-S. Han, S. C. Lee, G.-S. Hwang, E.-W. Lee, *Nat. Commun.* **2023**, *14*, 5728.
